# Fungal communities in feces of the frugivorous bat *Ectophylla alba* and its highly specialized *Ficus colubrinae* diet

**DOI:** 10.1186/s42523-022-00169-w

**Published:** 2022-03-18

**Authors:** Priscila Chaverri, Gloriana Chaverri

**Affiliations:** 1 Escuela de Biología and Centro de Investigaciones en Productos Naturales (CIPRONA), Universidad de Costa Rica, San Pedro, Costa Rica; 2 Department of Plant Science and Landscape Architecture, University of Maryland, College Park, MD USA; 3 Sede del Sur, Universidad de Costa Rica, Golfito, 60701 Costa Rica; 4 Smithsonian Tropical Research Institute, Balboa, Ancón, Panamá

**Keywords:** Dispersal, Ecological network, Endophytes, Epiphytes, Gut microbiome, Janzen–Connell hypothesis, Metabarcoding, Mycobiota, *Pyxidiophora*, Theory of pest pressure

## Abstract

**Background:**

Bats are important long-distance dispersers of many tropical plants, yet, by consuming fruits, they may disperse not only the plant’s seeds, but also the mycobiota within those fruits. We characterized the culture-dependent and independent fungal communities in fruits of *Ficus colubrinae* and feces of *Ectophylla alba* to determine if passage through the digestive tract of bats affected the total mycobiota.

**Results:**

Using presence/absence and normalized abundance data from fruits and feces, we demonstrate that the fungal communities were significantly different, even though there was an overlap of ca. 38% of Amplicon Sequence Variants (ASVs). We show that some of the fungi from fruits were also present and grew from fecal samples. Fecal fungal communities were dominated by *Agaricomycetes*, followed by *Dothideomycetes*, *Sordariomycetes, Eurotiomycetes*, and *Malasseziomycetes*, while fruit samples were dominated by *Dothideomycetes*, followed by *Sordariomycetes*, *Agaricomycetes, Eurotiomycetes*, and *Laboulbeniomycetes*. Linear discriminant analyses (LDA) show that, for bat feces, the indicator taxa include *Basidiomycota* (i.e., *Agaricomycetes: Polyporales* and *Agaricales*), and the ascomycetous class *Eurotiomycetes* (i.e., *Eurotiales*, *Aspergillaceae*). For fruits, indicator taxa are in the *Ascomycota* (i.e., *Dothideomycetes*: *Botryosphaeriales*; *Laboulbeniomycetes*: *Pyxidiophorales*; and *Sordariomycetes*: *Glomerellales*). In our study, the differences in fungal species composition between the two communities (fruits vs. feces) reflected on the changes in the functional diversity. For example, the core community in bat feces is constituted by saprobes and animal commensals, while that of fruits is composed mostly of phytopathogens and arthropod-associated fungi.

**Conclusions:**

Our study provides the groundwork to continue disentangling the direct and indirect symbiotic relationships in an ecological network that has not received enough attention: fungi-plants-bats. Findings also suggest that the role of frugivores in plant-animal mutualistic networks may extend beyond seed dispersal: they may also promote the dispersal of potentially beneficial microbial symbionts while, for example, hindering those that can cause plant disease.

**Supplementary Information:**

The online version contains supplementary material available at 10.1186/s42523-022-00169-w.

## Introduction

Ecological networks have been the topic of extensive research, yet it seems that we still have important missing pieces of these complex puzzles [1]. For example, few studies have addressed the role of parasites in networks [2], while indirect interactions—those that occur when the interaction between two species is modified by a third one [3, 4]—are seldom addressed and poorly understood [5, 6]. Notwithstanding, indirect interactions are ubiquitous in ecological networks and play a critical role in shaping the bonds among species within communities [7–11]. While we typically believe that the survival of a species depends solely on the protection of its direct interactions, realistically it may also depend on how those interactions are shaped by species indirectly linked to each node in the network; the loss of any of these indirect links may have unforeseen effects on network functioning. Therefore, in considering the vulnerability of many species due to human activities, such as introduction of exotic species, habitat loss, introduction of parasites, and climate change, we need to acknowledge both the direct and indirect interactions of a taxon within ecological networks to fully understand the potential effects of its loss.

A type of ecological network that has received much attention is that which encompasses interactions between plants and their animal pollinators and seed dispersers; the interaction is mutually beneficial because animals help transport pollen and seeds, and in exchange obtain food (reviewed in [12]). Studies of plant-animal mutualistic networks have focused on understanding the relationship among interacting species [13, 14], the importance of particular taxa and network topology for maintaining network resilience [15–18], and how the topology of mutualistic networks may influence resilience [15–17] and even species diversity [15, 19]. However, these studies have neglected an important component of plant communities: their microbial symbionts. One such group of important microbial symbionts are endophytic fungi, which live within aerial tissues of plants without causing any visible negative impact. Even though some endophytes may be latent pathogens or saprotrophs [20], in many cases these endosymbionts provide benefits to the plant including protection against diseases and pests, plant growth, and reduction of drought stress [21]. As a result, endophytic fungi are regarded as critical components of any healthy plant community. Less is known about epiphytic or phyllosphere fungi, but there is some evidence of benefits to the host plant [22, 23].

The ability of a fungal species to disperse its spores (or other propagules such as hyphae, chlamydospores, and fruiting structures) is one of the factors that influence fungal diversity in a natural ecosystem [24, 25]. Fungi, including those that can become endophytic, can only disperse their spores short distances (a few centimeters at the most) using their own means (e.g., forcible ejection or discharge) [26, 27]. Consequently, they rely on other mechanisms for long distance dispersal, e.g., water, wind, and animals. Wind has been reported as the most efficient long-distance spore dispersal mechanism. However, in natural forests, especially old-growth, wind may not have a large influence in spore dispersal because trees and understorey vegetation provide a barrier to wind movement [28, 29]. Therefore, it is expected that other factors besides wind are influencing long-distance dispersal of fungi in natural tropical forests. Considering that animals are capable of long-distance dispersal of plant seeds, it is possible that their role extends to the dispersal of fungal spores and other propagules.

Many animals may disperse fungi directly by eating mushrooms and then defecating the spores; or indirectly, by eating other plant parts that contain these fungi. The direct consumption of fungal fruiting bodies, or mycophagy, and spore dispersal has been described several times in insects, rodents, marmosets, and other mammals [30–32]. In some cases it was reported that fungal spores survive and their germination is improved after passing the digestive tract of truffle-eating rodents or other ground-dwelling animals [32]. However, indirect fungal propagule dispersal is woefully unknown. We use bats as a model to understand this interaction because these mammals are important long-distance dispersers of many tropical plants [33]. Yet by consuming fruits, bats may disperse not only the plant’s seeds, but also the fungi that are contained in those fruits. Bats may be particularly good dispersers of fungi because they fly long distances each night, defecate during flight, and may retain viable propagules for long periods of time. Unlike birds, fruit-eating bats also venture frequently into deforested areas that may otherwise lack input of beneficial fungal spores [34–37]. This study represents a first step towards identifying an interaction that may have consequences for the preservation of healthy tropical ecosystems.

In this study we aimed to explore the hypothesis of an indirect mutualistic relationship between fruit-eating animals, specifically bats, and symbiotic fungi (with emphasis on endophytes) that grow within the tissues of fruits that bats eat. Since fungi can develop in any plant tissue as endophytes, including fruits [38, 39], it is presumed that bats may disperse fungal propagules that are consumed from these structures. To begin to investigate the poorly examined premise that bats are also long-distance dispersers of fungi, the main objectives of this study were to (i) characterize the fungal communities from the fruits of *Ficus colubrinae* and determine whether the same species are present in the feces of *Ectophylla alba*; and (ii) determine if at least some fungi survive the digestive tract of bats. In this project we aim to address these basic objectives, yet many questions will remain regarding the relationship between frugivores, plants, and fungi. We hope our answers will begin to shed light on this potential interaction and hopefully foster further scrutiny.

## Results

### *Ectophylla alba*’s main diet

Out of the total plant ITS nrDNA sequences amplified from the fecal samples, 82% (σ = 17) matched to several *Ficus* spp. with percent similarities of 90–99%, including *F. colubrinae* with 96% (GenBank accession number EU081760). However, the only two matching species that are present in La Selva Biological Station are *F. colubrinae* and *F. costaricana* (La Selva Florula Digital, http://sura.ots.ac.cr/florula4/). The remaining 18% of plant ITS sequences corresponded to various species of microscopic green algae (e.g., *Chlamydomonas* spp., *Parachlorella* spp., and *Trentepohlia* spp., among others). Most (i.e., 99.8%; σ = 0.17) of the ITS sequences from fruits matched to the same species as those found in feces.

### Comparison of fruit and fecal culture-independent fungal communities

**Fig. 1:**
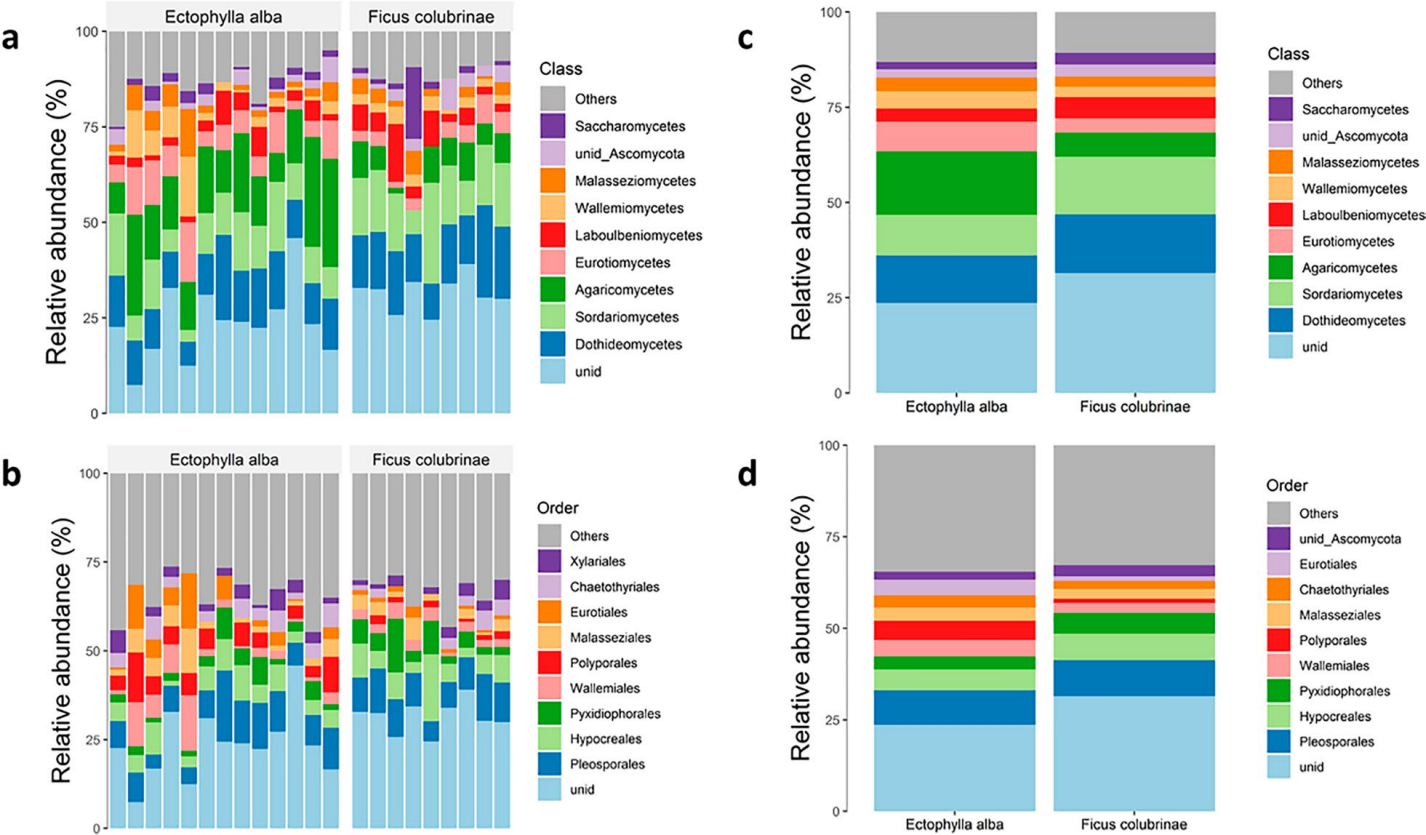
Barplots of fungal ASVs by class and order where panels (**a**) and (**b**) represent individual sample composition and panels (**c**) and (**d**) reflect overall group composition. Averaged taxa abundance per group is shown in c and d. Unidentified fungal taxa which could not be assigned to any other taxonomical category were aggregated as “unid” (light blue in all graphs); fungal taxa that could be assigned to *Ascomycota* but not further are aggregated as “unid_Ascomycota.” Only the 10 most abundant taxa are shown; uncommon taxa are combined in the category “others.”

The total number of fungal ASVs identified from metabarcoding of 9 pooled fruit samples (18 total fruits; sequencing failed for one of the fruit samples) and 13 bats was 460 and 1025, respectively (Additional file 1: Tables S1–S3). The phylum with the highest number of ASVs in both feces and fruits was *Ascomycota*, followed by *Basidiomycota* (Additional file 1: Fig. S1); a large percentage of ASVs (ca. 30%) did not match to any known fungal phylum. Figure 1 shows the most abundant (percent relative abundance of ASVs) classes and orders in the fecal and fruit samples. Excluding unidentified ASVs, fecal and fruit samples were dominated by the classes *Agaricomycetes* and *Dothideomycetes*, respectively (Fig. 1a, c, and Additional file 1: Fig. S1). The most frequent orders in feces (excluding unclassified ASVs) were *Pleosporales*, followed by *Polyporales, Chaetothyriales, Hypocreales*, and *Agaricales* (Fig. 1b, d, and Additional file 1: Fig. S1). Fruits were dominated by *Pleosporales*, followed by *Hypocreales, Chaetothyriales, Capnodiales*, and *Glomerellales*. Lastly, the genera with the greatest number of ASVs in the fecal samples were *Malassezia*, followed by *Wallemia, Aspergillus, Fusarium*, and *Pyxidiophora* (Additional file 1: Fig. S1). In contrast, *Malassezia, Pyxidiophora, Colletotrichum, Ochroconis,* and *Diaporthe* dominated the fruit mycobiota.

**Fig. 2:**
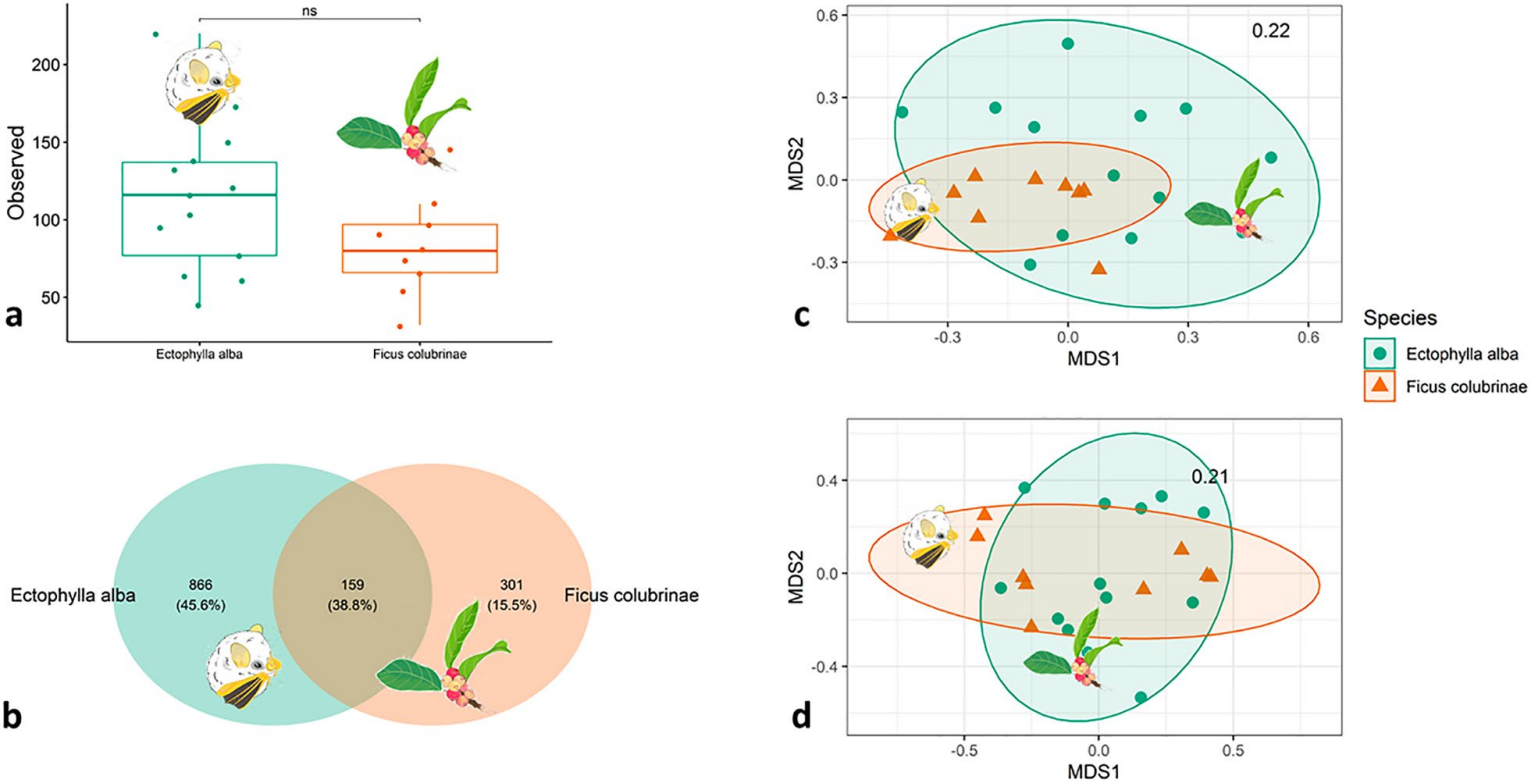
Alpha diversity (**a**), Venn diagram (**b**), and NMDS based on Jaccard distance for presence/absence data (**c**) and NMDS based on Bray–Curtis distance for normalized abundance data (**d**). **a**: *ns* non-significant difference. **c**, **d** the values within the plot represent the stress values, and ellipses the 95% confidence interval. Bat and fruit illustrations provided by Silvia Chaves Ramírez

While a larger number of fungal taxa was observed in bat feces than in fruit, alpha diversity did not differ significantly between the two communities (Fig. 2a) even when considering samples collected by tree (Additional file 1: Fig. S2a). However, fungal communities of fruits and feces were significantly different (presence/absence data: R = 0.07, F_1,20_ = 1.51, *P* < 0.001; normalized abundance data: R = 0.08, F_1,20_ = 1.84, *P* = 0.05) and there was no significant association between the fungal communities of bats and fruits collected from the same trees (Additional file 1: Figs. S2b–d). There were 866 unique fungal ASVs present in bat feces and 301 in fruits (Fig. 2b), resulting in overall distinct communities as visualized in the NMDS ordination (Fig. 2c, d); 159 ASVs overlapped. Both communities include *Cystofilobasidium*, *Fusarium*, *Geranomyces*, *Malassezia*, *Pyxidiophora*, and *Wallemia* (Additional file 1: Tables S1–S3).

**Fig. 3:**
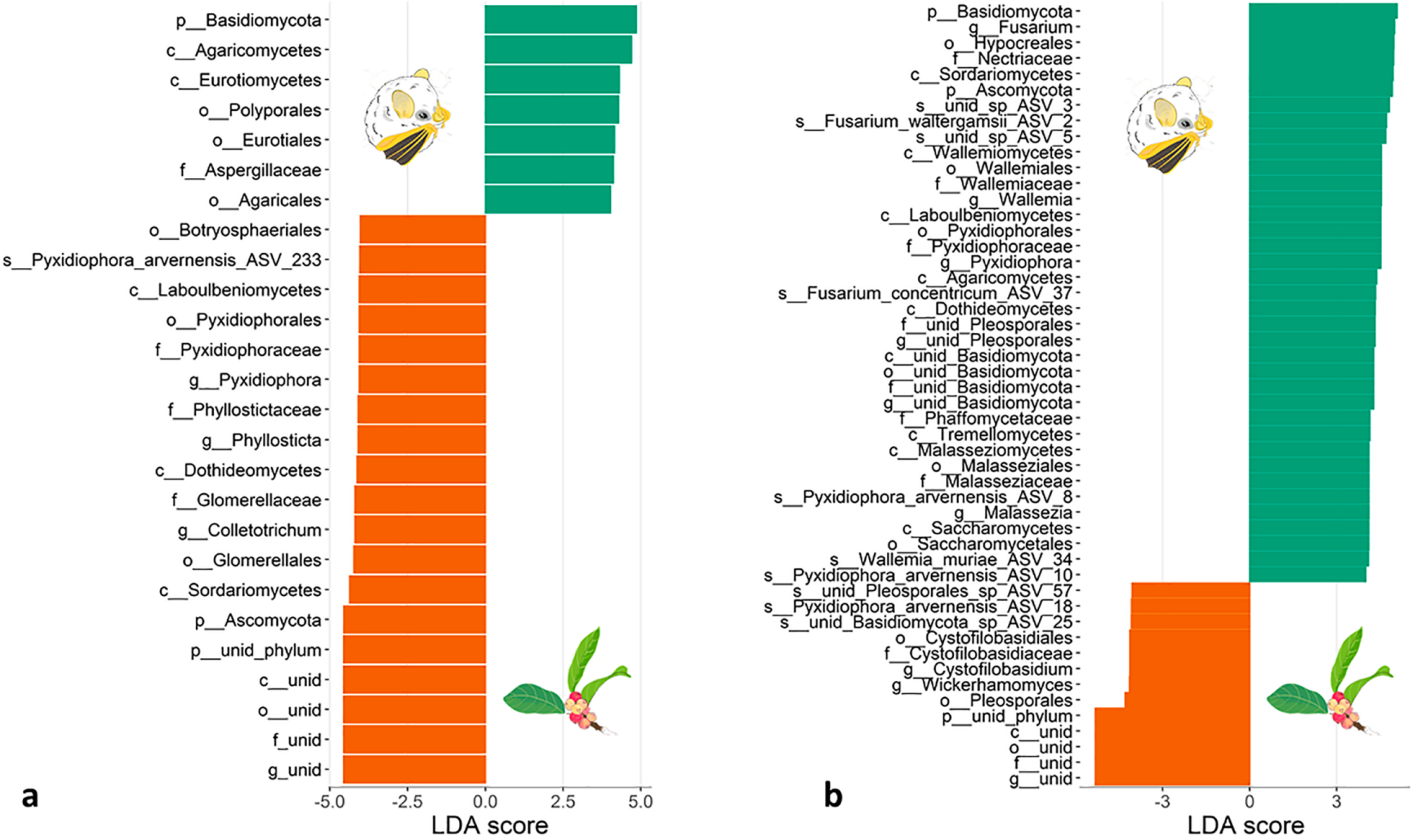
LDA score is the linear discriminant analysis score in LEfSe (**a**: results based on presence/absence data, **b**: results based on normalized abundance data). The letters represent taxonomic classifications: phylum (p), class (c), order (o), family (f), genus (g) and species (s). Unidentified fungal taxa that could be assigned to a specific taxonomic level are aggregated as “x_unid”. Bat and fruit illustrations provided by Silvia Chaves Ramírez

According to the LDA scores for presence/absence data (Fig. 3a), indicator taxa for bat feces belong to the phylum *Basidiomycota* (i.e., *Agaricomycetes: Polyporales* and *Agaricales*) and the ascomycetous class *Eurotiomycetes* (i.e., *Eurotiales, Aspergillaceae*). For fruits, there are several unidentified taxa which constitute the larger contributors to differentiating their fungal communities from those found in feces. Other indicator taxa in fruits belong in the phylum *Ascomycota* (i.e., *Dothideomycetes: Botryosphaeriales, Phyllostictaceae*, *Phyllosticta*; *Laboulbeniomycetes: Pyxidiophorales, Pyxidiophora*; and *Sordariomycetes: Glomerellales, Colletotrichum*). In contrast, when using normalized abundance (Fig. 3b), the analyses resulted in more and somewhat different indicator taxa in bat feces. For example, many unidentified *Basidiomycota* and *Ascomycota* groups, in addition to *Malasseziomycetes* (i.e., *Malasseziales, Malassezia*), *Wallemiomycetes* (i.e., *Wallemiales, Wallemia*), *Sordariomycetes* (i.e., *Hypocreales, Fusarium*), *Laboulbeniomycetes* (i.e., *Pyxidiophorales, Pyxidiophora*), and *Dothideomycetes* (i.e., *Pleosporales*), among others. In fruits, indicator taxa belong in *Tremellomycetes* (i.e., *Cystofilobasidiales, Cystofilobasidium*), *Saccharomycetes* (i.e., *Saccharomycetales*, *Wickerhamomyces*), and *Laboulbeniomycetes* (i.e., *Pyxidiophorales, Pyxidiophora*).

### Comparison of fruit and fecal culture-dependent fungal communities

Fungal colonies grew successfully from 4 fruits samples (11 isolates) and 10 fecal samples (30 isolates). The taxa present in fruit isolates included *Colletotrichum fruticola, C. siamense*, *Diaporthe* sp., *D*. cf. *hongkongensis*, *Fusarium concentricum*, and *Neopestalotiopsis saprophytica*; all matching ASVs that were also found in the culture-independent (metabarcoding) diversity analyses (Additional file 1: Table S3 and Additional file 1: Fig. S3). In fecal samples, the culture-dependent diversity was represented by *Fusarium concentricum, F. waltergamsii, Mucor irregularis, Neopestalotiopsis saprophytica*, and *Pseudopestalotiopsis simitheae*, also corresponding to ASVs obtained from the metabarcoding analyses. *Colletotrichum* spp. and *Diaporthe* spp. were only present in fruits in both culture-dependent and independent analyses. *Neopestalotiopsis saprophytica* was found in both fruits and feces. *Fusarium concentricum* dominated bat feces; however, some ASVs and cultures that matched this taxon were also observed in fruits. *Fusarium waltergamsii* grew only from fecal samples. However, metabarcoding data suggest that this taxon was also present in fruits. *Mucor irregularis* was only found in fecal samples in both culture-dependent and independent analyses.

### Analyses of putative ecological roles of the fungal taxa

**Fig. 4:**
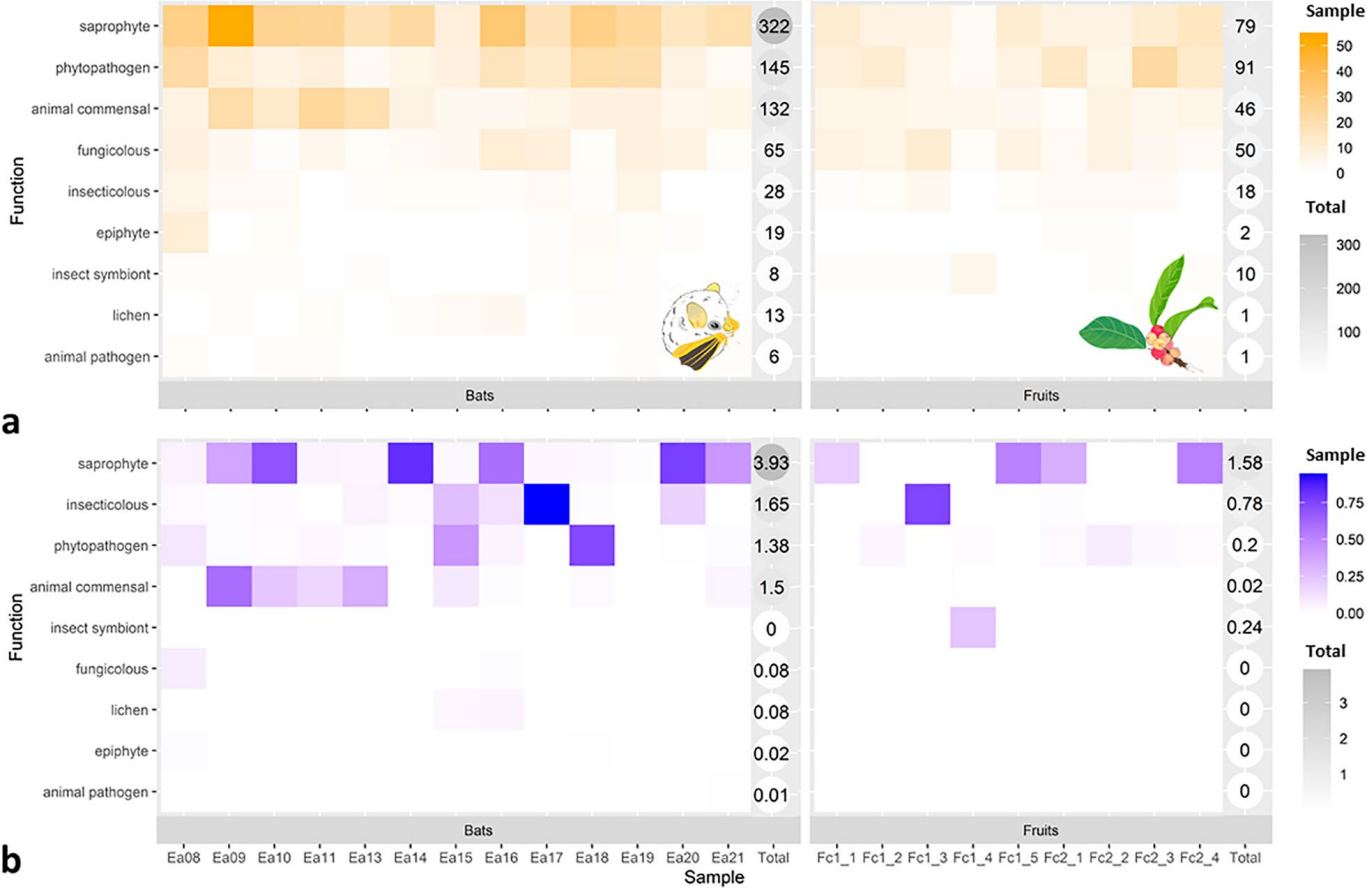
Heatmaps showing the relative contribution of taxa with a known function within each sample (columns) in bat feces and fruit communities based on presence/absence data (**a**) and normalized abundance (**b**). Sample abundance for (**a**) is based on the number of taxa found within each sample per function, whereas sample abundance for (**b**) is based on the sum of proportions of taxa for all taxa of a given function within samples. We also include a total column for each community, which shows the sum of all identified taxa within samples (**a**) or the sum of proportions (**b**) for a given function; grey shades provide an estimate of relative abundance. Bat and fruit illustrations provided by Silvia Chaves Ramírez

Based on results from FUNGuild annotation tool with modified assignments (see Methods section), in Fig. 4 we highlight the relative contribution of ASVs per sample according to putative ecological guild and based on presence/absence (Fig. 4a) and normalized abundance (Fig. 4b). Overall, excluding the unclassified/unidentified taxa, fecal samples were dominated by saprobes (e.g., *Agaricales*, *Polyporales,* and *Eurotiales*), phytopathogens (i.e., *Fusarium concentricum*), and animal (not insect) commensals (e.g., *Malasseziales* and *Wallemiales*), while fruit mycobiota was dominated by phytopathogens (e.g., *Botryosphaeriales* and *Glomerellales*), saprobes, and arthropod-associated taxa (i.e., *Pyxidiophora* and *Wickerhamomyces*). The LDA scores using presence/absence and normalized abundance (Fig. 3) showed that the indicator taxa in feces were mostly groups classified as saprobes and animal commensals. In contrast, the indicator taxa in fruits include phytopathogens and arthropod-associated fungi.

## Discussion

One of the hypotheses we posed in this study was that bats may disperse, through fecal deposition, the fungi that inhabit the fruits they consume. The combination of qualitative (presence/absence) and quantitative (normalized abundance) data suggest that approximately 38% of the original fruit mycobiota (i.e., species composition) remains in the fecal samples, even though the overall comparison of fruit and fecal fungal assemblages yielded marginally significant differences (Fig. 2). Through culture-dependent techniques we show that some of these fungal species originating from the fruit are still viable in feces, suggesting bats may be capable of dispersing fungi over long distances. Our characterization of the mycobiota through metabarcoding indicates the presence of fungal DNA (i.e., ITS nrDNA) in fruits that is similarly observed in bat feces, but we cannot conclude that all these species are still viable after passing through the digestive system. Our study found that out of the five culturable fungal species in *F. colubrinae* fruits, only two of those were recovered in cultures of bat feces. However, some of the ASVs identified by metabarcoding that were present in the fruit mycobiota but were not represented in cultured isolates did successfully grow from fecal samples. A noteworthy example is *Fusarium waltergamsii*, which was present in both fruits and feces, but was only cultured from feces, suggesting that passage through the digestive tract may help some fungi germinate and grow [30–32]. However, even though ITS nrDNA continues to be the most utilised option in fungal metabarcoding studies, caution should be placed in taxonomy assignments using this marker, as studies point to the limitations in species circumscription [40].

Many studies which compare both culturable and unculturable mycobiota from environmental samples have found similar trends where only a very small portion (< 5%) of the total fungal diversity is recovered in cultures [41–43]. This large difference is mainly due to competition (e.g., presence of low-abundant and slow-growing microorganisms that may be outcompeted by high-abundant and fast-growing species), obligate biotrophy (e.g., the fungus can only grow on a living host or partner), and substrate specificity (e.g., failure to grow on conventional media because of inappropriate conditions of pH, temperature, redox state, or availability of essential nutrients) [44–46]. Notwithstanding, our results and those of others indicate that several fungal taxa can grow after passing through the digestive tract of vertebrates [32, 47]. Additional RNA, transcriptomic, or proteomic analyses may help us better understand the viable mycobiota and the functions and interactions between microbial species [48–51]. Understanding which species can successfully grow after passage through animal guts will provide a clearer picture of the role of frugivores in the dispersal of fungal endophytes.

Comparisons of the mycobiota of fruits and bat feces provide preliminary evidence on how frugivores may be affecting plants exposed to microbial communities within feces, how surviving fungal taxa may affect frugivores, and how fungi may affect the interaction between fruits and frugivores. In our study, the differences in fungal species composition between the two communities (fruits vs. feces, Fig. 2) also reflected changes in functional diversity as estimated by ecological guild analyses (Fig. 4). Indicator taxa analyses (Fig. 3) revealed that the core fecal mycobiota is comprised of saprobe fungi, while that of fruits is constituted mostly of phytopathogens and arthropod-associated fungi. Therefore, *Ficus colubrinae* seeds dispersed by frugivores may benefit from a reduction in the number of potentially plant pathogenic taxa inherent to the fruit body. These interactions could increase seed survival which is often very low, primarily due to pathogenesis [52]. We can then infer that if *F. colubrinae* fruits are not consumed by frugivores and they just fall directly from the tree, potential plant pathogens contained in fruits will remain near the parent tree and may cause disease in seedlings. These results support the Janzen–Connell hypothesis [53, 54] or the Theory of pest pressure [55], which suggest that specialized natural enemies (i.e., plant pathogens) decrease survival of seedlings that are in high densities beneath the parent tree, thus giving locally rare species an advantage. An alternative scenario is that if fruit-eating bats defecate under or near *F. colubrinae* trees, or under or near their roosts (leaves of *Heliconia* spp.) [56], passage through the digestive tract may still result in decreased abundance of potential plant pathogens, which would reduce disease. Therefore, consumption of fruits by animals (i.e., bats) could not only benefit the plant by dispersing its seeds, but also by reducing the amount of pathogen inoculum and thus escape disease pressure [57].

A noteworthy and novel finding was *Pyxidiophora,* an obligate mycoparasitic fungal genus [58] found in both fruit and fecal mycobiota. The biology of this genus is poorly studied, but a few studies show that the sexual spores (ascospores) attach to phoretic mites of bark beetles [59]. The fungal spores are vectored by the insects, which then land on other fungi to become mycoparasitic [58]. As we found *Pyxidiophora* to be a core constituent of *Ficus colubrinae* fruits (Fig. 3), we hypothesize that this fungus is associated with the fig pollinating wasps (*Pegoscapus* spp., *Agaonidae, Chalcidoidea*) which were abundant inside the fruits we collected [60]. We also demonstrate that *Pyxidiophora* DNA is present in bat feces, suggesting that *Ectophylla alba* may also disperse these fungi. Previous studies report that *Pyxidiophora* is a common viable fungus parasitizing dung fungi [58]. Our study expands the list of potential arthropod hosts, suggesting that *Pyxidiophora* may not only be restricted to mites and beetles. Another fungus that may be associated with fig pollinating wasps is *Wickerhamomyces*, which has been reported from the midgut and gonads of several insects [61, 62].

While we still lack sufficient information to predict how certain fungal taxa found in the tissues of fruits may be affecting fruit-eating animals, some of the fungi we recorded in feces that were also present in fruits are considered common animal endosymbionts associated with healthy guts [63–65], such as *Malasseziales* and *Wallemiales*. In addition, some of the fungi that were found in feces are known to aid in digestion by improving the metabolizable energy of plant-based diets due to their ability to produce CAZymes and endo-beta(1,4)-xylanases (e.g., *Aspergillus* and related taxa, [66]) and plant cell-wall degrading enzymes (e.g., *Basidiomycota: Polyporales*, or *Ascomycota: Hypocreales*, *Fusarium* spp. [67]), in addition to the production and release of carotenoids, lipids, and coenzyme Q10 into the intestine (e.g., *Cystofilobasidium* and *Sporidiobolales*, [68–71]).

Finally, fungi may also affect the interaction between plants and frugivores. Fungi can produce secondary metabolites and volatile organic compounds (VOCs) which can affect fruit palatability and attraction [72–76]. For example, *Fusarium verticillioides* (related to *F. concentricum*, a species found in our fruit samples) and other *Fusarium* spp*.* have been found to produce VOCs that attract insects [77, 78]. By extension, we hypothesize that endophytic fungal communities may change the chemical composition of fruits and hence preferences in frugivores. While unquestionably relevant for understanding mutualistic networks, this topic remains completely unexplored.

This poorly studied interaction among fungal endophytes, fruits, and frugivores suggests there is a critical component of plant-animal ecological networks that urgently requires further scrutiny. This additional link complicates our understanding of mutualistic network dynamics and has implications for models developed thus far. For example, we often regard frugivores as somewhat equally responsible for promoting seed dispersal, yet differing foraging styles and physiological conditions among fruit-eating species can potentially affect dispersal distance and viability of propagules, fungal or otherwise [79, 80]. By affecting fruit palatability and overall plant fitness, the mycobiota may also be highly responsible for the success, or failure, of certain plant species, which ultimately modifies the composition of many ecological networks and interactions therein.

Study of the mycobiota has experienced a major increase in recent years, predominantly with the advent of genetic tools, and evidence is accumulating on the many roles that fungi play in natural ecosystems. Many of the studies conducted so far have shown a diverse fungal community in plants, yet surprisingly, very little is known about mycosymbionts in fruits (but see [38, 81–83]) despite the obvious role of fruits for plant fitness, and no studies to date have assessed how vertebrate consumption can affect fungal endophytes. Consequently, the role of endophytic fungi in mutualistic networks has been, until now, largely ignored. Our study shows that fungal endophytes are ubiquitous within fruits, and as such may be important components of plant-animal networks. Their ubiquity in plant tissues and the potential role that plant-eating organisms can play in dispersing the fungal propagules, suggest that further studies into mutualistic interactions should consider greater focus on endosymbionts.

## Conclusions

We conclude that fungal communities in *Ectophylla alba* feces and *Ficus colubrinae* fruits are fundamentally different, with about 38% of the ASVs shared between the two sample groups. As suggested by previous studies, we confirmed that a combination of qualitative (presence/absence) and quantitative (normalized abundance) data provides a more complete depiction of the fungal communities studied. We also show that there are several viable fungi and the presence of fruit ASVs in fecal samples, suggesting bats may be important dispersers for those fungi. The fruit mycobiota is dominated mostly by fungi with potential plant pathogenic activity, whereas fecal samples are dominated by saprobes. We also established, through metabarcoding, that *Ectophylla alba*’s main diet is based on *Ficus colubrinae* fruits. Our findings indicate that the role of frugivores in plant-animal mutualistic networks may extend beyond seed dispersal: they may also promote the dispersal of potentially beneficial microbial symbionts while simultaneously hindering those that can cause plant disease.

## Methods

### Study system

Fresh ripe fig fruits (*Ficus colubrinae*, *Moraceae*) and Honduran White Bat (*Ectophylla alba*, *Chiroptera: Phyllostomidae*) fecal samples were collected for fungal community analyses. It has been reported that *E. alba* feeds almost exclusively from *F. colubrinae* fruits [84]. *Ectophylla alba* is known only from Honduras, Nicaragua, Costa Rica, and western Panama [85]. In 2008, IUCN elevated this bat species to a near threatened Red List category [86]. Populations of this bat species have been declining due to urbanization and strong habitat (i.e., *Heliconia* leaves from intermediate secondary succession forests that are used for roosting) and diet (i.e., *F. colubrinae* fruits) preferences [86, 87]. *Ficus colubrinae* is an understory tree distributed from Mexico to Colombia, fruits throughout the year (La Selva Florula Digital, http://sura.ots.ac.cr/florula4/), and is a food source for many bats and birds [88].

### Fruit and fecal sample collection

Samples were collected in La Selva Biological Station (Sarapiquí, Heredia, Costa Rica). Two trees that were fruiting at the time of the fieldwork (February 2015) were chosen for fruit and fecal sample collection. We captured bats and collected fruits from the same trees as to increase the chances that the fecal samples came from the fruits consumed in that tree. Both sample types were subjected to gene-amplicon targeted sequencing (metabarcoding) and culture analyses. Each collection consisted of four adjacent ripe fruits (i.e., from the same cluster) placed into individual Ziploc bags. The clusters were picked from five randomly selected but reachable areas of each tree. Out of the four fruits per cluster, two were placed in 2-mL Eppendorf microtubes with silica gel and frozen at -20 °C for posterior DNA extraction. The remaining two were used for isolation into pure culture (see “Culturable fungi isolation and identification” section). Hereafter, trees are labeled as “Fc1” and “Fc2”, respectively, and each fruit sample from each tree as e.g., Fc1_1, Fc1_2, and so on. In total, we obtained 10 pooled fruit samples (representing 20 individual fruits) for metabarcoding and 20 fruits for culture analyses; 40 fruits for the entire study (2 trees × 5 clusters × 4 fruits = 40 fruits).

From trees Fc1 and Fc2, five and eight bats, respectively, were captured with mist nets (Ecotone, Poland) and immediately placed in sterilized cloth bags. To avoid cross-contamination, we cleaned our hands with an alcohol-based hand gel before releasing every bat from the net*.* When bats defecated, sterilized cotton swabs were used to obtain the fecal sample from the cloth bag. The approximate volume collected was a 4–5-mm-diam. pellet. Half of each sample was placed in 2-mL Eppendorf tubes, in Ziploc bags with silica gel, and then placed in a -20 °C freezer for subsequent metabarcoding analyses. The other half was placed in sterile Eppendorf tubes for later same-day culturing.

### Culturable fungi isolation and identification

Fruits were divided into five equal pieces and placed onto CMD + (BBL™ corn-meal-agar + 2% dextrose + antibiotic) 9-mm Petri dishes [89]. An antibiotic solution was added to the media to eliminate bacteria. From each fecal sample swab, five points of inoculation were made onto each CMD + Petri plate. The plates were incubated for several days (up to 2 weeks) at room temperature and the emerging colonies were subcultured to obtain pure isolates.

Genomic DNA from pure fungal cultures was extracted with PrepMan Ultra (Life Technologies, Waltham, MA, U.S.A.). The Internal Transcribed Spacer (ITS) and a region of the Large Subunit (28S) of the nuclear ribosomal DNA were amplified in one reaction, using the ITS5-forward (GGAAGTAAAAGTCGTAACAAGG) and LR5-reverse (TCCTGAGGGAAACTTCG) primers [90]. ITS is the official fungal barcode [40] and gives an approximate species identification. Polymerase chain reaction (PCR) conditions and protocols are described in previous publications [89]. PCR products were purified and sequenced at Macrogen U.S.A. Assembly of forward and reverse strands and sequence alignment were done in Geneious v 10.2.3 (https://www.geneious.com). BLASTn algorithm was performed in Geneious with retrieve from the UNITE v 2020 database. Best hits were compared and sequence identity of > 99% was used for taxonomy assignment.

### Metabarcoding of fruit and fecal samples

Genomic DNA from whole fruits and feces was extracted with the following protocol: fruits or feces were placed in the -20 °C freezer for at least one day and then, when ready to extract, placed into new tubes prefilled with 500 µm garnet beads and a 6 mm zirconium grinding satellite bead (OPS Diagnostics LLC, NJ, U.S.A.). For sample homogenization, a FastPrep® instrument (Zymo Research, Irvine, CA, U.S.A.) was used at maximum speed (6.5 m/s) for 1 min. 750 µl of Qiagen® Lysis Buffer AP1 and 6 ul of QiaGen® RNase-A were added to each tube and incubated overnight at 65 °C. Total DNA was extracted using the Qiagen® DNeasy Plant Mini Kit according to manufacturer’s instructions.

PCR amplicons of the ITS2 nrDNA region using fungal-specific primers fITS7-forward (GTGARTCATCGAATCTTTG) and ITS4-reverse (TCCTCCGCTTATTGATATGC) [91] were tagged and multiplexed for paired-end sequencing on the Illumina MiSeq 2 × 300 platform at MRDNA (http://mrdnalab.com, Shallowater, TX, U.S.A.). The same ITS2 primers can also amplify some plant DNA [91] and thus can be used to confirm the bat’s main diet. Three PCR stochastic replicates were pooled and purified using calibrated AMPure XP beads (Beckman Coulter Life Sciences, Indianapolis, IN, U.S.A.). PCR from a pure fungal culture of a *Trichoderma koningiopsis* and UltraPure™ DNase/RNase-Free Distilled Water (Thermo Fisher Scientific, Waltham, MA, U.S.A.) were used as positive and negative/blank controls, respectively, in quality control for the downstream bioinformatics. All raw sequencing data have been submitted to the NCBI Sequence Read Archive (SRA) database under the BioProject ID PRJNA759639.

### Metabarcoding bioinformatics and fungal species identification

Cutadapt v 2.3 [92] in Python v 3.7.10 was used to remove primers. Dada2 v 1.21.0 [93] in RStudio v 1.4.1717 was used for quality inspection and filtering, trimming, merging paired-ends, sample or Amplicon Sequence Variant (ASV) inference, and chimera removal. Forward and reverse sequences were filtered and trimmed where the quality score dropped to < 20 (i.e., forward trimmed at nucleotide position 270 and reverse at 210), and with a maximum number of expected errors (maxEE) set to 2. Sequences were clustered into ASVs [94] and then filtered for chimeras. To assign taxonomy, ASVs were subjected to similarity searches in the UNITE v 2020 curated and quality-checked database [95] using DECIPHER v 2.0 [96]. Each name was verified manually for nomenclatural accuracy either in Index Fungorum or Mycobank.

### Fungal diversity and community analyses

We analyzed the microbial communities found in fruits and bat feces using the package microeco v 0.6.5 [97] in R v 4.1.1. We transformed the ASV table to a matrix of presence/absence data (qualitative method) and estimated alpha (observed) and beta (Jaccard distance) diversity. Studies have suggested that using both quantitative and qualitative diversity measures will often be critical for understanding the factors that affect microbial diversity [98]. Therefore, to complement the results generated from presence/absence data, we also performed beta diversity analyses using abundance data (quantitative method) transformed to proportions [99]. This was accomplished by dividing the number of reads for each ASV in a sample (bat feces or fruit) by the total number of reads in that sample [99]. The distance matrix was constructed using the Bray–Curtis index. Alpha diversity (observed) was compared between the two communities using a t-test. Differences between the communities were determined based on ordination, using a non-metric multidimensional scaling (NMDS; [100]), and group distance, using a permutational multivariate analysis of variance (perMANOVA; [101]). Additionally, we identified which taxa might help us explain the differences between bat and fruit communities using the linear discriminant analysis (LDA) effect size (LEfSe) method [102]. We retained taxa with LDA scores > 4. In general, we placed more weight on the results from presence/absence data [100, 103, 104] over that from normalized abundance because we sought to determine whether the same fungal taxa in fruits were present in feces, and because of the intrinsic issues with misestimation of abundance in microbiome studies [99, 104–108].

### Estimation of abundance of fungal taxa according to their ecological roles

We explored if there was a trend in the relative abundance and presence/absence of ASVs with specific ecological roles in fruits and feces. A putative ecological or functional role was assigned by first parsing fungal community datasets by ecological guild using FUNGuild annotation tool [109] implemented in Python v 3.6 through the supercomputer Kabré (CNCA-CONARE, Costa Rica), and then manually checking, refining, and modifying the assignments (following the approach in [110]). Since many of the assignments provided by FUNGuild were ambiguous or incorrect, and other taxa had no assignments at all, we used the following modified putative roles: saprobe, plant pathogen, entomopathogen, animal (not arthropod) commensal, insect symbiont (not pathogen), mycotroph or fungicolous, lichen-forming, epiphyte, animal (not arthropod) pathogen. At the time we collected the fruits, there were no disease symptoms or necrotrophy. Therefore, all the inferred ecological guilds refer to a hypothesized cryptic role [111, 112].

With the data on ecological guilds, presence/absence, and normalized abundance for taxa, we then constructed two separate heatmaps. In these maps, we only included taxa for which more than 10 hits were recorded overall. For data on presence/absence we estimated abundance of a given function based on the number of ASVs with that function that were found in a specific sample (i.e., bat feces or fruits). The heatmap was created by plotting abundance of each function for each sample. For normalized abundance, the heatmap was created by plotting the sum of proportions (frequency) for all taxa of a given function within samples.

## Supplementary Information

**Additional file 1**. Supplementary Information (Tables and Figures).

## Data Availability

All code and raw data have been stored in the GitHub repository (https://github.com/morceglo/Fungal-communities-in-bats-and-fruits.git) and the NCBI Sequence Read Archive (SRA) database under the BioProject ID PRJNA759639.

## Abbreviations

ASV: Amplicon sequence variant
CMD: Corn-meal agar + dextrose
ITS: Internal transcribed spacer
LDA: Linear discriminant analysis
LEfSe: LDA effect size method
maxEE: Maximum number of expected errors
NMDS: Non-metric multidimensional scaling
PCR: Polymerase chain reaction
perMANOVA: Permutational multivariate analysis of variance
VOC: Volatile organic compound
