# Supplementary material for: Fungal communities in feces of the frugivorous bat Ectophylla alba and its highly specialized Ficus colubrinae diet
Source: Anim Microbiome. 2022 Mar 18;4:24. doi: 10.1186/s42523-022-00169-w (PMC8932179; doi:10.1186/s42523-022-00169-w)
Supplement: Supplementary file 1 — Additional file 1. Supplementary Information (Tables and Figures). [file 42523_2022_169_MOESM1_ESM.pdf]

Supplementary Figures

Figure S1. Number of ASVs per fungal group. For order and genus levels, only the top 20 (in number of ASVs) are included.

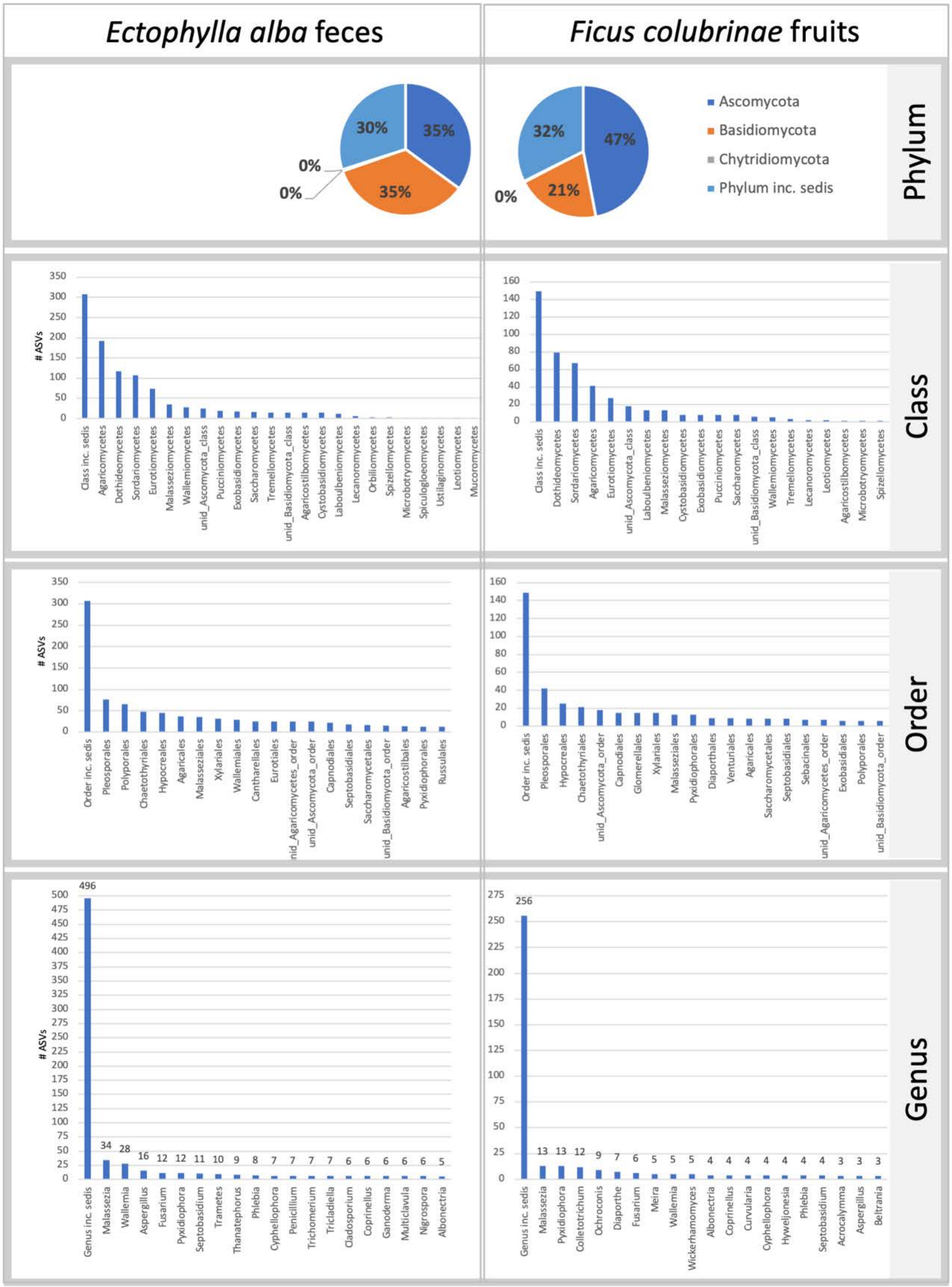

Figure S2. Alpha (a) and beta diversity (b), Venn diagram (c), and PCoA based on Jaccard distance (d). (a and b): ns = non-significant difference, * = $p < 0.05$, *** = $p < 0.001$, **** = $p < 0.0001$. (c): the percentages represent the sequence number percentages of ASVs in this fraction. (d): the ellipse represents the 95% confidence interval. Data are presented by community (bat feces: *Ectophylla alba*, and fruits: *Ficus colubrinae*) and by tree (Fc1: tree 1, Fc2: tree 2) where the fruits and bats were collected from.

.

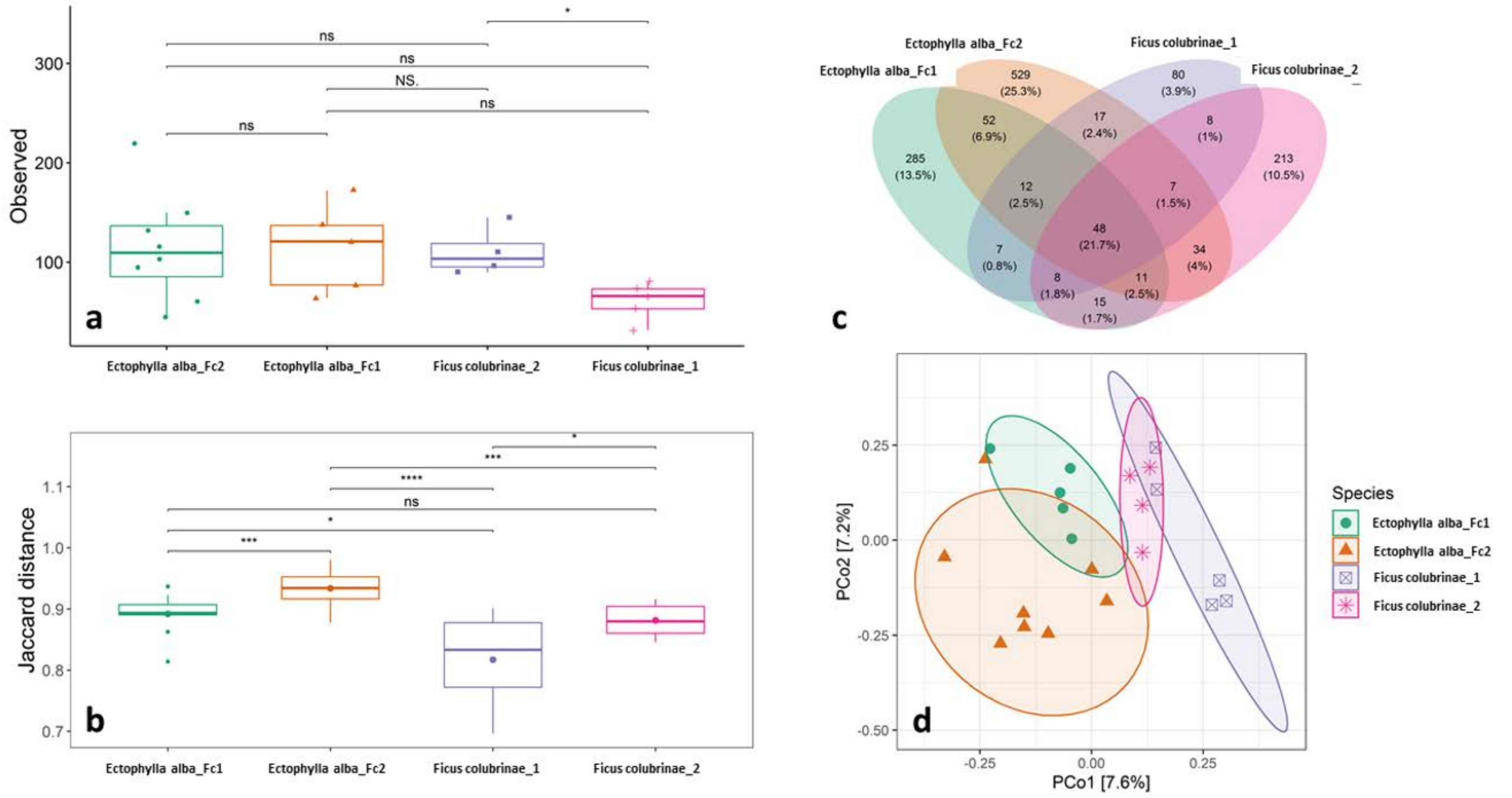

Figure S3. Position of the culturable fungi isolated from fecal and fruit samples within a cladogram that includes all sequences from metabarcoding. 1526 sequences were aligned with MAFFT. The cladogram was built with Neighbor-Joining and the HKY genetic distance model. The alignment and cladogram were done with Geneious Prime v 2021.1.1. The tree was visualized and edited in FigTree v 1.4.4. a) Cartoon cladogram with all sequences. b) Detail of *Mucor irregularis* clade. c) Detail of *Fusarium* spp. and related taxa. d) Detail of *Colletotrichum* spp., *Diaporthe* spp., and related taxa. e) Detail of *Neopestalotiopsis* spp., *Pseudopestalotiopsis* spp., and related taxa.

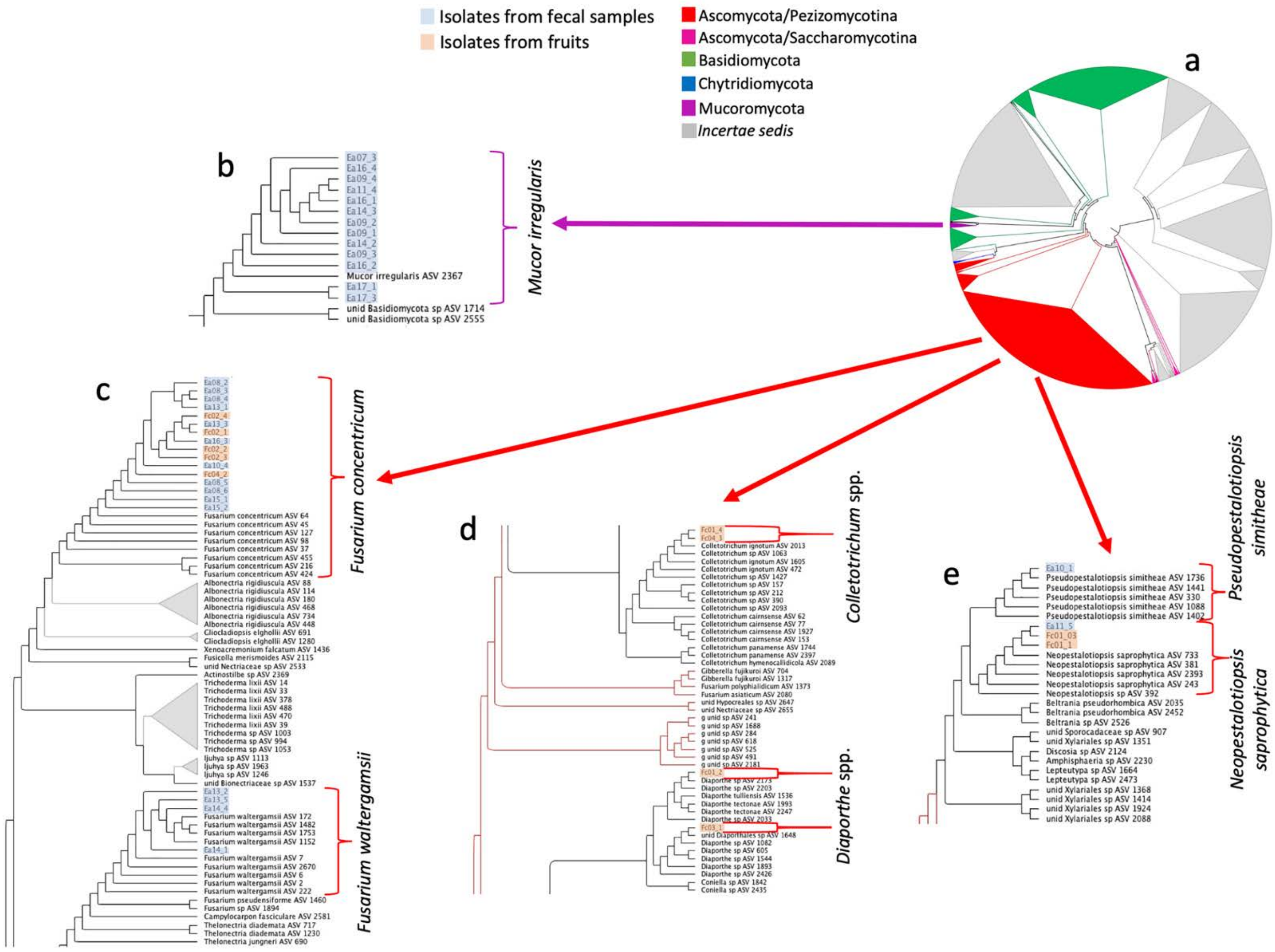

Supplementary Tables

Table S1. Abundance (number of sequences) data for the metabarcoding of the mycobiota of *Ectophylla alba* feces.

ASV	Ea08	Ea09	Ea10	Ea11	Ea13	Ea14	Ea15	Ea16	Ea17	Ea18	Ea19	Ea20	Ea21
ASV_10	6	23	0	0	0	363	787	1318	36692	114	155	463	0
ASV_100	0	5	0	0	87	0	0	0	16	0	0	0	0
ASV_1004	0	80	0	0	0	0	0	0	0	0	0	0	0
ASV_1005	0	80	0	0	0	0	0	0	0	0	0	0	0
ASV_1006	0	0	0	80	0	0	0	0	0	0	0	0	0
ASV_1007	0	0	0	0	0	0	0	43	0	0	0	0	0
ASV_1008	0	0	0	0	0	0	0	9	0	52	0	19	0
ASV_1009	0	0	0	0	0	0	0	0	0	0	0	0	0
ASV_101	0	0	0	24	0	0	0	0	0	0	0	0	0
ASV_1014	0	63	0	0	0	0	0	0	0	0	0	0	0
ASV_1015	0	0	0	0	0	0	0	0	9	0	0	0	0
ASV_1016	0	0	0	0	0	0	0	0	0	55	0	0	0
ASV_1017	0	0	0	0	0	0	0	0	0	0	79	0	0
ASV_1018	0	0	0	0	0	0	0	0	0	0	0	0	79
ASV_1021	78	0	0	0	0	0	0	0	0	0	0	0	0
ASV_1022	0	78	0	0	0	0	0	0	0	0	0	0	0
ASV_1023	0	0	78	0	0	0	0	0	0	0	0	0	0
ASV_1024	0	0	0	0	0	66	0	0	12	0	0	0	0
ASV_103	0	4	0	3719	4	0	0	0	10	0	10	0	0
ASV_1030	77	0	0	0	0	0	0	0	0	0	0	0	0
ASV_1031	77	0	0	0	0	0	0	0	0	0	0	0	0
ASV_1032	0	0	77	0	0	0	0	0	0	0	0	0	0
ASV_1033	0	0	0	0	0	0	0	0	0	77	0	0	0
ASV_1034	0	0	0	0	0	0	0	0	0	0	0	0	0
ASV_1035	0	0	0	0	0	0	0	0	0	0	0	0	0
ASV_1040	0	0	0	0	0	76	0	0	0	0	0	0	0
ASV_1041	0	0	0	0	0	0	0	0	0	76	0	0	0
ASV_1042	0	0	0	0	0	0	0	0	0	69	0	7	0
ASV_1045	0	75	0	0	0	0	0	0	0	0	0	0	0
ASV_1046	0	0	0	0	0	0	75	0	0	0	0	0	0
ASV_1047	0	0	0	0	0	0	0	44	0	0	0	31	0
ASV_105	0	0	0	3668	0	0	0	0	0	0	0	0	0
ASV_1054	52	0	0	0	0	22	0	0	0	0	0	0	0
ASV_1055	0	0	0	0	0	0	0	0	0	74	0	0	0
ASV_106	0	0	0	0	0	97	0	0	0	153	0	0	0
ASV_1062	0	0	0	0	0	0	0	73	0	0	0	0	0
ASV_1063	0	0	0	0	0	0	0	0	0	0	0	0	0
ASV_1064	0	0	0	0	0	0	0	0	0	0	0	0	0
ASV_1067	72	0	0	0	0	0	0	0	0	0	0	0	0
ASV_1068	0	72	0	0	0	0	0	0	0	0	0	0	0
ASV_1069	0	0	58	0	0	0	0	6	0	0	8	0	0
ASV_107	0	3433	3	0	3	0	0	0	31	0	0	0	0
ASV_1070	0	0	0	72	0	0	0	0	0	0	0	0	0
ASV_1071	0	0	0	0	0	0	0	0	0	0	4	0	0
ASV_1078	0	71	0	0	0	0	0	0	0	0	0	0	0
ASV_1079	0	71	0	0	0	0	0	0	0	0	0	0	0
ASV_108	25	2822	48	244	279	0	0	0	0	0	0	0	0
ASV_1080	0	0	0	71	0	0	0	0	0	0	0	0	0
ASV_1081	0	0	0	0	0	19	0	0	0	52	0	0	0
ASV_1082	0	0	0	0	0	0	0	0	0	0	0	0	0

ASV_109	0	0	0	0	0	0	0	0	0	0	3436	0	0
ASV_1092	0	0	0	0	0	70	0	0	0	0	0	0	0
ASV_1093	0	0	0	0	0	0	0	70	0	0	0	0	0
ASV_1094	0	0	0	0	0	0	0	0	0	70	0	0	0
ASV_1100	0	0	69	0	0	0	0	0	0	0	0	0	0
ASV_1101	0	0	0	69	0	0	0	0	0	0	0	0	0
ASV_1105	0	0	68	0	0	0	0	0	0	0	0	0	0
ASV_1106	0	0	0	0	0	0	0	0	0	0	0	0	0
ASV_1109	0	0	67	0	0	0	0	0	0	0	0	0	0
ASV_1110	0	0	0	0	0	67	0	0	0	0	0	0	0
ASV_1111	0	0	0	0	0	27	0	0	0	0	0	0	0
ASV_1112	0	0	0	0	0	0	0	0	0	67	0	0	0
ASV_1113	0	0	0	0	0	0	0	0	0	0	0	0	0
ASV_1120	66	0	0	0	0	0	0	0	0	0	0	0	0
ASV_1121	0	66	0	0	0	0	0	0	0	0	0	0	0
ASV_1122	0	66	0	0	0	0	0	0	0	0	0	0	0
ASV_1123	0	66	0	0	0	0	0	0	0	0	0	0	0
ASV_1124	0	0	66	0	0	0	0	0	0	0	0	0	0
ASV_1125	0	0	0	66	0	0	0	0	0	0	0	0	0
ASV_1126	0	0	0	0	0	0	0	66	0	0	0	0	0
ASV_1127	0	0	0	0	0	0	0	0	0	0	0	0	0
ASV_1128	0	0	0	0	0	0	0	0	0	0	0	0	0
ASV_1129	0	0	0	0	0	0	0	0	0	0	0	0	0
ASV_1130	0	0	0	0	0	0	0	0	0	0	0	0	0
ASV_1136	65	0	0	0	0	0	0	0	0	0	0	0	0
ASV_1137	0	65	0	0	0	0	0	0	0	0	0	0	0
ASV_1138	0	0	0	0	0	0	0	0	0	65	0	0	0
ASV_1139	0	0	0	0	0	0	0	0	0	0	0	0	0
ASV_114	0	0	0	17	12	0	0	0	0	18	0	0	0
ASV_1142	64	0	0	0	0	0	0	0	0	0	0	0	0
ASV_1143	0	0	0	0	0	0	64	0	0	0	0	0	0
ASV_1144	0	0	0	0	0	0	0	64	0	0	0	0	0
ASV_1145	0	0	0	0	0	0	0	0	0	0	0	0	0
ASV_115	3156	0	0	0	0	0	0	0	0	0	0	10	0
ASV_1151	0	0	0	0	0	0	0	63	0	0	0	0	0
ASV_1156	62	0	0	0	0	0	0	0	0	0	0	0	0
ASV_1157	0	0	0	62	0	0	0	0	0	0	0	0	0
ASV_1158	0	0	0	0	0	62	0	0	0	0	0	0	0
ASV_1159	0	0	0	0	0	0	0	62	0	0	0	0	0
ASV_1160	0	0	0	0	0	0	0	49	0	0	0	9	0
ASV_1161	0	0	0	0	0	0	0	0	0	0	62	0	0
ASV_1167	59	0	0	0	0	0	0	0	0	0	2	0	0
ASV_1168	0	61	0	0	0	0	0	0	0	0	0	0	0
ASV_1169	0	0	0	61	0	0	0	0	0	0	0	0	0
ASV_117	0	0	0	0	0	0	0	0	0	0	0	0	0
ASV_1170	0	0	0	61	0	0	0	0	0	0	0	0	0
ASV_1171	0	0	0	61	0	0	0	0	0	0	0	0	0
ASV_1172	0	0	0	0	0	0	0	0	0	0	0	0	0
ASV_1181	0	0	0	0	0	0	60	0	0	0	0	0	0
ASV_1184	0	0	0	0	0	0	0	59	0	0	0	0	0
ASV_1185	0	0	0	0	0	0	0	59	0	0	0	0	0
ASV_1186	0	0	0	0	0	0	0	59	0	0	0	0	0
ASV_1187	0	0	0	0	0	0	0	0	0	0	0	0	59
ASV_1188	0	0	0	0	0	0	0	0	0	0	0	0	0
ASV_1189	0	0	0	0	0	0	0	0	0	0	0	0	0
ASV_1194	0	0	0	58	0	0	0	0	0	0	0	0	0

ASV_1195	0	0	0	0	0	0	58	0	0	0	0	0	0
ASV_1196	0	0	0	0	0	0	0	0	0	0	58	0	0
ASV_1197	0	0	0	0	0	0	0	0	0	0	0	0	0
ASV_120	15	6	0	0	0	7	2	8	12	0	32	0	0
ASV_1204	57	0	0	0	0	0	0	0	0	0	0	0	0
ASV_1205	0	0	0	0	0	0	0	57	0	0	0	0	0
ASV_1206	0	0	0	0	0	0	0	57	0	0	0	0	0
ASV_1207	0	0	0	0	0	0	0	57	0	0	0	0	0
ASV_1208	0	0	0	0	0	0	0	0	7	50	0	0	0
ASV_1209	0	0	0	0	0	0	0	0	0	0	0	0	57
ASV_1210	0	0	0	0	0	0	0	0	0	0	0	0	57
ASV_1211	0	0	0	0	0	0	0	0	0	0	0	0	0
ASV_1219	0	0	56	0	0	0	0	0	0	0	0	0	0
ASV_1220	0	0	56	0	0	0	0	0	0	0	0	0	0
ASV_1221	0	0	56	0	0	0	0	0	0	0	0	0	0
ASV_1222	0	0	0	56	0	0	0	0	0	0	0	0	0
ASV_1223	0	0	0	0	0	56	0	0	0	0	0	0	0
ASV_1224	0	0	0	0	0	0	0	50	0	0	6	0	0
ASV_1225	0	0	0	0	0	0	0	0	0	56	0	0	0
ASV_1226	0	0	0	0	0	0	0	0	0	0	56	0	0
ASV_1227	0	0	0	0	0	0	0	0	0	0	0	0	56
ASV_1228	0	0	0	0	0	0	0	0	0	0	0	0	0
ASV_1229	0	0	0	0	0	0	0	0	0	0	0	0	0
ASV_123	0	0	0	234	0	0	0	0	0	0	0	0	0
ASV_1235	55	0	0	0	0	0	0	0	0	0	0	0	0
ASV_1236	0	0	0	0	0	0	0	0	0	55	0	0	0
ASV_1237	0	0	0	0	0	0	0	0	0	0	55	0	0
ASV_1238	0	0	0	0	0	0	0	0	0	0	0	0	0
ASV_1242	0	0	54	0	0	0	0	0	0	0	0	0	0
ASV_1243	0	0	0	0	0	0	0	44	10	0	0	0	0
ASV_1244	0	0	0	0	0	0	0	0	0	54	0	0	0
ASV_1245	0	0	0	0	0	0	0	0	0	54	0	0	0
ASV_1246	0	0	0	0	0	0	0	0	0	0	0	0	0
ASV_125	0	0	276	0	2417	0	0	0	0	0	38	0	0
ASV_1254	53	0	0	0	0	0	0	0	0	0	0	0	0
ASV_1255	0	0	53	0	0	0	0	0	0	0	0	0	0
ASV_1256	0	0	0	53	0	0	0	0	0	0	0	0	0
ASV_1257	0	0	0	0	0	0	53	0	0	0	0	0	0
ASV_1258	0	0	0	0	0	0	0	53	0	0	0	0	0
ASV_1259	0	0	0	0	0	0	0	53	0	0	0	0	0
ASV_126	2563	0	0	9	0	0	0	2	0	0	53	0	0
ASV_1267	52	0	0	0	0	0	0	0	0	0	0	0	0
ASV_1268	0	0	0	52	0	0	0	0	0	0	0	0	0
ASV_1269	0	0	0	3	0	0	0	0	0	49	0	0	0
ASV_127	0	0	0	0	0	0	0	0	0	2634	0	0	0
ASV_1270	0	0	0	0	0	0	0	0	0	0	52	0	0
ASV_1271	0	0	0	0	0	0	0	0	0	0	52	0	0
ASV_1272	0	0	0	0	0	0	0	0	0	0	52	0	0
ASV_1273	0	0	0	0	0	0	0	0	0	0	0	0	52
ASV_1274	0	0	0	0	0	0	0	0	0	0	0	0	0
ASV_1275	0	0	0	0	0	0	0	0	0	0	0	0	0
ASV_1282	0	51	0	0	0	0	0	0	0	0	0	0	0
ASV_1283	0	0	0	51	0	0	0	0	0	0	0	0	0
ASV_1284	0	0	0	0	51	0	0	0	0	0	0	0	0
ASV_1285	0	0	0	0	0	0	0	0	0	0	0	0	51
ASV_1289	0	0	0	0	0	0	0	0	0	0	0	0	0

ASV_1296	49	0	0	0	0	0	0	0	0	0	0	0	0
ASV_1297	49	0	0	0	0	0	0	0	0	0	0	0	0
ASV_1298	49	0	0	0	0	0	0	0	0	0	0	0	0
ASV_1299	0	0	0	49	0	0	0	0	0	0	0	0	0
ASV_13	0	0	6	0	37	6	0	0	0	0	55992	0	0
ASV_130	14	0	4	2375	0	0	0	0	0	0	0	0	0
ASV_1300	0	0	0	49	0	0	0	0	0	0	0	0	0
ASV_1301	0	0	0	49	0	0	0	0	0	0	0	0	0
ASV_1302	0	0	0	0	0	0	0	40	0	0	0	0	0
ASV_1303	0	0	0	0	0	0	0	0	0	49	0	0	0
ASV_1304	0	0	0	0	0	0	0	0	0	0	0	0	49
ASV_1312	0	0	0	0	0	0	0	48	0	0	0	0	0
ASV_1313	0	0	0	0	0	0	0	0	37	0	0	0	0
ASV_1314	0	0	0	0	0	0	0	0	0	0	0	0	0
ASV_1325	0	0	0	0	0	0	0	0	0	47	0	0	0
ASV_133	0	0	12	0	0	0	0	0	0	0	39	0	0
ASV_1331	0	0	0	0	0	0	0	46	0	0	0	0	0
ASV_1332	0	0	0	0	0	0	0	0	0	46	0	0	0
ASV_1333	0	0	0	0	0	0	0	0	0	0	46	0	0
ASV_1334	0	0	0	0	0	0	0	0	0	0	0	0	0
ASV_134	161	0	0	0	0	0	0	0	0	0	0	0	112
ASV_1341	0	0	0	45	0	0	0	0	0	0	0	0	0
ASV_1342	0	0	0	45	0	0	0	0	0	0	0	0	0
ASV_1343	0	0	0	0	0	45	0	0	0	0	0	0	0
ASV_1344	0	0	0	0	0	0	0	20	25	0	0	0	0
ASV_1345	0	0	0	0	0	0	0	0	0	0	45	0	0
ASV_1346	0	0	0	0	0	0	0	0	0	0	0	0	0
ASV_135	0	2180	0	0	0	0	0	0	0	0	0	0	0
ASV_1351	44	0	0	0	0	0	0	0	0	0	0	0	0
ASV_1352	0	0	0	0	0	0	0	44	0	0	0	0	0
ASV_1353	0	0	0	0	0	0	0	0	0	44	0	0	0
ASV_1354	0	0	0	0	0	0	0	0	0	44	0	0	0
ASV_1355	0	0	0	0	0	0	0	0	0	0	44	0	0
ASV_1356	0	0	0	0	0	0	0	0	0	0	0	0	0
ASV_1357	0	0	0	0	0	0	0	0	0	0	0	0	0
ASV_1358	0	0	0	0	0	0	0	0	0	0	0	0	0
ASV_1359	0	0	0	0	0	0	0	0	0	0	0	0	0
ASV_1366	0	0	43	0	0	0	0	0	0	0	0	0	0
ASV_1367	0	0	0	0	0	0	0	0	0	20	0	0	0
ASV_1368	0	0	0	0	0	0	0	0	0	0	0	0	0
ASV_1369	0	0	0	0	0	0	0	0	0	0	0	0	0
ASV_137	0	1808	0	0	0	0	337	0	36	0	0	0	0
ASV_1373	42	0	0	0	0	0	0	0	0	0	0	0	0
ASV_1374	0	0	0	0	0	0	0	42	0	0	0	0	0
ASV_1375	0	0	0	0	0	0	0	0	0	0	0	42	0
ASV_138	1778	0	313	0	0	0	0	0	0	0	21	0	0
ASV_139	0	0	0	2157	0	0	0	0	0	0	0	0	0
ASV_1390	0	0	0	41	0	0	0	0	0	0	0	0	0
ASV_1391	0	0	0	0	0	41	0	0	0	0	0	0	0
ASV_1392	0	0	0	0	0	0	0	0	0	0	0	0	0
ASV_140	0	0	0	1980	0	0	0	7	0	0	74	0	0
ASV_1400	40	0	0	0	0	0	0	0	0	0	0	0	0
ASV_1401	40	0	0	0	0	0	0	0	0	0	0	0	0
ASV_1402	40	0	0	0	0	0	0	0	0	0	0	0	0
ASV_1403	0	0	0	0	0	40	0	0	0	0	0	0	0
ASV_1404	0	0	0	0	0	0	0	40	0	0	0	0	0

ASV_1405	0	0	0	0	0	0	0	0	0	37	0	0	0
ASV_1412	0	0	0	0	0	0	0	39	0	0	0	0	0
ASV_1413	0	0	0	0	0	0	0	0	0	0	39	0	0
ASV_1414	0	0	0	0	0	0	0	0	0	0	21	0	0
ASV_1415	0	0	0	0	0	0	0	0	0	0	0	0	0
ASV_1422	0	10	0	0	28	0	0	0	0	0	0	0	0
ASV_1423	0	0	38	0	0	0	0	0	0	0	0	0	0
ASV_1424	0	0	0	0	0	0	0	38	0	0	0	0	0
ASV_1425	0	0	0	0	0	0	0	38	0	0	0	0	0
ASV_1426	0	0	0	0	0	0	0	0	0	0	0	0	0
ASV_1427	0	0	0	0	0	0	0	0	0	0	0	0	0
ASV_1428	0	0	0	0	0	0	0	0	0	0	0	0	0
ASV_1436	37	0	0	0	0	0	0	0	0	0	0	0	0
ASV_1437	0	0	37	0	0	0	0	0	0	0	0	0	0
ASV_1438	0	0	37	0	0	0	0	0	0	0	0	0	0
ASV_1439	0	0	2	0	0	0	0	0	0	0	0	0	35
ASV_1440	0	0	0	0	0	37	0	0	0	0	0	0	0
ASV_1441	0	0	0	0	0	0	37	0	0	0	0	0	0
ASV_1442	0	0	0	0	0	0	0	0	0	0	37	0	0
ASV_145	1876	0	0	0	0	0	0	0	0	0	49	0	0
ASV_1459	0	0	36	0	0	0	0	0	0	0	0	0	0
ASV_1460	0	0	0	0	0	0	0	0	0	36	0	0	0
ASV_1461	0	0	0	0	0	0	0	0	0	36	0	0	0
ASV_1462	0	0	0	0	0	0	0	0	0	36	0	0	0
ASV_1463	0	0	0	0	0	0	0	0	0	0	0	0	0
ASV_1464	0	0	0	0	0	0	0	0	0	0	0	0	0
ASV_147	480	0	0	0	0	0	0	0	0	0	0	0	0
ASV_1472	35	0	0	0	0	0	0	0	0	0	0	0	0
ASV_1473	0	0	0	35	0	0	0	0	0	0	0	0	0
ASV_1474	0	0	0	0	0	0	0	11	0	0	0	24	0
ASV_1475	0	0	0	0	0	0	0	0	0	0	0	35	0
ASV_1483	34	0	0	0	0	0	0	0	0	0	0	0	0
ASV_1484	0	0	0	0	0	34	0	0	0	0	0	0	0
ASV_1485	0	0	0	0	0	0	0	0	34	0	0	0	0
ASV_1486	0	0	0	0	0	0	0	0	0	26	0	0	0
ASV_1487	0	0	0	0	0	0	0	0	0	0	0	34	0
ASV_1488	0	0	0	0	0	0	0	0	0	0	0	0	34
ASV_1489	0	0	0	0	0	0	0	0	0	0	0	0	34
ASV_149	0	1752	0	0	0	0	0	0	0	0	0	0	0
ASV_1490	0	0	0	0	0	0	0	0	0	0	0	0	0
ASV_150	0	1729	0	0	0	0	0	0	16	0	0	0	0
ASV_1500	0	0	0	0	0	0	33	0	0	0	0	0	0
ASV_1501	0	0	0	0	0	0	0	0	0	33	0	0	0
ASV_1502	0	0	0	0	0	0	0	0	0	0	0	0	0
ASV_1503	0	0	0	0	0	0	0	0	0	0	0	0	0
ASV_1504	0	0	0	0	0	0	0	0	0	0	0	0	0
ASV_151	0	584	386	0	787	0	0	0	0	0	0	0	0
ASV_1515	0	0	0	32	0	0	0	0	0	0	0	0	0
ASV_1516	0	0	0	0	0	0	0	0	0	32	0	0	0
ASV_1517	0	0	0	0	0	0	0	0	0	0	32	0	0
ASV_1518	0	0	0	0	0	0	0	0	0	0	32	0	0
ASV_1519	0	0	0	0	0	0	0	0	0	0	32	0	0
ASV_152	0	0	0	0	0	0	0	0	0	0	0	0	0
ASV_1520	0	0	0	0	0	0	0	0	0	0	0	0	32
ASV_1530	0	31	0	0	0	0	0	0	0	0	0	0	0
ASV_1531	0	0	0	0	0	31	0	0	0	0	0	0	0

ASV_1532	0	0	0	0	0	24	0	0	7	0	0	0	0
ASV_1533	0	0	0	0	0	0	0	17	0	0	0	14	0
ASV_1534	0	0	0	0	0	0	0	7	7	0	17	0	0
ASV_1535	0	0	0	0	0	0	0	0	17	0	0	0	0
ASV_1536	0	0	0	0	0	0	0	0	0	0	0	0	0
ASV_1537	0	0	0	0	0	0	0	0	0	0	0	0	0
ASV_154	0	5	0	11	0	0	0	1667	0	0	9	0	0
ASV_1549	30	0	0	0	0	0	0	0	0	0	0	0	0
ASV_1550	30	0	0	0	0	0	0	0	0	0	0	0	0
ASV_1551	4	0	0	0	0	0	0	0	0	0	0	0	0
ASV_1552	0	30	0	0	0	0	0	0	0	0	0	0	0
ASV_1553	0	0	30	0	0	0	0	0	0	0	0	0	0
ASV_1554	0	0	0	0	0	0	30	0	0	0	0	0	0
ASV_1555	0	0	0	0	0	0	0	30	0	0	0	0	0
ASV_1556	0	0	0	0	0	0	0	0	0	12	6	0	0
ASV_1557	0	0	0	0	0	0	0	0	0	0	30	0	0
ASV_1558	0	0	0	0	0	0	0	0	0	0	30	0	0
ASV_1559	0	0	0	0	0	0	0	0	0	0	30	0	0
ASV_1560	0	0	0	0	0	0	0	0	0	0	18	5	0
ASV_1561	0	0	0	0	0	0	0	0	0	0	0	0	0
ASV_1562	0	0	0	0	0	0	0	0	0	0	0	0	0
ASV_1563	0	0	0	0	0	0	0	0	0	0	0	0	0
ASV_157	5	0	0	0	0	0	0	0	8	0	10	0	0
ASV_1578	29	0	0	0	0	0	0	0	0	0	0	0	0
ASV_1579	19	0	0	0	0	0	0	10	0	0	0	0	0
ASV_1580	0	0	29	0	0	0	0	0	0	0	0	0	0
ASV_1581	0	0	0	29	0	0	0	0	0	0	0	0	0
ASV_1582	0	0	0	7	0	0	0	0	0	0	0	0	0
ASV_1583	0	0	0	0	0	0	29	0	0	0	0	0	0
ASV_1584	0	0	0	0	0	0	0	29	0	0	0	0	0
ASV_1585	0	0	0	0	0	0	0	0	29	0	0	0	0
ASV_1586	0	0	0	0	0	0	0	0	0	29	0	0	0
ASV_1587	0	0	0	0	0	0	0	0	0	0	29	0	0
ASV_1588	0	0	0	0	0	0	0	0	0	0	0	0	29
ASV_159	0	1529	0	0	0	0	0	0	0	0	0	0	0
ASV_1602	28	0	0	0	0	0	0	0	0	0	0	0	0
ASV_1603	28	0	0	0	0	0	0	0	0	0	0	0	0
ASV_1604	28	0	0	0	0	0	0	0	0	0	0	0	0
ASV_1605	0	0	28	0	0	0	0	0	0	0	0	0	0
ASV_1606	0	0	0	28	0	0	0	0	0	0	0	0	0
ASV_1607	0	0	0	0	0	0	0	28	0	0	0	0	0
ASV_1608	0	0	0	0	0	0	0	0	21	0	0	0	0
ASV_1609	0	0	0	0	0	0	0	0	0	28	0	0	0
ASV_161	0	0	0	0	0	0	0	0	0	0	0	0	0
ASV_1610	0	0	0	0	0	0	0	0	0	28	0	0	0
ASV_1611	0	0	0	0	0	0	0	0	0	0	28	0	0
ASV_1612	0	0	0	0	0	0	0	0	0	0	0	28	0
ASV_1613	0	0	0	0	0	0	0	0	0	0	0	0	0
ASV_1614	0	0	0	0	0	0	0	0	0	0	0	0	0
ASV_162	0	0	0	0	0	0	0	0	0	0	0	0	1512
ASV_1626	0	27	0	0	0	0	0	0	0	0	0	0	0
ASV_1627	0	0	0	0	0	27	0	0	0	0	0	0	0
ASV_1628	0	0	0	0	0	0	0	27	0	0	0	0	0
ASV_1629	0	0	0	0	0	0	0	0	0	27	0	0	0
ASV_163	17	0	0	0	0	64	20	347	135	77	23	0	0
ASV_1630	0	0	0	0	0	0	0	0	0	0	27	0	0

ASV_1636	0	0	0	0	0	26	0	0	0	0	0	0	0
ASV_1637	0	0	0	0	0	0	0	26	0	0	0	0	0
ASV_1638	0	0	0	0	0	0	0	26	0	0	0	0	0
ASV_1639	0	0	0	0	0	0	0	0	26	0	0	0	0
ASV_1640	0	0	0	0	0	0	0	0	0	26	0	0	0
ASV_1641	0	0	0	0	0	0	0	0	0	26	0	0	0
ASV_1642	0	0	0	0	0	0	0	0	0	26	0	0	0
ASV_1643	0	0	0	0	0	0	0	0	0	0	26	0	0
ASV_1644	0	0	0	0	0	0	0	0	0	0	26	0	0
ASV_1645	0	0	0	0	0	0	0	0	0	0	26	0	0
ASV_1646	0	0	0	0	0	0	0	0	0	0	0	0	0
ASV_1647	0	0	0	0	0	0	0	0	0	0	0	0	0
ASV_1648	0	0	0	0	0	0	0	0	0	0	0	0	0
ASV_1649	0	0	0	0	0	0	0	0	0	0	0	0	0
ASV_166	0	228	0	0	1216	0	0	0	0	0	0	0	0
ASV_1661	25	0	0	0	0	0	0	0	0	0	0	0	0
ASV_1662	25	0	0	0	0	0	0	0	0	0	0	0	0
ASV_1663	0	0	25	0	0	0	0	0	0	0	0	0	0
ASV_1664	0	0	0	0	0	0	0	0	0	25	0	0	0
ASV_1665	0	0	0	0	0	0	0	0	0	25	0	0	0
ASV_1666	0	0	0	0	0	0	0	0	0	0	0	0	25
ASV_1667	0	0	0	0	0	0	0	0	0	0	0	0	0
ASV_1668	0	0	0	0	0	0	0	0	0	0	0	0	0
ASV_1669	0	0	0	0	0	0	0	0	0	0	0	0	0
ASV_1670	0	0	0	0	0	0	0	0	0	0	0	0	0
ASV_1671	0	0	0	0	0	0	0	0	0	0	0	0	0
ASV_168	1351	0	0	0	0	0	0	0	19	0	0	0	10
ASV_1684	24	0	0	0	0	0	0	0	0	0	0	0	0
ASV_1685	0	0	24	0	0	0	0	0	0	0	0	0	0
ASV_1686	0	0	0	0	0	0	0	24	0	0	0	0	0
ASV_1687	0	0	0	0	0	0	0	0	0	0	24	0	0
ASV_1688	0	0	0	0	0	0	0	0	0	0	0	24	0
ASV_1689	0	0	0	0	0	0	0	0	0	0	0	0	0
ASV_1690	0	0	0	0	0	0	0	0	0	0	0	0	0
ASV_17	0	0	0	524	0	54	0	0	0	0	45782	0	0
ASV_170	0	1359	0	0	0	0	0	0	0	0	0	0	0
ASV_1703	23	0	0	0	0	0	0	0	0	0	0	0	0
ASV_1704	23	0	0	0	0	0	0	0	0	0	0	0	0
ASV_1705	18	0	0	0	0	0	0	0	0	0	0	5	0
ASV_1706	0	23	0	0	0	0	0	0	0	0	0	0	0
ASV_1707	0	0	18	0	0	0	0	0	0	0	0	5	0
ASV_1708	0	0	0	0	0	23	0	0	0	0	0	0	0
ASV_1709	0	0	0	0	0	23	0	0	0	0	0	0	0
ASV_1710	0	0	0	0	0	23	0	0	0	0	0	0	0
ASV_1711	0	0	0	0	0	0	0	0	23	0	0	0	0
ASV_1712	0	0	0	0	0	0	0	0	0	23	0	0	0
ASV_1713	0	0	0	0	0	0	0	0	0	0	0	0	23
ASV_1714	0	0	0	0	0	0	0	0	0	0	0	0	23
ASV_1715	0	0	0	0	0	0	0	0	0	0	0	0	0
ASV_1731	22	0	0	0	0	0	0	0	0	0	0	0	0
ASV_1732	0	0	0	22	0	0	0	0	0	0	0	0	0
ASV_1733	0	0	0	0	0	0	0	22	0	0	0	0	0
ASV_1734	0	0	0	0	0	0	0	0	0	22	0	0	0
ASV_1735	0	0	0	0	0	0	0	0	0	22	0	0	0
ASV_1736	0	0	0	0	0	0	0	0	0	22	0	0	0
ASV_1737	0	0	0	0	0	0	0	0	0	22	0	0	0

ASV_1738	0	0	0	0	0	0	0	0	0	22	0	0	0
ASV_1739	0	0	0	0	0	0	0	0	0	0	22	0	0
ASV_174	0	0	0	0	0	37	0	1307	0	0	0	0	0
ASV_1740	0	0	0	0	0	0	0	0	0	0	16	0	0
ASV_1741	0	0	0	0	0	0	0	0	0	0	0	22	0
ASV_1742	0	0	0	0	0	0	0	0	0	0	0	0	22
ASV_1743	0	0	0	0	0	0	0	0	0	0	0	0	0
ASV_1744	0	0	0	0	0	0	0	0	0	0	0	0	0
ASV_1745	0	0	0	0	0	0	0	0	0	0	0	0	0
ASV_175	7	0	0	1255	0	0	0	0	0	0	78	0	0
ASV_1758	11	0	10	0	0	0	0	0	0	0	0	0	0
ASV_1759	0	12	0	0	0	0	0	0	0	9	0	0	0
ASV_1760	0	0	0	21	0	0	0	0	0	0	0	0	0
ASV_1761	0	0	0	0	0	21	0	0	0	0	0	0	0
ASV_1762	0	0	0	0	0	0	0	21	0	0	0	0	0
ASV_1763	0	0	0	0	0	0	0	0	21	0	0	0	0
ASV_1764	0	0	0	0	0	0	0	0	0	21	0	0	0
ASV_1765	0	0	0	0	0	0	0	0	0	0	21	0	0
ASV_1766	0	0	0	0	0	0	0	0	0	0	0	0	21
ASV_1767	0	0	0	0	0	0	0	0	0	0	0	0	0
ASV_1768	0	0	0	0	0	0	0	0	0	0	0	0	0
ASV_1779	0	0	0	0	0	20	0	0	0	0	0	0	0
ASV_1780	0	0	0	0	0	20	0	0	0	0	0	0	0
ASV_1781	0	0	0	0	0	0	0	20	0	0	0	0	0
ASV_1782	0	0	0	0	0	0	0	20	0	0	0	0	0
ASV_1783	0	0	0	0	0	0	0	20	0	0	0	0	0
ASV_1784	0	0	0	0	0	0	0	20	0	0	0	0	0
ASV_1785	0	0	0	0	0	0	0	16	0	0	0	0	0
ASV_1786	0	0	0	0	0	0	0	0	0	20	0	0	0
ASV_1787	0	0	0	0	0	0	0	0	0	0	20	0	0
ASV_1788	0	0	0	0	0	0	0	0	0	0	0	20	0
ASV_1789	0	0	0	0	0	0	0	0	0	0	0	0	0
ASV_179	0	7	0	9	0	66	0	0	0	0	16	555	603
ASV_1790	0	0	0	0	0	0	0	0	0	0	0	0	0
ASV_1791	0	0	0	0	0	0	0	0	0	0	0	0	0
ASV_18	17	0	0	3	0	0	1004	103	44357	0	84	80	0
ASV_1814	19	0	0	0	0	0	0	0	0	0	0	0	0
ASV_1815	0	0	19	0	0	0	0	0	0	0	0	0	0
ASV_1816	0	0	19	0	0	0	0	0	0	0	0	0	0
ASV_1817	0	0	0	0	0	0	0	19	0	0	0	0	0
ASV_1818	0	0	0	0	0	0	0	0	0	19	0	0	0
ASV_1819	0	0	0	0	0	0	0	0	0	0	19	0	0
ASV_182	0	0	0	0	0	0	0	770	0	410	16	0	0
ASV_1820	0	0	0	0	0	0	0	0	0	0	0	19	0
ASV_1821	0	0	0	0	0	0	0	0	0	0	0	0	0
ASV_1822	0	0	0	0	0	0	0	0	0	0	0	0	0
ASV_1823	0	0	0	0	0	0	0	0	0	0	0	0	0
ASV_183	0	0	0	0	0	0	0	0	0	0	0	0	0
ASV_1839	18	0	0	0	0	0	0	0	0	0	0	0	0
ASV_1840	0	0	18	0	0	0	0	0	0	0	0	0	0
ASV_1841	0	0	0	0	0	0	0	0	18	0	0	0	0
ASV_1842	0	0	0	0	0	0	0	0	0	18	0	0	0
ASV_1843	0	0	0	0	0	0	0	0	0	18	0	0	0
ASV_1844	0	0	0	0	0	0	0	0	0	0	18	0	0
ASV_1845	0	0	0	0	0	0	0	0	0	0	18	0	0
ASV_1846	0	0	0	0	0	0	0	0	0	0	18	0	0

ASV_1847	0	0	0	0	0	0	0	0	0	0	0	18	0
ASV_1848	0	0	0	0	0	0	0	0	0	0	0	18	0
ASV_1849	0	0	0	0	0	0	0	0	0	0	0	0	0
ASV_1850	0	0	0	0	0	0	0	0	0	0	0	0	0
ASV_186	0	0	0	0	0	0	0	0	0	0	0	0	0
ASV_1862	17	0	0	0	0	0	0	0	0	0	0	0	0
ASV_1863	17	0	0	0	0	0	0	0	0	0	0	0	0
ASV_1864	17	0	0	0	0	0	0	0	0	0	0	0	0
ASV_1865	0	17	0	0	0	0	0	0	0	0	0	0	0
ASV_1866	0	0	0	0	0	0	0	2	0	0	0	0	0
ASV_1867	0	0	0	0	0	0	0	0	0	17	0	0	0
ASV_1868	0	0	0	0	0	0	0	0	0	0	0	0	0
ASV_1869	0	0	0	0	0	0	0	0	0	0	0	0	0
ASV_1884	16	0	0	0	0	0	0	0	0	0	0	0	0
ASV_1885	0	0	0	0	0	16	0	0	0	0	0	0	0
ASV_1886	0	0	0	0	0	16	0	0	0	0	0	0	0
ASV_1887	0	0	0	0	0	16	0	0	0	0	0	0	0
ASV_1888	0	0	0	0	0	0	16	0	0	0	0	0	0
ASV_1889	0	0	0	0	0	0	0	16	0	0	0	0	0
ASV_1890	0	0	0	0	0	0	0	16	0	0	0	0	0
ASV_1891	0	0	0	0	0	0	0	0	0	0	16	0	0
ASV_1892	0	0	0	0	0	0	0	0	0	0	0	0	16
ASV_1893	0	0	0	0	0	0	0	0	0	0	0	0	0
ASV_1894	0	0	0	0	0	0	0	0	0	0	0	0	0
ASV_1895	0	0	0	0	0	0	0	0	0	0	0	0	0
ASV_19	0	0	0	0	0	0	0	0	0	0	43280	0	0
ASV_190	0	0	0	0	0	0	0	0	0	0	0	0	0
ASV_1913	8	0	0	0	0	0	0	0	0	0	0	0	0
ASV_1914	0	0	0	0	0	15	0	0	0	0	0	0	0
ASV_1915	0	0	0	0	0	15	0	0	0	0	0	0	0
ASV_1916	0	0	0	0	0	15	0	0	0	0	0	0	0
ASV_1917	0	0	0	0	0	0	0	15	0	0	0	0	0
ASV_1918	0	0	0	0	0	0	0	0	15	0	0	0	0
ASV_1919	0	0	0	0	0	0	0	0	15	0	0	0	0
ASV_192	0	0	0	372	0	0	0	0	0	0	0	0	0
ASV_1920	0	0	0	0	0	0	0	0	15	0	0	0	0
ASV_1921	0	0	0	0	0	0	0	0	0	0	15	0	0
ASV_1922	0	0	0	0	0	0	0	0	0	0	15	0	0
ASV_1923	0	0	0	0	0	0	0	0	0	0	15	0	0
ASV_1924	0	0	0	0	0	0	0	0	0	0	15	0	0
ASV_1925	0	0	0	0	0	0	0	0	0	0	15	0	0
ASV_1926	0	0	0	0	0	0	0	0	0	0	0	15	0
ASV_1927	0	0	0	0	0	0	0	0	0	0	0	0	0
ASV_1928	0	0	0	0	0	0	0	0	0	0	0	0	0
ASV_1929	0	0	0	0	0	0	0	0	0	0	0	0	0
ASV_193	0	0	0	1019	0	0	0	0	0	0	37	0	0
ASV_194	0	0	0	0	0	0	0	0	0	0	0	0	0
ASV_1944	14	0	0	0	0	0	0	0	0	0	0	0	0
ASV_1945	14	0	0	0	0	0	0	0	0	0	0	0	0
ASV_1946	0	0	0	0	0	14	0	0	0	0	0	0	0
ASV_1947	0	0	0	0	0	0	14	0	0	0	0	0	0
ASV_1948	0	0	0	0	0	0	0	14	0	0	0	0	0
ASV_1949	0	0	0	0	0	0	0	0	0	14	0	0	0
ASV_195	0	632	14	0	378	0	0	11	8	0	0	0	0
ASV_1950	0	0	0	0	0	0	0	0	0	14	0	0	0
ASV_1951	0	0	0	0	0	0	0	0	0	14	0	0	0

ASV_1952	0	0	0	0	0	0	0	0	0	14	0	0	0
ASV_1953	0	0	0	0	0	0	0	0	0	0	14	0	0
ASV_1954	0	0	0	0	0	0	0	0	0	0	0	14	0
ASV_1955	0	0	0	0	0	0	0	0	0	0	0	0	0
ASV_1956	0	0	0	0	0	0	0	0	0	0	0	0	0
ASV_196	1040	0	0	0	0	0	0	0	0	0	0	0	0
ASV_197	0	0	0	0	1037	0	0	0	0	0	0	0	0
ASV_1979	13	0	0	0	0	0	0	0	0	0	0	0	0
ASV_1980	0	0	0	0	0	13	0	0	0	0	0	0	0
ASV_1981	0	0	0	0	0	0	0	7	0	0	0	0	0
ASV_1982	0	0	0	0	0	0	0	0	13	0	0	0	0
ASV_1983	0	0	0	0	0	0	0	0	0	13	0	0	0
ASV_1984	0	0	0	0	0	0	0	0	0	13	0	0	0
ASV_1985	0	0	0	0	0	0	0	0	0	0	13	0	0
ASV_1986	0	0	0	0	0	0	0	0	0	0	13	0	0
ASV_1987	0	0	0	0	0	0	0	0	0	0	0	13	0
ASV_1988	0	0	0	0	0	0	0	0	0	0	0	13	0
ASV_1989	0	0	0	0	0	0	0	0	0	0	0	13	0
ASV_1990	0	0	0	0	0	0	0	0	0	0	0	13	0
ASV_1991	0	0	0	0	0	0	0	0	0	0	0	0	13
ASV_1992	0	0	0	0	0	0	0	0	0	0	0	0	0
ASV_1993	0	0	0	0	0	0	0	0	0	0	0	0	0
ASV_1994	0	0	0	0	0	0	0	0	0	0	0	0	0
ASV_2	304	7620	22383	0	0	37430	0	21452	0	0	885	36196	0
ASV_2013	12	0	0	0	0	0	0	0	0	0	0	0	0
ASV_2014	0	0	0	12	0	0	0	0	0	0	0	0	0
ASV_2015	0	0	0	0	0	12	0	0	0	0	0	0	0
ASV_2016	0	0	0	0	0	12	0	0	0	0	0	0	0
ASV_2017	0	0	0	0	0	4	0	4	0	0	0	0	0
ASV_2018	0	0	0	0	0	0	12	0	0	0	0	0	0
ASV_2019	0	0	0	0	0	0	0	12	0	0	0	0	0
ASV_2020	0	0	0	0	0	0	0	12	0	0	0	0	0
ASV_2021	0	0	0	0	0	0	0	12	0	0	0	0	0
ASV_2022	0	0	0	0	0	0	0	0	12	0	0	0	0
ASV_2023	0	0	0	0	0	0	0	0	0	12	0	0	0
ASV_2024	0	0	0	0	0	0	0	0	0	0	12	0	0
ASV_2025	0	0	0	0	0	0	0	0	0	0	12	0	0
ASV_2026	0	0	0	0	0	0	0	0	0	0	0	12	0
ASV_2027	0	0	0	0	0	0	0	0	0	0	0	12	0
ASV_2028	0	0	0	0	0	0	0	0	0	0	0	12	0
ASV_2029	0	0	0	0	0	0	0	0	0	0	0	12	0
ASV_203	255	0	0	0	0	0	0	0	0	0	0	0	37
ASV_2030	0	0	0	0	0	0	0	0	0	0	0	12	0
ASV_2031	0	0	0	0	0	0	0	0	0	0	0	12	0
ASV_2032	0	0	0	0	0	0	0	0	0	0	0	12	0
ASV_2033	0	0	0	0	0	0	0	0	0	0	0	0	12
ASV_2034	0	0	0	0	0	0	0	0	0	0	0	0	0
ASV_2035	0	0	0	0	0	0	0	0	0	0	0	0	0
ASV_2036	0	0	0	0	0	0	0	0	0	0	0	0	0
ASV_2037	0	0	0	0	0	0	0	0	0	0	0	0	0
ASV_2038	0	0	0	0	0	0	0	0	0	0	0	0	0
ASV_204	0	0	0	0	0	0	0	0	0	0	982	0	0
ASV_206	0	0	0	0	0	0	0	0	0	0	980	0	0
ASV_2067	11	0	0	0	0	0	0	0	0	0	0	0	0
ASV_2068	11	0	0	0	0	0	0	0	0	0	0	0	0
ASV_2069	0	11	0	0	0	0	0	0	0	0	0	0	0

ASV_2070	0	0	0	0	0	11	0	0	0	0	0	0	0
ASV_2071	0	0	0	0	0	11	0	0	0	0	0	0	0
ASV_2072	0	0	0	0	0	0	0	11	0	0	0	0	0
ASV_2073	0	0	0	0	0	0	0	11	0	0	0	0	0
ASV_2074	0	0	0	0	0	0	0	11	0	0	0	0	0
ASV_2075	0	0	0	0	0	0	0	0	11	0	0	0	0
ASV_2076	0	0	0	0	0	0	0	0	11	0	0	0	0
ASV_2077	0	0	0	0	0	0	0	0	11	0	0	0	0
ASV_2078	0	0	0	0	0	0	0	0	11	0	0	0	0
ASV_2079	0	0	0	0	0	0	0	0	0	11	0	0	0
ASV_208	0	0	0	0	951	0	0	0	0	0	0	0	0
ASV_2080	0	0	0	0	0	0	0	0	0	11	0	0	0
ASV_2081	0	0	0	0	0	0	0	0	0	11	0	0	0
ASV_2082	0	0	0	0	0	0	0	0	0	0	11	0	0
ASV_2083	0	0	0	0	0	0	0	0	0	0	11	0	0
ASV_2084	0	0	0	0	0	0	0	0	0	0	0	11	0
ASV_2085	0	0	0	0	0	0	0	0	0	0	0	11	0
ASV_2086	0	0	0	0	0	0	0	0	0	0	0	0	0
ASV_2087	0	0	0	0	0	0	0	0	0	0	0	0	0
ASV_2088	0	0	0	0	0	0	0	0	0	0	0	0	0
ASV_2089	0	0	0	0	0	0	0	0	0	0	0	0	0
ASV_2090	0	0	0	0	0	0	0	0	0	0	0	0	0
ASV_2091	0	0	0	0	0	0	0	0	0	0	0	0	0
ASV_2092	0	0	0	0	0	0	0	0	0	0	0	0	0
ASV_2093	0	0	0	0	0	0	0	0	0	0	0	0	0
ASV_210	0	14	0	0	0	0	0	158	107	62	0	0	0
ASV_2114	10	0	0	0	0	0	0	0	0	0	0	0	0
ASV_2115	10	0	0	0	0	0	0	0	0	0	0	0	0
ASV_2116	10	0	0	0	0	0	0	0	0	0	0	0	0
ASV_2117	0	0	0	0	0	10	0	0	0	0	0	0	0
ASV_2118	0	0	0	0	0	10	0	0	0	0	0	0	0
ASV_2119	0	0	0	0	0	0	10	0	0	0	0	0	0
ASV_212	0	0	0	0	0	0	0	0	0	0	0	0	0
ASV_2120	0	0	0	0	0	0	0	10	0	0	0	0	0
ASV_2121	0	0	0	0	0	0	0	10	0	0	0	0	0
ASV_2122	0	0	0	0	0	0	0	0	10	0	0	0	0
ASV_2123	0	0	0	0	0	0	0	0	10	0	0	0	0
ASV_2124	0	0	0	0	0	0	0	0	0	0	10	0	0
ASV_2125	0	0	0	0	0	0	0	0	0	0	10	0	0
ASV_2126	0	0	0	0	0	0	0	0	0	0	10	0	0
ASV_2127	0	0	0	0	0	0	0	0	0	0	10	0	0
ASV_2128	0	0	0	0	0	0	0	0	0	0	0	10	0
ASV_2129	0	0	0	0	0	0	0	0	0	0	0	10	0
ASV_213	0	3	0	2	0	0	0	0	7	67	9	0	0
ASV_2130	0	0	0	0	0	0	0	0	0	0	0	0	10
ASV_2131	0	0	0	0	0	0	0	0	0	0	0	0	0
ASV_2132	0	0	0	0	0	0	0	0	0	0	0	0	0
ASV_2133	0	0	0	0	0	0	0	0	0	0	0	0	0
ASV_2134	0	0	0	0	0	0	0	0	0	0	0	0	0
ASV_2135	0	0	0	0	0	0	0	0	0	0	0	0	0
ASV_2136	0	0	0	0	0	0	0	0	0	0	0	0	0
ASV_214	0	0	0	0	0	39	0	336	0	413	0	0	0
ASV_216	0	0	0	0	0	0	778	70	0	0	0	0	0
ASV_218	0	123	0	0	721	0	0	0	0	0	0	0	0
ASV_221	838	0	0	0	0	0	0	0	0	0	0	0	0
ASV_222	0	0	73	0	0	90	0	82	83	0	0	87	52

ASV_226	488	0	0	0	2	0	0	279	28	0	17	0	0
ASV_228	0	0	0	0	0	0	0	0	0	0	817	0	0
ASV_23	0	0	0	290	0	23	0	0	0	0	36307	0	0
ASV_230	0	0	0	0	0	0	0	0	0	41	0	0	0
ASV_231	0	798	0	0	0	0	0	0	0	0	0	0	0
ASV_232	514	0	279	2	0	0	0	0	0	0	10	0	0
ASV_233	64	0	0	86	0	0	0	0	0	0	57	0	0
ASV_235	0	0	0	0	0	0	0	765	0	0	5	5	0
ASV_236	0	0	0	0	0	0	0	0	0	0	770	0	0
ASV_239	0	28	43	541	146	0	0	0	0	0	0	0	0
ASV_240	0	0	0	0	0	86	0	0	0	0	0	0	674
ASV_241	7	0	0	0	2	0	0	3	0	0	0	0	0
ASV_243	223	0	40	380	0	0	0	62	0	0	0	12	0
ASV_244	0	0	0	0	0	0	0	0	0	0	0	0	0
ASV_246	0	580	0	0	0	0	0	0	0	0	0	0	0
ASV_247	0	0	0	0	0	0	0	0	0	0	748	0	0
ASV_248	0	741	0	0	0	0	0	0	0	0	0	0	0
ASV_249	0	730	0	0	0	0	0	0	0	0	5	0	0
ASV_25	34	12	0	52	32859	6	0	0	101	30	232	13	0
ASV_250	0	0	0	0	0	0	0	0	0	724	0	0	0
ASV_252	0	0	0	0	0	0	0	0	0	0	728	0	0
ASV_254	714	0	0	6	0	0	0	0	0	0	0	0	0
ASV_255	0	0	0	0	0	0	0	0	0	0	0	717	0
ASV_261	0	0	0	703	0	0	0	0	0	0	0	0	0
ASV_262	0	0	0	449	0	137	0	0	0	55	0	25	26
ASV_263	0	0	0	0	0	0	0	0	0	0	691	0	0
ASV_264	0	0	0	0	0	0	0	0	0	0	0	0	0
ASV_268	0	0	0	0	0	0	0	666	0	0	0	0	0
ASV_269	0	0	0	0	0	0	0	661	0	0	8	0	0
ASV_270	0	0	0	0	664	0	0	0	0	0	0	0	0
ASV_271	0	0	0	0	0	0	0	0	0	0	661	0	0
ASV_274	0	436	213	0	0	0	0	0	0	0	0	0	0
ASV_275	0	149	0	500	0	0	0	0	0	0	0	0	0
ASV_276	229	0	0	165	0	0	27	53	0	0	0	27	0
ASV_278	0	0	0	0	0	0	0	0	0	0	635	0	0
ASV_28	0	37	0	0	0	0	747	85	28233	0	76	0	0
ASV_280	0	0	0	0	0	0	0	180	82	335	0	0	0
ASV_283	0	0	0	0	0	0	0	6	0	0	0	236	364
ASV_284	0	0	0	0	0	0	0	0	0	0	0	0	0
ASV_286	0	602	0	0	0	0	0	0	7	0	0	0	0
ASV_287	0	0	0	0	3	0	0	0	0	603	7	0	0
ASV_288	339	0	0	0	0	0	0	0	0	269	0	0	0
ASV_292	0	0	0	0	0	0	0	0	0	0	0	0	0
ASV_296	0	0	0	0	0	0	0	0	0	0	589	0	0
ASV_297	0	564	0	0	0	0	0	0	0	0	0	0	0
ASV_3	0	0	0	62957	0	4691	686	1579	904	2071	5309	0	0
ASV_304	0	0	0	0	0	0	0	368	3	0	0	0	0
ASV_306	0	0	552	0	0	0	0	18	0	0	0	0	0
ASV_309	0	0	0	0	0	0	0	0	0	0	558	0	0
ASV_312	9	0	0	0	0	0	0	0	0	0	0	0	0
ASV_314	0	128	0	0	0	72	58	136	60	51	15	0	0
ASV_315	0	0	0	0	0	0	0	0	0	0	0	0	0
ASV_317	0	0	0	527	0	0	0	0	0	0	0	0	0
ASV_325	0	0	0	495	0	0	0	0	0	0	0	0	0
ASV_326	0	494	0	0	0	0	0	0	0	0	0	0	0
ASV_327	0	0	0	0	0	0	0	0	0	0	0	0	0

ASV_328	0	0	0	484	0	0	0	0	0	0	0	0	0
ASV_329	0	0	0	0	0	0	0	0	0	0	482	0	0
ASV_330	446	0	12	0	0	0	0	0	0	0	16	7	0
ASV_331	0	0	0	481	0	0	0	0	0	0	0	0	0
ASV_332	0	0	0	0	480	0	0	0	0	0	0	0	0
ASV_333	0	180	15	59	210	0	6	0	0	0	0	0	8
ASV_335	0	0	0	326	0	0	0	0	0	0	0	128	0
ASV_336	0	0	0	0	0	86	320	0	5	0	0	0	0
ASV_337	0	471	0	0	0	0	0	0	0	0	0	0	0
ASV_338	0	0	0	0	471	0	0	0	0	0	0	0	0
ASV_34	34	20431	591	3084	2293	32	0	0	44	98	75	0	4
ASV_340	0	0	0	0	0	0	0	0	0	0	10	0	0
ASV_341	106	0	21	256	0	0	0	5	8	45	25	0	0
ASV_343	0	0	0	451	0	0	0	0	11	0	0	0	0
ASV_344	0	0	0	0	0	111	0	109	0	0	0	130	89
ASV_347	0	452	0	0	0	0	0	0	0	0	0	0	0
ASV_35	25354	3	0	73	0	0	0	0	41	0	207	0	0
ASV_351	0	0	0	441	0	0	0	0	0	0	0	0	0
ASV_353	0	0	0	0	0	0	0	0	0	0	434	0	0
ASV_357	0	430	0	0	0	0	0	0	0	0	0	0	0
ASV_36	0	0	0	0	0	0	0	0	0	0	0	0	0
ASV_360	0	0	0	320	77	0	0	0	0	0	0	0	0
ASV_361	0	426	0	0	0	0	0	0	0	0	0	0	0
ASV_362	0	0	0	0	418	0	0	0	0	0	0	0	0
ASV_363	0	0	0	0	0	0	0	0	0	0	0	0	0
ASV_364	0	0	0	0	0	0	0	0	0	0	0	0	0
ASV_365	0	0	0	0	0	0	0	0	3	0	0	0	0
ASV_366	0	0	0	0	0	0	0	191	0	0	7	0	0
ASV_369	0	79	0	213	0	0	0	0	0	116	4	0	0
ASV_37	6635	0	0	0	0	3	3622	1	20	13733	53	0	0
ASV_370	0	0	0	412	0	0	0	0	0	0	0	0	0
ASV_371	0	393	0	0	0	16	0	0	0	0	0	0	0
ASV_375	40	0	6	347	0	0	0	0	0	0	9	0	0
ASV_380	212	0	179	0	0	0	0	0	0	0	0	0	0
ASV_381	122	0	0	224	0	12	0	0	0	0	38	0	0
ASV_382	0	0	0	0	0	0	0	0	0	0	394	0	0
ASV_383	393	0	0	0	0	0	0	0	0	0	0	0	0
ASV_387	0	0	0	0	0	0	0	0	0	0	382	0	0
ASV_392	374	0	0	0	0	0	0	0	0	0	0	0	0
ASV_394	0	0	0	0	0	0	0	0	0	0	371	0	0
ASV_396	0	0	0	0	0	0	0	0	0	0	0	0	0
ASV_400	0	0	0	0	362	0	0	0	0	0	0	0	0
ASV_403	0	0	0	0	357	0	0	0	0	0	0	0	0
ASV_404	0	0	0	359	0	0	0	0	0	0	0	0	0
ASV_408	0	351	0	0	0	0	0	0	0	0	0	0	0
ASV_409	0	0	0	0	0	0	0	0	0	0	351	0	0
ASV_414	0	0	0	0	343	0	0	0	0	0	0	0	0
ASV_415	331	0	0	0	0	0	0	0	0	0	10	0	0
ASV_418	0	0	0	0	0	0	0	0	0	0	0	0	0
ASV_419	0	0	0	0	0	0	0	0	0	0	0	0	0
ASV_42	19391	13	0	75	3	1	0	0	44	0	154	0	0
ASV_420	0	0	0	0	0	0	0	0	0	0	0	0	0
ASV_421	0	0	0	0	334	0	0	0	0	0	0	0	0
ASV_425	0	0	330	0	0	0	0	0	0	0	0	0	0
ASV_428	181	0	135	0	0	0	0	0	0	0	6	0	0
ASV_431	0	0	0	0	0	0	0	0	0	35	0	0	0

ASV_433	0	0	0	305	0	0	0	0	0	0	0	0	0
ASV_434	0	0	0	0	0	0	0	0	0	0	316	0	0
ASV_435	0	0	0	0	314	0	0	0	0	0	0	0	0
ASV_436	0	185	0	0	125	0	0	0	0	0	0	0	0
ASV_437	0	0	0	0	0	0	240	0	0	0	0	0	0
ASV_438	16	0	95	75	0	14	27	0	0	82	0	0	0
ASV_44	73	7598	281	1023	7590	26	0	65	44	281	61	41	77
ASV_440	0	65	0	147	0	0	0	0	0	94	0	0	0
ASV_442	0	0	0	304	0	0	0	0	0	0	0	0	0
ASV_447	0	0	0	0	301	0	0	0	0	0	0	0	0
ASV_448	0	0	0	0	0	0	0	0	112	0	0	0	0
ASV_449	0	0	0	0	0	0	0	0	0	0	0	0	0
ASV_45	3519	54	0	0	0	0	2109	0	0	9740	87	0	0
ASV_450	0	296	0	0	0	0	0	0	0	0	0	0	0
ASV_451	295	0	0	0	0	0	0	0	0	0	0	0	0
ASV_455	0	0	288	0	0	0	0	0	0	0	0	0	0
ASV_457	0	0	0	0	0	0	0	0	0	6	0	0	0
ASV_46	0	11721	311	1975	1210	0	0	0	0	101	0	0	0
ASV_463	0	0	0	0	0	0	278	0	0	0	0	0	0
ASV_464	0	0	0	0	0	0	0	0	0	0	0	0	0
ASV_465	0	0	0	263	0	13	0	0	0	0	0	0	0
ASV_466	0	0	0	0	0	0	0	0	0	0	276	0	0
ASV_467	0	0	0	275	0	0	0	0	0	0	0	0	0
ASV_468	0	0	0	0	0	0	0	0	81	0	0	0	0
ASV_469	0	0	0	0	0	0	0	0	0	0	273	0	0
ASV_47	0	0	0	0	15038	0	0	0	73	50	0	0	0
ASV_472	0	0	0	0	0	0	0	0	0	0	0	0	0
ASV_473	0	0	0	270	0	0	0	0	0	0	0	0	0
ASV_474	0	0	0	0	0	0	0	0	0	0	270	0	0
ASV_475	0	0	0	0	0	0	0	0	0	0	0	0	0
ASV_477	0	0	266	0	0	0	0	0	0	0	0	0	0
ASV_478	0	0	0	0	0	0	0	0	0	0	84	0	0
ASV_479	0	0	0	0	0	0	0	0	0	0	0	0	0
ASV_483	0	0	0	0	0	0	0	102	0	0	118	0	0
ASV_486	260	0	0	0	0	0	0	0	0	0	0	0	0
ASV_487	0	87	0	89	74	0	9	0	0	0	0	0	0
ASV_488	0	0	0	39	0	0	0	0	0	0	28	0	0
ASV_490	254	0	0	0	0	0	0	0	0	0	0	0	0
ASV_491	0	0	0	0	0	0	0	0	0	0	0	0	0
ASV_495	235	0	0	0	0	0	0	0	0	0	16	0	0
ASV_5	0	0	0	32100	0	1914	488	724	566	1653	3456	0	0
ASV_501	0	0	0	0	0	0	218	0	0	0	0	25	0
ASV_502	0	0	0	0	0	0	0	0	65	0	14	0	0
ASV_504	0	0	0	0	0	0	0	0	0	0	239	0	0
ASV_505	0	0	0	0	0	0	0	0	0	0	0	0	0
ASV_506	0	0	0	236	0	0	0	0	0	0	0	0	0
ASV_508	230	0	0	0	0	0	0	0	0	0	0	0	0
ASV_509	0	0	0	0	233	0	0	0	0	0	0	0	0
ASV_51	69	0	0	17	0	0	0	38	19	0	12475	10	0
ASV_512	0	231	0	0	0	0	0	0	0	0	0	0	0
ASV_515	0	0	0	0	0	0	0	0	0	0	0	0	0
ASV_52	33	6245	5121	651	12	0	640	0	34	7	60	0	0
ASV_521	0	136	0	0	90	0	0	0	0	0	0	0	0
ASV_523	0	0	0	0	0	4	0	221	0	0	0	0	0
ASV_525	0	0	0	0	0	0	0	0	0	0	0	0	0
ASV_527	0	0	0	79	0	0	0	0	0	0	0	0	0

ASV_528	0	0	0	219	0	0	0	0	0	0	0	0	0
ASV_529	218	0	0	0	0	0	0	0	0	0	0	0	0
ASV_530	0	79	0	0	126	0	0	0	13	0	0	0	0
ASV_532	0	0	0	0	0	0	0	0	95	0	0	0	0
ASV_537	0	0	0	0	0	0	0	0	0	0	0	0	0
ASV_544	0	0	0	0	0	0	0	0	0	0	210	0	0
ASV_545	209	0	0	0	0	0	0	0	0	0	0	0	0
ASV_546	0	0	0	209	0	0	0	0	0	0	0	0	0
ASV_547	0	0	0	0	0	0	209	0	0	0	0	0	0
ASV_548	0	0	0	208	0	0	0	0	0	0	0	0	0
ASV_549	0	0	0	0	0	0	149	0	0	0	59	0	0
ASV_553	0	0	0	206	0	0	0	0	0	0	0	0	0
ASV_555	194	0	0	0	0	0	0	0	0	0	2	0	0
ASV_556	0	0	0	0	0	0	0	0	25	174	0	6	0
ASV_558	0	0	0	204	0	0	0	0	0	0	0	0	0
ASV_56	10054	0	15	0	0	0	0	0	0	0	117	0	10
ASV_565	0	0	0	0	3	0	0	0	0	0	0	111	0
ASV_566	0	0	0	0	0	0	200	0	0	0	0	0	0
ASV_567	0	0	0	0	0	0	200	0	0	0	0	0	0
ASV_568	0	0	0	0	199	0	0	0	0	0	0	0	0
ASV_569	0	0	0	0	0	0	0	0	0	0	198	0	0
ASV_57	41	29	18	28	17	0	0	20	53	0	78	13	12
ASV_571	0	0	0	0	0	0	0	0	0	0	196	0	0
ASV_572	195	0	0	0	0	0	0	0	0	0	0	0	0
ASV_575	0	0	0	0	194	0	0	0	0	0	0	0	0
ASV_577	0	0	192	0	0	0	0	0	0	0	0	0	0
ASV_58	0	19	0	0	31	13	0	0	0	0	9888	0	0
ASV_581	0	190	0	0	0	0	0	0	0	0	0	0	0
ASV_587	0	83	0	101	0	0	0	0	0	0	0	0	0
ASV_588	0	0	0	0	0	0	0	0	0	0	184	0	0
ASV_589	183	0	0	0	0	0	0	0	0	0	0	0	0
ASV_59	0	0	0	0	0	0	0	0	0	0	0	0	0
ASV_590	0	67	0	0	0	0	0	54	0	36	0	3	23
ASV_593	0	0	0	182	0	0	0	0	0	0	0	0	0
ASV_596	0	0	0	0	0	0	0	0	0	0	0	0	0
ASV_6	147	4144	6872	0	0	9124	0	2624	5788	0	515	4995	2815
ASV_60	0	0	0	0	0	0	0	202	287	0	0	5701	0
ASV_601	10	99	0	0	0	0	0	58	0	0	0	0	0
ASV_602	0	177	0	0	0	0	0	0	0	0	0	0	0
ASV_603	0	0	0	0	0	0	0	0	0	177	0	0	0
ASV_604	0	0	0	0	0	0	0	0	0	0	0	0	0
ASV_605	0	0	0	0	0	0	0	0	0	0	0	0	0
ASV_611	174	0	0	0	0	0	0	0	0	0	0	0	0
ASV_612	17	129	0	0	0	0	0	0	0	0	27	0	0
ASV_615	0	0	0	0	0	0	0	0	0	0	0	0	0
ASV_619	0	0	0	0	0	0	171	0	0	0	0	0	0
ASV_62	19	0	0	3	0	0	0	0	0	0	29	0	0
ASV_622	0	0	0	0	0	0	0	170	0	0	0	0	0
ASV_628	0	0	0	167	0	0	0	0	0	0	0	0	0
ASV_630	165	0	0	0	0	0	0	0	0	0	0	0	0
ASV_631	165	0	0	0	0	0	0	0	0	0	0	0	0
ASV_633	161	0	0	0	0	0	0	0	3	0	0	0	0
ASV_634	158	3	0	0	0	0	0	0	0	0	3	0	0
ASV_635	158	0	0	0	0	0	0	0	0	0	6	0	0
ASV_639	0	162	0	0	0	0	0	0	0	0	0	0	0
ASV_643	0	161	0	0	0	0	0	0	0	0	0	0	0

ASV_644	0	0	0	0	0	16	0	64	48	0	0	0	0
ASV_646	0	0	0	0	0	0	0	0	0	0	0	0	0
ASV_647	0	0	0	0	0	0	0	0	0	0	0	0	0
ASV_650	0	0	0	159	0	0	0	0	0	0	0	0	0
ASV_651	0	0	0	0	0	0	0	0	0	0	0	0	159
ASV_652	0	0	0	0	0	0	0	0	0	0	0	0	0
ASV_657	157	0	0	0	0	0	0	0	0	0	0	0	0
ASV_658	0	0	0	0	0	0	157	0	0	0	0	0	0
ASV_66	0	0	0	18	7133	0	0	0	0	0	30	0	0
ASV_661	0	0	0	0	0	0	0	0	0	156	0	0	0
ASV_665	0	155	0	0	0	0	0	0	0	0	0	0	0
ASV_67	0	0	0	7174	15	14	0	0	0	0	0	0	0
ASV_670	0	0	0	0	0	0	0	0	0	0	0	0	0
ASV_671	50	0	101	0	0	0	0	0	0	0	0	0	0
ASV_674	0	150	0	0	0	0	0	0	0	0	0	0	0
ASV_678	0	149	0	0	0	0	0	0	0	0	0	0	0
ASV_679	0	0	0	0	149	0	0	0	0	0	0	0	0
ASV_68	0	0	0	0	0	0	0	0	0	0	0	0	0
ASV_680	0	0	0	0	0	0	0	0	26	58	0	0	0
ASV_683	0	0	0	0	148	0	0	0	0	0	0	0	0
ASV_684	0	0	0	0	0	0	0	0	0	148	0	0	0
ASV_685	0	0	0	0	0	0	0	0	0	0	0	27	121
ASV_686	0	0	0	0	0	0	0	0	0	0	121	26	0
ASV_69	0	3586	2152	356	0	0	429	0	0	0	0	0	0
ASV_690	142	0	0	0	0	0	0	0	0	0	0	0	0
ASV_691	0	0	0	0	0	0	0	146	0	0	0	0	0
ASV_692	0	0	0	0	0	0	0	0	0	0	38	0	0
ASV_702	0	0	0	144	0	0	0	0	0	0	0	0	0
ASV_703	0	0	0	0	0	0	0	0	37	107	0	0	0
ASV_704	0	0	0	0	0	0	0	0	2	7	0	105	0
ASV_705	0	0	0	0	0	0	0	143	0	0	0	0	0
ASV_706	0	0	0	0	0	0	0	0	0	0	143	0	0
ASV_709	0	0	0	0	0	0	142	0	0	0	0	0	0
ASV_710	0	0	0	0	0	0	0	0	0	0	142	0	0
ASV_711	0	0	0	0	0	0	0	0	0	0	0	0	0
ASV_715	135	0	0	0	0	0	0	0	0	0	0	6	0
ASV_716	0	0	92	0	0	0	0	49	0	0	0	0	0
ASV_717	0	0	0	0	0	141	0	0	0	0	0	0	0
ASV_719	0	140	0	0	0	0	0	0	0	0	0	0	0
ASV_720	0	0	0	0	0	0	0	0	0	140	0	0	0
ASV_721	0	0	0	0	0	0	0	0	0	0	140	0	0
ASV_722	0	0	0	0	0	0	0	0	0	0	0	0	140
ASV_723	0	0	0	0	0	0	0	0	0	0	0	0	0
ASV_725	0	0	0	139	0	0	0	0	0	0	0	0	0
ASV_726	0	0	0	0	0	0	0	0	0	0	0	0	0
ASV_727	0	0	0	0	0	0	0	0	0	0	0	0	0
ASV_729	0	0	130	0	0	8	0	0	0	0	0	0	0
ASV_731	0	0	137	0	0	0	0	0	0	0	0	0	0
ASV_732	0	0	0	137	0	0	0	0	0	0	0	0	0
ASV_734	0	0	0	0	0	136	0	0	0	0	0	0	0
ASV_737	0	0	0	119	0	0	0	0	0	0	0	0	0
ASV_738	0	0	0	0	0	0	0	0	0	135	0	0	0
ASV_74	0	0	0	0	0	0	0	0	0	0	11	0	0
ASV_743	0	0	0	0	0	0	0	0	0	134	0	0	0
ASV_744	0	0	0	0	0	0	0	0	0	133	0	0	0
ASV_745	0	0	0	0	0	0	0	0	0	0	133	0	0

ASV_747	0	0	0	0	0	0	0	0	0	0	0	0	0
ASV_748	0	131	0	0	0	0	0	0	0	0	0	0	0
ASV_749	0	0	0	0	0	0	0	0	0	0	0	0	0
ASV_75	14	3384	416	2016	1	0	0	0	20	0	30	0	0
ASV_752	0	0	0	0	0	0	0	0	0	0	0	0	0
ASV_756	118	0	0	0	0	0	0	0	0	0	0	11	0
ASV_757	0	0	0	0	0	0	0	0	0	128	0	0	0
ASV_758	0	0	0	0	0	0	0	0	0	128	0	0	0
ASV_759	0	0	0	0	0	0	0	0	0	0	0	0	0
ASV_76	6	0	0	0	0	0	0	0	18	0	28	0	0
ASV_761	0	0	0	0	0	0	0	0	0	127	0	0	0
ASV_762	0	0	0	0	0	0	0	0	0	0	127	0	0
ASV_763	0	0	0	0	0	0	0	0	0	0	127	0	0
ASV_768	0	0	0	126	0	0	0	0	0	0	0	0	0
ASV_769	0	0	0	0	126	0	0	0	0	0	0	0	0
ASV_77	0	11	0	0	0	0	0	0	15	0	0	0	0
ASV_771	0	0	0	125	0	0	0	0	0	0	0	0	0
ASV_772	124	0	0	0	0	0	0	0	0	0	0	0	0
ASV_773	0	0	0	124	0	0	0	0	0	0	0	0	0
ASV_774	0	0	0	0	124	0	0	0	0	0	0	0	0
ASV_775	0	0	0	0	0	0	0	0	0	0	124	0	0
ASV_779	0	0	0	0	0	0	0	0	0	0	0	0	0
ASV_78	5678	16	0	25	14	0	0	0	14	0	103	0	6
ASV_783	0	0	0	0	0	7	0	67	37	0	0	0	0
ASV_787	0	0	0	0	0	0	121	0	0	0	0	0	0
ASV_795	0	118	0	0	0	0	0	0	0	0	0	0	0
ASV_796	0	0	0	0	0	0	0	118	0	0	0	0	0
ASV_797	0	0	0	0	0	0	0	0	0	118	0	0	0
ASV_798	0	0	0	0	0	0	0	0	0	0	118	0	0
ASV_799	117	0	0	0	0	0	0	0	0	0	0	0	0
ASV_8	66	53	51	26	9	584	1097	2241	58626	191	259	991	25
ASV_80	0	4582	57	425	498	0	0	0	23	53	42	0	0
ASV_800	0	117	0	0	0	0	0	0	0	0	0	0	0
ASV_801	0	0	0	117	0	0	0	0	0	0	0	0	0
ASV_802	0	0	0	0	0	0	0	18	0	75	0	0	0
ASV_808	115	0	0	0	0	0	0	0	0	0	0	0	0
ASV_809	115	0	0	0	0	0	0	0	0	0	0	0	0
ASV_81	24	0	624	0	4802	0	0	0	0	0	0	0	0
ASV_810	0	0	0	0	115	0	0	0	0	0	0	0	0
ASV_814	114	0	0	0	0	0	0	0	0	0	0	0	0
ASV_815	0	0	0	0	0	0	0	0	0	0	0	0	0
ASV_817	0	113	0	0	0	0	0	0	0	0	0	0	0
ASV_818	0	0	0	0	0	0	0	0	0	0	87	0	0
ASV_82	0	0	0	0	0	279	0	148	0	181	0	0	0
ASV_821	0	97	0	0	0	0	0	0	0	10	0	0	5
ASV_822	0	0	0	0	0	0	0	112	0	0	0	0	0
ASV_826	0	0	0	0	0	0	0	0	17	94	0	0	0
ASV_827	0	0	0	0	0	0	0	0	0	0	0	0	0
ASV_838	108	0	0	0	0	0	0	0	0	0	0	0	0
ASV_839	0	108	0	0	0	0	0	0	0	0	0	0	0
ASV_84	30	11	0	0	6	0	0	0	0	0	54	0	5
ASV_840	0	0	0	0	0	0	0	0	0	108	0	0	0
ASV_841	0	0	0	0	0	0	0	0	0	108	0	0	0
ASV_842	0	0	0	0	0	0	0	0	0	0	0	0	108
ASV_843	0	0	0	0	0	0	0	0	0	0	0	0	0
ASV_847	94	0	0	0	0	0	0	0	0	0	13	0	0

ASV_848	0	0	0	0	0	0	0	0	107	0	0	0	0
ASV_85	0	0	0	0	0	0	0	141	165	0	0	2650	0
ASV_852	0	0	0	106	0	0	0	0	0	0	0	0	0
ASV_858	0	0	105	0	0	0	0	0	0	0	0	0	0
ASV_859	0	0	0	0	0	0	0	0	0	0	0	0	0
ASV_861	104	0	0	0	0	0	0	0	0	0	0	0	0
ASV_863	0	0	0	103	0	0	0	0	0	0	0	0	0
ASV_864	0	0	0	0	0	0	0	34	0	69	0	0	0
ASV_865	0	0	0	0	0	0	0	0	41	0	0	0	0
ASV_866	0	0	0	0	0	0	0	0	0	103	0	0	0
ASV_867	0	0	0	0	0	0	0	0	0	0	102	0	0
ASV_870	101	0	0	0	0	0	0	0	0	0	0	0	0
ASV_871	0	0	0	101	0	0	0	0	0	0	0	0	0
ASV_873	0	0	0	0	0	0	0	0	0	0	0	0	0
ASV_876	0	0	0	0	0	16	0	39	44	0	0	0	0
ASV_877	0	0	0	0	0	0	0	0	0	0	0	0	0
ASV_88	0	0	16	0	0	0	0	0	0	0	38	0	0
ASV_880	0	98	0	0	0	0	0	0	0	0	0	0	0
ASV_881	0	0	0	0	0	0	0	0	0	0	0	0	98
ASV_884	91	0	0	0	0	0	0	0	0	0	6	0	0
ASV_885	0	0	0	97	0	0	0	0	0	0	0	0	0
ASV_886	0	0	0	0	0	0	0	0	0	97	0	0	0
ASV_89	0	0	0	4388	0	0	0	0	0	0	36	0	0
ASV_893	0	0	0	0	0	0	0	0	0	0	96	0	0
ASV_895	0	72	0	0	0	0	0	0	0	0	0	0	23
ASV_896	0	0	0	0	95	0	0	0	0	0	0	0	0
ASV_9	19979	390	114	0	0	0	0	0	0	0	0	0	857
ASV_900	0	0	0	0	0	0	0	0	0	0	0	0	0
ASV_907	93	0	0	0	0	0	0	0	0	0	0	0	0
ASV_908	0	0	0	0	0	0	0	93	0	0	0	0	0
ASV_909	0	0	0	0	0	0	0	0	0	0	93	0	0
ASV_91	0	117	0	0	4242	0	0	0	0	0	20	0	0
ASV_910	0	0	0	0	0	0	0	0	0	0	0	0	93
ASV_911	0	0	0	0	0	0	0	0	0	0	0	0	0
ASV_914	0	92	0	0	0	0	0	0	0	0	0	0	0
ASV_915	0	0	0	92	0	0	0	0	0	0	0	0	0
ASV_916	0	0	0	0	0	0	0	0	0	0	92	0	0
ASV_917	0	0	0	0	0	0	0	0	0	0	0	0	0
ASV_918	0	0	0	0	0	0	0	0	0	0	0	0	0
ASV_923	0	0	91	0	0	0	0	0	0	0	0	0	0
ASV_924	0	0	0	91	0	0	0	0	0	0	0	0	0
ASV_925	0	0	0	0	0	0	0	91	0	0	0	0	0
ASV_926	0	0	0	0	0	0	0	0	0	0	0	0	0
ASV_927	0	0	0	0	0	0	0	0	0	0	0	0	0
ASV_934	90	0	0	0	0	0	0	0	0	0	0	0	0
ASV_94	0	0	0	0	0	0	0	0	0	0	4191	0	0
ASV_941	84	0	0	5	0	0	0	0	0	0	0	0	0
ASV_942	0	0	0	0	0	0	0	89	0	0	0	0	0
ASV_943	0	0	0	0	0	0	0	0	0	0	89	0	0
ASV_944	0	0	0	0	0	0	0	0	0	0	7	0	0
ASV_946	0	0	0	88	0	0	0	0	0	0	0	0	0
ASV_947	0	0	0	0	0	0	0	0	51	0	0	0	0
ASV_949	87	0	0	0	0	0	0	0	0	0	0	0	0
ASV_95	0	17	0	0	0	0	0	0	0	0	0	0	0
ASV_950	0	0	87	0	0	0	0	0	0	0	0	0	0
ASV_951	0	0	0	0	0	0	87	0	0	0	0	0	0

ASV_952	0	0	0	0	0	0	0	0	0	0	87	0	0
ASV_953	0	0	0	0	0	0	0	0	0	0	0	0	0
ASV_963	0	86	0	0	0	0	0	0	0	0	0	0	0
ASV_964	0	0	0	86	0	0	0	0	0	0	0	0	0
ASV_965	0	0	0	0	86	0	0	0	0	0	0	0	0
ASV_966	0	0	0	0	0	0	0	86	0	0	0	0	0
ASV_967	0	0	0	0	0	0	0	0	0	0	0	0	86
ASV_969	85	0	0	0	0	0	0	0	0	0	0	0	0
ASV_970	85	0	0	0	0	0	0	0	0	0	0	0	0
ASV_971	0	0	0	0	0	85	0	0	0	0	0	0	0
ASV_972	0	0	0	0	0	0	0	0	85	0	0	0	0
ASV_976	84	0	0	0	0	0	0	0	0	0	0	0	0
ASV_977	81	0	0	0	0	0	0	0	0	0	0	0	0
ASV_978	0	0	0	84	0	0	0	0	0	0	0	0	0
ASV_979	0	0	0	0	0	84	0	0	0	0	0	0	0
ASV_98	0	0	0	0	0	0	0	0	0	3895	0	0	0
ASV_980	0	0	0	0	0	0	0	0	0	84	0	0	0
ASV_981	0	0	0	0	0	0	0	0	0	0	84	0	0
ASV_984	83	0	0	0	0	0	0	0	0	0	0	0	0
ASV_985	0	0	0	83	0	0	0	0	0	0	0	0	0
ASV_986	0	0	0	0	0	0	0	0	0	83	0	0	0
ASV_987	0	0	0	0	0	0	0	0	0	0	0	0	83
ASV_988	0	0	0	0	0	0	0	0	0	0	0	0	83
ASV_989	0	0	0	0	0	0	0	0	0	0	0	0	0
ASV_995	82	0	0	0	0	0	0	0	0	0	0	0	0
ASV_996	0	82	0	0	0	0	0	0	0	0	0	0	0
ASV_997	0	0	0	81	0	0	0	0	0	0	0	0	0
ASV_998	0	0	0	78	0	0	0	0	0	0	3	0	0
ASV_999	0	0	0	0	0	0	0	81	0	0	0	0	0

Table S2. Abundance (number of sequences) data for the metabarcoding of the mycobiota of *Ficus colubrinae* fruits.

ASV	F01.1	F01.2	F01.3	F01.4	F01.5	F02.1	F02.2	F02.3	F02.4
ASV_10	26	16	38941	0	29	0	18	0	0
ASV_100	0	6	0	3542	0	0	9	0	0
ASV_1004	0	0	0	0	0	0	0	0	0
ASV_1005	0	0	0	0	0	0	0	0	0
ASV_1006	0	0	0	0	0	0	0	0	0
ASV_1007	0	0	0	0	0	0	0	0	37
ASV_1008	0	0	0	0	0	0	0	0	0
ASV_1009	0	0	0	0	0	0	80	0	0
ASV_101	0	0	0	0	0	0	3786	0	0
ASV_1014	0	0	0	0	0	0	0	16	0
ASV_1015	0	0	0	0	0	0	0	70	0
ASV_1016	0	0	0	0	0	0	0	24	0
ASV_1017	0	0	0	0	0	0	0	0	0
ASV_1018	0	0	0	0	0	0	0	0	0
ASV_1021	0	0	0	0	0	0	0	0	0
ASV_1022	0	0	0	0	0	0	0	0	0
ASV_1023	0	0	0	0	0	0	0	0	0
ASV_1024	0	0	0	0	0	0	0	0	0
ASV_103	8	0	0	0	0	0	14	0	0
ASV_1030	0	0	0	0	0	0	0	0	0
ASV_1031	0	0	0	0	0	0	0	0	0
ASV_1032	0	0	0	0	0	0	0	0	0
ASV_1033	0	0	0	0	0	0	0	0	0
ASV_1034	0	0	0	77	0	0	0	0	0
ASV_1035	0	0	0	0	0	77	0	0	0
ASV_1040	0	0	0	0	0	0	0	0	0
ASV_1041	0	0	0	0	0	0	0	0	0
ASV_1042	0	0	0	0	0	0	0	0	0
ASV_1045	0	0	0	0	0	0	0	0	0
ASV_1046	0	0	0	0	0	0	0	0	0
ASV_1047	0	0	0	0	0	0	0	0	0
ASV_105	0	0	0	0	0	0	0	0	0
ASV_1054	0	0	0	0	0	0	0	0	0
ASV_1055	0	0	0	0	0	0	0	0	0
ASV_106	1584	1180	593	0	0	0	0	0	0
ASV_1062	0	0	0	0	0	0	0	0	0
ASV_1063	0	0	0	0	0	73	0	0	0
ASV_1064	0	0	0	0	0	0	0	73	0
ASV_1067	0	0	0	0	0	0	0	0	0
ASV_1068	0	0	0	0	0	0	0	0	0
ASV_1069	0	0	0	0	0	0	0	0	0
ASV_107	0	0	15	0	19	0	0	0	7
ASV_1070	0	0	0	0	0	0	0	0	0
ASV_1071	0	0	68	0	0	0	0	0	0
ASV_1078	0	0	0	0	0	0	0	0	0
ASV_1079	0	0	0	0	0	0	0	0	0
ASV_108	21	30	37	12	0	0	0	0	20
ASV_1080	0	0	0	0	0	0	0	0	0
ASV_1081	0	0	0	0	0	0	0	0	0
ASV_1082	0	0	0	0	0	0	0	71	0
ASV_109	0	0	0	0	0	0	0	0	0
ASV_1092	0	0	0	0	0	0	0	0	0
ASV_1093	0	0	0	0	0	0	0	0	0
ASV_1094	0	0	0	0	0	0	0	0	0
ASV_1100	0	0	0	0	0	0	0	0	0
ASV_1101	0	0	0	0	0	0	0	0	0
ASV_1105	0	0	0	0	0	0	0	0	0

ASV_1106	0	0	0	0	0	68	0	0	0
ASV_1109	0	0	0	0	0	0	0	0	0
ASV_1110	0	0	0	0	0	0	0	0	0
ASV_1111	0	0	0	0	0	0	0	0	40
ASV_1112	0	0	0	0	0	0	0	0	0
ASV_1113	0	0	0	0	67	0	0	0	0
ASV_1120	0	0	0	0	0	0	0	0	0
ASV_1121	0	0	0	0	0	0	0	0	0
ASV_1122	0	0	0	0	0	0	0	0	0
ASV_1123	0	0	0	0	0	0	0	0	0
ASV_1124	0	0	0	0	0	0	0	0	0
ASV_1125	0	0	0	0	0	0	0	0	0
ASV_1126	0	0	0	0	0	0	0	0	0
ASV_1127	0	0	66	0	0	0	0	0	0
ASV_1128	0	0	0	0	0	0	66	0	0
ASV_1129	0	0	0	0	0	0	34	17	15
ASV_1130	0	0	0	0	0	0	0	66	0
ASV_1136	0	0	0	0	0	0	0	0	0
ASV_1137	0	0	0	0	0	0	0	0	0
ASV_1138	0	0	0	0	0	0	0	0	0
ASV_1139	0	0	0	0	0	0	0	0	65
ASV_114	0	0	0	0	2663	0	0	0	595
ASV_1142	0	0	0	0	0	0	0	0	0
ASV_1143	0	0	0	0	0	0	0	0	0
ASV_1144	0	0	0	0	0	0	0	0	0
ASV_1145	0	0	0	0	0	0	0	64	0
ASV_115	0	0	0	0	11	0	0	0	0
ASV_1151	0	0	0	0	0	0	0	0	0
ASV_1156	0	0	0	0	0	0	0	0	0
ASV_1157	0	0	0	0	0	0	0	0	0
ASV_1158	0	0	0	0	0	0	0	0	0
ASV_1159	0	0	0	0	0	0	0	0	0
ASV_1160	0	0	0	0	0	0	0	0	4
ASV_1161	0	0	0	0	0	0	0	0	0
ASV_1167	0	0	0	0	0	0	0	0	0
ASV_1168	0	0	0	0	0	0	0	0	0
ASV_1169	0	0	0	0	0	0	0	0	0
ASV_117	1615	975	537	0	0	0	0	0	0
ASV_1170	0	0	0	0	0	0	0	0	0
ASV_1171	0	0	0	0	0	0	0	0	0
ASV_1172	0	0	0	0	0	0	0	61	0
ASV_1181	0	0	0	0	0	0	0	0	0
ASV_1184	0	0	0	0	0	0	0	0	0
ASV_1185	0	0	0	0	0	0	0	0	0
ASV_1186	0	0	0	0	0	0	0	0	0
ASV_1187	0	0	0	0	0	0	0	0	0
ASV_1188	0	0	0	59	0	0	0	0	0
ASV_1189	0	0	0	0	0	0	0	59	0
ASV_1194	0	0	0	0	0	0	0	0	0
ASV_1195	0	0	0	0	0	0	0	0	0
ASV_1196	0	0	0	0	0	0	0	0	0
ASV_1197	0	0	0	0	0	0	0	58	0
ASV_120	11	9	10	0	0	7	0	2906	0
ASV_1204	0	0	0	0	0	0	0	0	0
ASV_1205	0	0	0	0	0	0	0	0	0
ASV_1206	0	0	0	0	0	0	0	0	0
ASV_1207	0	0	0	0	0	0	0	0	0
ASV_1208	0	0	0	0	0	0	0	0	0
ASV_1209	0	0	0	0	0	0	0	0	0
ASV_1210	0	0	0	0	0	0	0	0	0
ASV_1211	0	0	0	0	0	0	0	0	57
ASV_1219	0	0	0	0	0	0	0	0	0

ASV_1220	0	0	0	0	0	0	0	0	0
ASV_1221	0	0	0	0	0	0	0	0	0
ASV_1222	0	0	0	0	0	0	0	0	0
ASV_1223	0	0	0	0	0	0	0	0	0
ASV_1224	0	0	0	0	0	0	0	0	0
ASV_1225	0	0	0	0	0	0	0	0	0
ASV_1226	0	0	0	0	0	0	0	0	0
ASV_1227	0	0	0	0	0	0	0	0	0
ASV_1228	0	0	0	0	0	0	0	56	0
ASV_1229	0	0	0	0	0	0	0	0	56
ASV_123	0	0	0	0	0	286	0	1474	913
ASV_1235	0	0	0	0	0	0	0	0	0
ASV_1236	0	0	0	0	0	0	0	0	0
ASV_1237	0	0	0	0	0	0	0	0	0
ASV_1238	0	0	0	0	0	0	55	0	0
ASV_1242	0	0	0	0	0	0	0	0	0
ASV_1243	0	0	0	0	0	0	0	0	0
ASV_1244	0	0	0	0	0	0	0	0	0
ASV_1245	0	0	0	0	0	0	0	0	0
ASV_1246	0	0	0	0	54	0	0	0	0
ASV_125	0	0	0	0	0	0	0	18	0
ASV_1254	0	0	0	0	0	0	0	0	0
ASV_1255	0	0	0	0	0	0	0	0	0
ASV_1256	0	0	0	0	0	0	0	0	0
ASV_1257	0	0	0	0	0	0	0	0	0
ASV_1258	0	0	0	0	0	0	0	0	0
ASV_1259	0	0	0	0	0	0	0	0	0
ASV_126	2	0	0	0	0	0	12	0	0
ASV_1267	0	0	0	0	0	0	0	0	0
ASV_1268	0	0	0	0	0	0	0	0	0
ASV_1269	0	0	0	0	0	0	0	0	0
ASV_127	0	0	0	0	0	0	0	0	0
ASV_1270	0	0	0	0	0	0	0	0	0
ASV_1271	0	0	0	0	0	0	0	0	0
ASV_1272	0	0	0	0	0	0	0	0	0
ASV_1273	0	0	0	0	0	0	0	0	0
ASV_1274	0	0	0	52	0	0	0	0	0
ASV_1275	0	0	0	0	0	52	0	0	0
ASV_1282	0	0	0	0	0	0	0	0	0
ASV_1283	0	0	0	0	0	0	0	0	0
ASV_1284	0	0	0	0	0	0	0	0	0
ASV_1285	0	0	0	0	0	0	0	0	0
ASV_1289	0	0	0	0	0	50	0	0	0
ASV_1296	0	0	0	0	0	0	0	0	0
ASV_1297	0	0	0	0	0	0	0	0	0
ASV_1298	0	0	0	0	0	0	0	0	0
ASV_1299	0	0	0	0	0	0	0	0	0
ASV_13	0	103	0	0	0	0	0	75	56
ASV_130	0	0	0	0	5	0	0	0	0
ASV_1300	0	0	0	0	0	0	0	0	0
ASV_1301	0	0	0	0	0	0	0	0	0
ASV_1302	0	9	0	0	0	0	0	0	0
ASV_1303	0	0	0	0	0	0	0	0	0
ASV_1304	0	0	0	0	0	0	0	0	0
ASV_1312	0	0	0	0	0	0	0	0	0
ASV_1313	0	0	0	0	0	11	0	0	0
ASV_1314	0	0	0	48	0	0	0	0	0
ASV_1325	0	0	0	0	0	0	0	0	0
ASV_133	23	0	33	2193	0	0	0	0	17
ASV_1331	0	0	0	0	0	0	0	0	0
ASV_1332	0	0	0	0	0	0	0	0	0
ASV_1333	0	0	0	0	0	0	0	0	0

ASV_1334	0	0	0	0	0	46	0	0	0
ASV_134	0	0	0	222	1049	340	0	388	0
ASV_1341	0	0	0	0	0	0	0	0	0
ASV_1342	0	0	0	0	0	0	0	0	0
ASV_1343	0	0	0	0	0	0	0	0	0
ASV_1344	0	0	0	0	0	0	0	0	0
ASV_1345	0	0	0	0	0	0	0	0	0
ASV_1346	0	0	0	0	0	0	0	0	45
ASV_135	0	0	0	0	0	0	14	20	0
ASV_1351	0	0	0	0	0	0	0	0	0
ASV_1352	0	0	0	0	0	0	0	0	0
ASV_1353	0	0	0	0	0	0	0	0	0
ASV_1354	0	0	0	0	0	0	0	0	0
ASV_1355	0	0	0	0	0	0	0	0	0
ASV_1356	0	0	0	0	4	23	0	17	0
ASV_1357	0	0	0	0	0	44	0	0	0
ASV_1358	0	0	0	0	0	0	44	0	0
ASV_1359	0	0	0	0	0	0	0	0	44
ASV_1366	0	0	0	0	0	0	0	0	0
ASV_1367	0	0	0	0	0	6	6	0	11
ASV_1368	0	0	0	0	0	0	43	0	0
ASV_1369	0	0	0	0	0	0	0	43	0
ASV_137	0	0	0	0	0	0	0	0	0
ASV_1373	0	0	0	0	0	0	0	0	0
ASV_1374	0	0	0	0	0	0	0	0	0
ASV_1375	0	0	0	0	0	0	0	0	0
ASV_138	0	2	0	0	0	0	0	2	0
ASV_139	0	0	0	0	0	0	0	0	0
ASV_1390	0	0	0	0	0	0	0	0	0
ASV_1391	0	0	0	0	0	0	0	0	0
ASV_1392	0	0	0	0	0	0	0	41	0
ASV_140	8	0	0	0	0	0	0	0	4
ASV_1400	0	0	0	0	0	0	0	0	0
ASV_1401	0	0	0	0	0	0	0	0	0
ASV_1402	0	0	0	0	0	0	0	0	0
ASV_1403	0	0	0	0	0	0	0	0	0
ASV_1404	0	0	0	0	0	0	0	0	0
ASV_1405	0	0	0	0	0	3	0	0	0
ASV_1412	0	0	0	0	0	0	0	0	0
ASV_1413	0	0	0	0	0	0	0	0	0
ASV_1414	0	0	0	0	0	0	18	0	0
ASV_1415	19	0	0	0	20	0	0	0	0
ASV_1422	0	0	0	0	0	0	0	0	0
ASV_1423	0	0	0	0	0	0	0	0	0
ASV_1424	0	0	0	0	0	0	0	0	0
ASV_1425	0	0	0	0	0	0	0	0	0
ASV_1426	24	0	0	0	0	0	0	0	14
ASV_1427	0	0	0	0	0	0	0	38	0
ASV_1428	0	0	0	0	0	0	0	0	38
ASV_1436	0	0	0	0	0	0	0	0	0
ASV_1437	0	0	0	0	0	0	0	0	0
ASV_1438	0	0	0	0	0	0	0	0	0
ASV_1439	0	0	0	0	0	0	0	0	0
ASV_1440	0	0	0	0	0	0	0	0	0
ASV_1441	0	0	0	0	0	0	0	0	0
ASV_1442	0	0	0	0	0	0	0	0	0
ASV_145	0	0	0	0	0	0	0	0	0
ASV_1459	0	0	0	0	0	0	0	0	0
ASV_1460	0	0	0	0	0	0	0	0	0
ASV_1461	0	0	0	0	0	0	0	0	0
ASV_1462	0	0	0	0	0	0	0	0	0
ASV_1463	0	0	0	0	0	0	18	0	18

ASV_1464	0	0	0	0	0	0	0	36	0
ASV_147	0	0	0	153	303	100	0	472	338
ASV_1472	0	0	0	0	0	0	0	0	0
ASV_1473	0	0	0	0	0	0	0	0	0
ASV_1474	0	0	0	0	0	0	0	0	0
ASV_1475	0	0	0	0	0	0	0	0	0
ASV_1483	0	0	0	0	0	0	0	0	0
ASV_1484	0	0	0	0	0	0	0	0	0
ASV_1485	0	0	0	0	0	0	0	0	0
ASV_1486	0	0	0	0	0	8	0	0	0
ASV_1487	0	0	0	0	0	0	0	0	0
ASV_1488	0	0	0	0	0	0	0	0	0
ASV_1489	0	0	0	0	0	0	0	0	0
ASV_149	0	0	0	0	0	0	0	8	0
ASV_1490	0	0	0	0	0	0	0	29	5
ASV_150	0	0	0	13	0	0	0	0	0
ASV_1500	0	0	0	0	0	0	0	0	0
ASV_1501	0	0	0	0	0	0	0	0	0
ASV_1502	4	6	0	0	0	3	0	20	0
ASV_1503	0	0	0	0	0	33	0	0	0
ASV_1504	0	0	0	0	0	0	0	33	0
ASV_151	0	0	0	0	0	0	0	0	0
ASV_1515	0	0	0	0	0	0	0	0	0
ASV_1516	0	0	0	0	0	0	0	0	0
ASV_1517	0	0	0	0	0	0	0	0	0
ASV_1518	0	0	0	0	0	0	0	0	0
ASV_1519	0	0	0	0	0	0	0	0	0
ASV_152	0	0	0	0	0	233	0	882	624
ASV_1520	0	0	0	0	0	0	0	0	0
ASV_1530	0	0	0	0	0	0	0	0	0
ASV_1531	0	0	0	0	0	0	0	0	0
ASV_1532	0	0	0	0	0	0	0	0	0
ASV_1533	0	0	0	0	0	0	0	0	0
ASV_1534	0	0	0	0	0	0	0	0	0
ASV_1535	0	0	0	0	0	0	0	14	0
ASV_1536	0	31	0	0	0	0	0	0	0
ASV_1537	0	0	0	0	0	31	0	0	0
ASV_154	0	0	0	0	0	0	0	8	0
ASV_1549	0	0	0	0	0	0	0	0	0
ASV_1550	0	0	0	0	0	0	0	0	0
ASV_1551	0	0	0	0	0	0	26	0	0
ASV_1552	0	0	0	0	0	0	0	0	0
ASV_1553	0	0	0	0	0	0	0	0	0
ASV_1554	0	0	0	0	0	0	0	0	0
ASV_1555	0	0	0	0	0	0	0	0	0
ASV_1556	0	0	0	0	0	0	0	12	0
ASV_1557	0	0	0	0	0	0	0	0	0
ASV_1558	0	0	0	0	0	0	0	0	0
ASV_1559	0	0	0	0	0	0	0	0	0
ASV_1560	0	0	0	0	0	0	0	0	7
ASV_1561	30	0	0	0	0	0	0	0	0
ASV_1562	0	0	0	0	30	0	0	0	0
ASV_1563	0	0	0	0	0	0	30	0	0
ASV_157	69	1273	32	0	0	22	0	48	25
ASV_1578	0	0	0	0	0	0	0	0	0
ASV_1579	0	0	0	0	0	0	0	0	0
ASV_1580	0	0	0	0	0	0	0	0	0
ASV_1581	0	0	0	0	0	0	0	0	0
ASV_1582	0	0	0	0	7	0	15	0	0
ASV_1583	0	0	0	0	0	0	0	0	0
ASV_1584	0	0	0	0	0	0	0	0	0
ASV_1585	0	0	0	0	0	0	0	0	0

ASV_1586	0	0	0	0	0	0	0	0	0
ASV_1587	0	0	0	0	0	0	0	0	0
ASV_1588	0	0	0	0	0	0	0	0	0
ASV_159	20	0	0	10	22	0	0	0	0
ASV_1602	0	0	0	0	0	0	0	0	0
ASV_1603	0	0	0	0	0	0	0	0	0
ASV_1604	0	0	0	0	0	0	0	0	0
ASV_1605	0	0	0	0	0	0	0	0	0
ASV_1606	0	0	0	0	0	0	0	0	0
ASV_1607	0	0	0	0	0	0	0	0	0
ASV_1608	0	0	0	0	0	7	0	0	0
ASV_1609	0	0	0	0	0	0	0	0	0
ASV_161	0	0	0	0	0	0	1516	0	0
ASV_1610	0	0	0	0	0	0	0	0	0
ASV_1611	0	0	0	0	0	0	0	0	0
ASV_1612	0	0	0	0	0	0	0	0	0
ASV_1613	0	0	0	0	28	0	0	0	0
ASV_1614	0	0	0	0	0	28	0	0	0
ASV_162	0	0	0	0	0	0	0	0	0
ASV_1626	0	0	0	0	0	0	0	0	0
ASV_1627	0	0	0	0	0	0	0	0	0
ASV_1628	0	0	0	0	0	0	0	0	0
ASV_1629	0	0	0	0	0	0	0	0	0
ASV_163	84	249	13	169	7	0	0	18	249
ASV_1630	0	0	0	0	0	0	0	0	0
ASV_1636	0	0	0	0	0	0	0	0	0
ASV_1637	0	0	0	0	0	0	0	0	0
ASV_1638	0	0	0	0	0	0	0	0	0
ASV_1639	0	0	0	0	0	0	0	0	0
ASV_1640	0	0	0	0	0	0	0	0	0
ASV_1641	0	0	0	0	0	0	0	0	0
ASV_1642	0	0	0	0	0	0	0	0	0
ASV_1643	0	0	0	0	0	0	0	0	0
ASV_1644	0	0	0	0	0	0	0	0	0
ASV_1645	0	0	0	0	0	0	0	0	0
ASV_1646	0	0	26	0	0	0	0	0	0
ASV_1647	0	0	0	0	0	26	0	0	0
ASV_1648	0	0	0	0	0	26	0	0	0
ASV_1649	0	0	0	0	0	0	0	26	0
ASV_166	0	0	0	0	0	0	0	0	0
ASV_1661	0	0	0	0	0	0	0	0	0
ASV_1662	0	0	0	0	0	0	0	0	0
ASV_1663	0	0	0	0	0	0	0	0	0
ASV_1664	0	0	0	0	0	0	0	0	0
ASV_1665	0	0	0	0	0	0	0	0	0
ASV_1666	0	0	0	0	0	0	0	0	0
ASV_1667	0	0	0	0	0	25	0	0	0
ASV_1668	0	0	0	0	0	0	25	0	0
ASV_1669	0	0	0	0	0	0	25	0	0
ASV_1670	0	0	0	0	0	0	0	25	0
ASV_1671	0	0	0	0	0	0	0	0	25
ASV_168	0	7	16	0	4	0	0	10	9
ASV_1684	0	0	0	0	0	0	0	0	0
ASV_1685	0	0	0	0	0	0	0	0	0
ASV_1686	0	0	0	0	0	0	0	0	0
ASV_1687	0	0	0	0	0	0	0	0	0
ASV_1688	0	0	0	0	0	0	0	0	0
ASV_1689	0	24	0	0	0	0	0	0	0
ASV_1690	0	0	0	0	9	0	15	0	0
ASV_17	517	356	191	0	0	0	766	0	0
ASV_170	0	23	0	0	0	0	0	0	0
ASV_1703	0	0	0	0	0	0	0	0	0

ASV_1704	0	0	0	0	0	0	0	0	0
ASV_1705	0	0	0	0	0	0	0	0	0
ASV_1706	0	0	0	0	0	0	0	0	0
ASV_1707	0	0	0	0	0	0	0	0	0
ASV_1708	0	0	0	0	0	0	0	0	0
ASV_1709	0	0	0	0	0	0	0	0	0
ASV_1710	0	0	0	0	0	0	0	0	0
ASV_1711	0	0	0	0	0	0	0	0	0
ASV_1712	0	0	0	0	0	0	0	0	0
ASV_1713	0	0	0	0	0	0	0	0	0
ASV_1714	0	0	0	0	0	0	0	0	0
ASV_1715	0	0	0	0	0	0	0	23	0
ASV_1731	0	0	0	0	0	0	0	0	0
ASV_1732	0	0	0	0	0	0	0	0	0
ASV_1733	0	0	0	0	0	0	0	0	0
ASV_1734	0	0	0	0	0	0	0	0	0
ASV_1735	0	0	0	0	0	0	0	0	0
ASV_1736	0	0	0	0	0	0	0	0	0
ASV_1737	0	0	0	0	0	0	0	0	0
ASV_1738	0	0	0	0	0	0	0	0	0
ASV_1739	0	0	0	0	0	0	0	0	0
ASV_174	0	0	0	0	0	0	0	0	0
ASV_1740	6	0	0	0	0	0	0	0	0
ASV_1741	0	0	0	0	0	0	0	0	0
ASV_1742	0	0	0	0	0	0	0	0	0
ASV_1743	13	0	0	0	0	0	0	9	0
ASV_1744	0	22	0	0	0	0	0	0	0
ASV_1745	0	0	0	0	0	0	22	0	0
ASV_175	0	0	0	0	0	0	0	0	0
ASV_1758	0	0	0	0	0	0	0	0	0
ASV_1759	0	0	0	0	0	0	0	0	0
ASV_1760	0	0	0	0	0	0	0	0	0
ASV_1761	0	0	0	0	0	0	0	0	0
ASV_1762	0	0	0	0	0	0	0	0	0
ASV_1763	0	0	0	0	0	0	0	0	0
ASV_1764	0	0	0	0	0	0	0	0	0
ASV_1765	0	0	0	0	0	0	0	0	0
ASV_1766	0	0	0	0	0	0	0	0	0
ASV_1767	0	0	0	0	0	21	0	0	0
ASV_1768	0	0	0	0	0	21	0	0	0
ASV_1779	0	0	0	0	0	0	0	0	0
ASV_1780	0	0	0	0	0	0	0	0	0
ASV_1781	0	0	0	0	0	0	0	0	0
ASV_1782	0	0	0	0	0	0	0	0	0
ASV_1783	0	0	0	0	0	0	0	0	0
ASV_1784	0	0	0	0	0	0	0	0	0
ASV_1785	0	0	0	0	0	0	4	0	0
ASV_1786	0	0	0	0	0	0	0	0	0
ASV_1787	0	0	0	0	0	0	0	0	0
ASV_1788	0	0	0	0	0	0	0	0	0
ASV_1789	0	0	0	0	0	0	0	20	0
ASV_179	0	0	0	0	0	0	0	10	0
ASV_1790	0	0	0	0	0	0	0	20	0
ASV_1791	0	0	0	0	0	0	0	0	20
ASV_18	21	22	0	0	14	0	12	26	0
ASV_1814	0	0	0	0	0	0	0	0	0
ASV_1815	0	0	0	0	0	0	0	0	0
ASV_1816	0	0	0	0	0	0	0	0	0
ASV_1817	0	0	0	0	0	0	0	0	0
ASV_1818	0	0	0	0	0	0	0	0	0
ASV_1819	0	0	0	0	0	0	0	0	0
ASV_182	0	0	0	0	0	0	0	0	0

ASV_1820	0	0	0	0	0	0	0	0	0
ASV_1821	19	0	0	0	0	0	0	0	0
ASV_1822	0	0	0	0	0	0	19	0	0
ASV_1823	0	0	0	0	0	0	0	19	0
ASV_183	0	0	0	0	0	0	1196	0	0
ASV_1839	0	0	0	0	0	0	0	0	0
ASV_1840	0	0	0	0	0	0	0	0	0
ASV_1841	0	0	0	0	0	0	0	0	0
ASV_1842	0	0	0	0	0	0	0	0	0
ASV_1843	0	0	0	0	0	0	0	0	0
ASV_1844	0	0	0	0	0	0	0	0	0
ASV_1845	0	0	0	0	0	0	0	0	0
ASV_1846	0	0	0	0	0	0	0	0	0
ASV_1847	0	0	0	0	0	0	0	0	0
ASV_1848	0	0	0	0	0	0	0	0	0
ASV_1849	0	0	0	0	0	18	0	0	0
ASV_1850	0	0	0	0	0	0	18	0	0
ASV_186	0	1111	0	0	0	0	0	0	0
ASV_1862	0	0	0	0	0	0	0	0	0
ASV_1863	0	0	0	0	0	0	0	0	0
ASV_1864	0	0	0	0	0	0	0	0	0
ASV_1865	0	0	0	0	0	0	0	0	0
ASV_1866	0	0	0	0	0	0	15	0	0
ASV_1867	0	0	0	0	0	0	0	0	0
ASV_1868	17	0	0	0	0	0	0	0	0
ASV_1869	0	0	0	0	0	0	0	0	17
ASV_1884	0	0	0	0	0	0	0	0	0
ASV_1885	0	0	0	0	0	0	0	0	0
ASV_1886	0	0	0	0	0	0	0	0	0
ASV_1887	0	0	0	0	0	0	0	0	0
ASV_1888	0	0	0	0	0	0	0	0	0
ASV_1889	0	0	0	0	0	0	0	0	0
ASV_1890	0	0	0	0	0	0	0	0	0
ASV_1891	0	0	0	0	0	0	0	0	0
ASV_1892	0	0	0	0	0	0	0	0	0
ASV_1893	0	0	0	0	0	16	0	0	0
ASV_1894	0	0	0	0	0	0	0	16	0
ASV_1895	0	0	0	0	0	0	0	16	0
ASV_19	0	0	0	0	0	0	208	0	0
ASV_190	0	0	0	0	0	0	607	267	191
ASV_1913	0	0	0	0	0	0	7	0	0
ASV_1914	0	0	0	0	0	0	0	0	0
ASV_1915	0	0	0	0	0	0	0	0	0
ASV_1916	0	0	0	0	0	0	0	0	0
ASV_1917	0	0	0	0	0	0	0	0	0
ASV_1918	0	0	0	0	0	0	0	0	0
ASV_1919	0	0	0	0	0	0	0	0	0
ASV_192	0	0	0	0	0	0	690	0	0
ASV_1920	0	0	0	0	0	0	0	0	0
ASV_1921	0	0	0	0	0	0	0	0	0
ASV_1922	0	0	0	0	0	0	0	0	0
ASV_1923	0	0	0	0	0	0	0	0	0
ASV_1924	0	0	0	0	0	0	0	0	0
ASV_1925	0	0	0	0	0	0	0	0	0
ASV_1926	0	0	0	0	0	0	0	0	0
ASV_1927	15	0	0	0	0	0	0	0	0
ASV_1928	15	0	0	0	0	0	0	0	0
ASV_1929	0	0	0	0	0	0	0	0	15
ASV_193	0	0	0	0	0	0	0	0	0
ASV_194	0	0	0	0	0	0	0	1052	0
ASV_1944	0	0	0	0	0	0	0	0	0
ASV_1945	0	0	0	0	0	0	0	0	0

ASV_1946	0	0	0	0	0	0	0	0	0
ASV_1947	0	0	0	0	0	0	0	0	0
ASV_1948	0	0	0	0	0	0	0	0	0
ASV_1949	0	0	0	0	0	0	0	0	0
ASV_195	0	0	0	0	0	0	0	0	0
ASV_1950	0	0	0	0	0	0	0	0	0
ASV_1951	0	0	0	0	0	0	0	0	0
ASV_1952	0	0	0	0	0	0	0	0	0
ASV_1953	0	0	0	0	0	0	0	0	0
ASV_1954	0	0	0	0	0	0	0	0	0
ASV_1955	14	0	0	0	0	0	0	0	0
ASV_1956	0	0	0	0	0	0	14	0	0
ASV_196	0	0	0	0	0	0	0	0	0
ASV_197	0	0	0	0	0	0	0	0	0
ASV_1979	0	0	0	0	0	0	0	0	0
ASV_1980	0	0	0	0	0	0	0	0	0
ASV_1981	6	0	0	0	0	0	0	0	0
ASV_1982	0	0	0	0	0	0	0	0	0
ASV_1983	0	0	0	0	0	0	0	0	0
ASV_1984	0	0	0	0	0	0	0	0	0
ASV_1985	0	0	0	0	0	0	0	0	0
ASV_1986	0	0	0	0	0	0	0	0	0
ASV_1987	0	0	0	0	0	0	0	0	0
ASV_1988	0	0	0	0	0	0	0	0	0
ASV_1989	0	0	0	0	0	0	0	0	0
ASV_1990	0	0	0	0	0	0	0	0	0
ASV_1991	0	0	0	0	0	0	0	0	0
ASV_1992	0	0	0	0	0	0	13	0	0
ASV_1993	0	0	0	0	0	0	13	0	0
ASV_1994	0	0	0	0	0	0	0	13	0
ASV_2	12151	0	0	0	14221	4737	0	35	18951
ASV_2013	0	0	0	0	0	0	0	0	0
ASV_2014	0	0	0	0	0	0	0	0	0
ASV_2015	0	0	0	0	0	0	0	0	0
ASV_2016	0	0	0	0	0	0	0	0	0
ASV_2017	0	0	0	0	0	0	0	4	0
ASV_2018	0	0	0	0	0	0	0	0	0
ASV_2019	0	0	0	0	0	0	0	0	0
ASV_2020	0	0	0	0	0	0	0	0	0
ASV_2021	0	0	0	0	0	0	0	0	0
ASV_2022	0	0	0	0	0	0	0	0	0
ASV_2023	0	0	0	0	0	0	0	0	0
ASV_2024	0	0	0	0	0	0	0	0	0
ASV_2025	0	0	0	0	0	0	0	0	0
ASV_2026	0	0	0	0	0	0	0	0	0
ASV_2027	0	0	0	0	0	0	0	0	0
ASV_2028	0	0	0	0	0	0	0	0	0
ASV_2029	0	0	0	0	0	0	0	0	0
ASV_203	0	0	0	113	230	85	0	278	0
ASV_2030	0	0	0	0	0	0	0	0	0
ASV_2031	0	0	0	0	0	0	0	0	0
ASV_2032	0	0	0	0	0	0	0	0	0
ASV_2033	0	0	0	0	0	0	0	0	0
ASV_2034	12	0	0	0	0	0	0	0	0
ASV_2035	0	0	0	0	0	12	0	0	0
ASV_2036	0	0	0	0	0	0	0	12	0
ASV_2037	0	0	0	0	0	0	0	0	12
ASV_2038	0	0	0	0	0	0	0	0	12
ASV_204	0	0	0	0	0	0	0	0	0
ASV_206	0	0	0	0	0	0	0	0	0
ASV_2067	0	0	0	0	0	0	0	0	0
ASV_2068	0	0	0	0	0	0	0	0	0

ASV_2069	0	0	0	0	0	0	0	0	0
ASV_2070	0	0	0	0	0	0	0	0	0
ASV_2071	0	0	0	0	0	0	0	0	0
ASV_2072	0	0	0	0	0	0	0	0	0
ASV_2073	0	0	0	0	0	0	0	0	0
ASV_2074	0	0	0	0	0	0	0	0	0
ASV_2075	0	0	0	0	0	0	0	0	0
ASV_2076	0	0	0	0	0	0	0	0	0
ASV_2077	0	0	0	0	0	0	0	0	0
ASV_2078	0	0	0	0	0	0	0	0	0
ASV_2079	0	0	0	0	0	0	0	0	0
ASV_208	0	0	0	28	0	0	0	0	0
ASV_2080	0	0	0	0	0	0	0	0	0
ASV_2081	0	0	0	0	0	0	0	0	0
ASV_2082	0	0	0	0	0	0	0	0	0
ASV_2083	0	0	0	0	0	0	0	0	0
ASV_2084	0	0	0	0	0	0	0	0	0
ASV_2085	0	0	0	0	0	0	0	0	0
ASV_2086	11	0	0	0	0	0	0	0	0
ASV_2087	0	0	0	0	0	11	0	0	0
ASV_2088	0	0	0	0	0	11	0	0	0
ASV_2089	0	0	0	0	0	11	0	0	0
ASV_2090	0	0	0	0	0	11	0	0	0
ASV_2091	0	0	0	0	0	11	0	0	0
ASV_2092	0	0	0	0	0	0	0	11	0
ASV_2093	0	0	0	0	0	0	0	11	0
ASV_210	71	201	0	130	0	9	0	0	195
ASV_2114	0	0	0	0	0	0	0	0	0
ASV_2115	0	0	0	0	0	0	0	0	0
ASV_2116	0	0	0	0	0	0	0	0	0
ASV_2117	0	0	0	0	0	0	0	0	0
ASV_2118	0	0	0	0	0	0	0	0	0
ASV_2119	0	0	0	0	0	0	0	0	0
ASV_212	0	887	0	0	10	0	0	0	0
ASV_2120	0	0	0	0	0	0	0	0	0
ASV_2121	0	0	0	0	0	0	0	0	0
ASV_2122	0	0	0	0	0	0	0	0	0
ASV_2123	0	0	0	0	0	0	0	0	0
ASV_2124	0	0	0	0	0	0	0	0	0
ASV_2125	0	0	0	0	0	0	0	0	0
ASV_2126	0	0	0	0	0	0	0	0	0
ASV_2127	0	0	0	0	0	0	0	0	0
ASV_2128	0	0	0	0	0	0	0	0	0
ASV_2129	0	0	0	0	0	0	0	0	0
ASV_213	23	73	435	0	0	0	0	229	0
ASV_2130	0	0	0	0	0	0	0	0	0
ASV_2131	0	0	10	0	0	0	0	0	0
ASV_2132	0	0	10	0	0	0	0	0	0
ASV_2133	0	0	0	0	0	0	0	10	0
ASV_2134	0	0	0	0	0	0	0	10	0
ASV_2135	0	0	0	0	0	0	0	10	0
ASV_2136	0	0	0	0	0	0	0	10	0
ASV_214	0	48	47	0	0	0	0	0	0
ASV_216	0	0	0	0	0	0	0	0	0
ASV_218	0	0	0	0	0	0	0	0	0
ASV_221	0	0	0	0	0	0	0	0	0
ASV_222	96	63	68	0	53	51	0	0	39
ASV_226	0	0	0	0	0	0	6	4	0
ASV_228	0	0	0	0	0	0	0	0	0
ASV_23	349	252	130	0	0	0	504	0	0
ASV_230	0	0	0	767	0	0	0	0	0
ASV_231	0	0	0	0	0	0	0	0	8

ASV_232	0	0	0	0	0	0	0	0	0
ASV_233	73	73	81	49	40	29	89	92	44
ASV_235	0	0	0	0	0	0	0	0	0
ASV_236	0	0	0	0	0	0	0	0	0
ASV_239	0	0	0	0	0	0	0	0	4
ASV_240	0	0	0	0	0	0	0	0	0
ASV_241	0	0	6	0	0	0	741	0	0
ASV_243	0	0	0	0	0	24	0	0	10
ASV_244	0	751	0	0	0	0	0	0	0
ASV_246	0	0	0	19	0	0	0	0	0
ASV_247	0	0	0	0	0	0	0	0	0
ASV_248	0	0	0	0	0	0	0	0	0
ASV_249	0	3	0	0	0	0	0	0	0
ASV_25	48	47	38	0	17	9	61	0	33
ASV_250	0	7	0	0	0	0	3	0	0
ASV_252	0	0	0	0	0	0	0	0	0
ASV_254	0	0	0	0	0	0	0	0	0
ASV_255	0	0	0	0	0	0	0	0	0
ASV_261	0	0	0	0	0	0	0	0	0
ASV_262	0	0	0	0	0	0	0	0	0
ASV_263	0	0	0	0	0	0	0	0	0
ASV_264	0	10	0	0	0	43	13	613	0
ASV_268	0	0	3	0	0	0	0	0	0
ASV_269	0	0	0	0	0	0	0	0	0
ASV_270	0	0	0	0	0	0	0	0	0
ASV_271	0	0	0	0	0	0	0	0	0
ASV_274	0	0	0	0	0	0	0	0	0
ASV_275	0	0	0	0	0	0	0	0	0
ASV_276	0	0	145	0	0	0	0	0	0
ASV_278	0	0	0	0	0	0	0	0	0
ASV_28	0	0	0	0	38	0	0	0	0
ASV_280	0	37	0	0	0	0	0	0	0
ASV_283	0	0	0	0	0	0	19	0	0
ASV_284	0	0	0	0	0	37	588	0	0
ASV_286	0	0	0	0	5	0	0	0	0
ASV_287	0	0	0	0	0	0	0	0	0
ASV_288	0	0	4	0	0	0	0	0	0
ASV_292	0	0	0	0	0	0	598	0	0
ASV_296	0	0	0	0	0	0	0	0	0
ASV_297	0	0	24	0	0	0	0	0	0
ASV_3	56627	40277	18611	0	0	0	87705	0	0
ASV_304	0	0	0	0	0	0	208	0	0
ASV_306	0	0	0	0	0	0	0	0	0
ASV_309	0	0	0	0	0	0	0	0	0
ASV_312	0	70	335	0	0	0	0	139	0
ASV_314	0	4	5	0	0	4	0	8	0
ASV_315	0	0	0	534	0	0	0	0	0
ASV_317	0	0	0	0	0	0	0	0	0
ASV_325	0	0	0	0	0	0	0	0	0
ASV_326	0	0	0	0	0	0	0	0	0
ASV_327	0	0	0	0	0	0	0	302	192
ASV_328	0	0	0	0	0	0	0	0	0
ASV_329	0	0	0	0	0	0	0	0	0
ASV_330	0	0	0	0	0	0	0	0	0
ASV_331	0	0	0	0	0	0	0	0	0
ASV_332	0	0	0	0	0	0	0	0	0
ASV_333	0	0	0	0	0	0	0	0	0
ASV_335	0	0	0	0	0	20	0	0	0
ASV_336	0	0	0	0	0	63	0	0	0
ASV_337	0	0	0	0	0	0	0	0	0
ASV_338	0	0	0	0	0	0	0	0	0
ASV_34	29	56	42	10	41	8	61	37	29

ASV_340	2	0	6	0	0	114	0	185	153
ASV_341	0	0	0	0	0	0	0	0	0
ASV_343	0	0	0	0	0	0	0	0	0
ASV_344	0	0	0	0	0	0	0	19	0
ASV_347	0	0	0	0	0	0	0	0	0
ASV_35	50	36	80	0	33	6	49	79	33
ASV_351	0	0	0	0	0	0	0	0	0
ASV_353	0	0	0	0	0	0	0	0	0
ASV_357	0	0	0	0	0	0	0	0	0
ASV_36	0	0	0	0	0	0	0	0	25743
ASV_360	11	0	0	0	0	0	9	10	0
ASV_361	0	0	0	0	0	0	0	0	0
ASV_362	0	0	0	0	0	0	0	0	0
ASV_363	0	0	0	0	0	0	418	0	0
ASV_364	0	0	0	0	0	0	418	0	0
ASV_365	0	0	0	0	0	0	0	413	0
ASV_366	0	10	0	0	0	6	185	16	0
ASV_369	0	0	0	0	0	0	0	0	0
ASV_37	0	39	102	0	5	0	0	17	0
ASV_370	0	0	0	0	0	0	0	0	0
ASV_371	0	0	0	0	0	0	0	0	0
ASV_375	0	0	0	0	0	0	0	0	0
ASV_380	0	0	0	0	3	0	3	0	0
ASV_381	0	0	0	0	0	0	0	0	0
ASV_382	0	0	0	0	0	0	0	0	0
ASV_383	0	0	0	0	0	0	0	0	0
ASV_387	0	0	0	0	0	0	0	0	0
ASV_392	0	0	0	0	0	0	0	0	0
ASV_394	0	0	0	0	0	0	0	0	0
ASV_396	0	0	0	141	228	0	0	0	0
ASV_400	0	0	0	0	0	0	0	0	0
ASV_403	0	0	0	0	0	0	3	0	0
ASV_404	0	0	0	0	0	0	0	0	0
ASV_408	0	0	0	0	0	0	0	0	0
ASV_409	0	0	0	0	0	0	0	0	0
ASV_414	0	0	0	0	0	0	0	0	0
ASV_415	0	0	0	0	0	0	0	0	0
ASV_418	0	0	0	0	0	220	0	0	118
ASV_419	0	0	0	0	0	191	0	0	144
ASV_42	36	19	59	0	19	0	58	41	29
ASV_420	0	0	0	0	0	0	335	0	0
ASV_421	0	0	0	0	0	0	0	0	0
ASV_425	0	0	0	0	0	0	0	0	0
ASV_428	0	0	0	0	0	0	0	0	4
ASV_431	154	130	0	0	0	0	0	0	0
ASV_433	0	0	3	0	0	0	8	0	0
ASV_434	0	0	0	0	0	0	0	0	0
ASV_435	0	0	0	0	0	0	0	0	0
ASV_436	0	0	0	0	0	0	0	0	0
ASV_437	0	0	0	0	0	70	0	0	0
ASV_438	0	0	0	0	0	0	0	0	0
ASV_44	25	56	44	0	44	0	53	30	22
ASV_440	0	0	0	0	0	0	0	0	0
ASV_442	0	0	0	0	0	0	0	0	0
ASV_447	0	0	0	0	0	0	0	0	0
ASV_448	0	0	185	0	0	0	0	0	0
ASV_449	0	0	0	0	0	0	297	0	0
ASV_45	65	0	0	0	0	0	0	0	0
ASV_450	0	0	0	0	0	0	0	0	0
ASV_451	0	0	0	0	0	0	0	0	0
ASV_455	0	0	0	0	0	0	0	0	0
ASV_457	0	0	0	7	0	88	0	92	93

ASV_46	0	0	0	0	0	0	0	0	0
ASV_463	0	0	0	0	0	0	0	0	0
ASV_464	0	0	0	0	0	0	191	86	0
ASV_465	0	0	0	0	0	0	0	0	0
ASV_466	0	0	0	0	0	0	0	0	0
ASV_467	0	0	0	0	0	0	0	0	0
ASV_468	0	0	144	49	0	0	0	0	0
ASV_469	0	0	0	0	0	0	0	0	0
ASV_47	0	0	0	0	0	0	0	0	0
ASV_472	0	40	0	0	0	83	0	113	35
ASV_473	0	0	0	0	0	0	0	0	0
ASV_474	0	0	0	0	0	0	0	0	0
ASV_475	0	0	0	0	0	26	128	57	58
ASV_477	0	0	0	0	0	0	0	0	0
ASV_478	0	0	0	0	0	58	0	124	0
ASV_479	0	0	0	0	0	0	0	265	0
ASV_483	27	15	0	0	0	0	0	0	0
ASV_486	0	0	0	0	0	0	0	0	0
ASV_487	0	0	0	0	0	0	0	0	0
ASV_488	49	43	0	0	46	0	53	0	0
ASV_490	0	0	0	0	0	0	0	0	0
ASV_491	0	0	0	0	0	0	253	0	0
ASV_495	0	0	0	0	0	0	0	0	0
ASV_5	37424	27567	14008	0	0	0	62477	0	0
ASV_501	0	0	0	0	0	0	0	0	0
ASV_502	0	0	0	0	0	0	163	0	0
ASV_504	0	0	0	0	0	0	0	0	0
ASV_505	238	0	0	0	0	0	0	0	0
ASV_506	0	0	0	0	0	0	0	0	0
ASV_508	5	0	0	0	0	0	0	0	0
ASV_509	0	0	0	0	0	0	0	0	0
ASV_51	37	19	40	3	0	5	55	34	22
ASV_512	0	0	0	0	0	0	0	0	0
ASV_515	0	0	0	0	0	0	230	0	0
ASV_52	0	38	35	24	28	10	0	0	0
ASV_521	0	0	0	0	0	0	0	0	0
ASV_523	0	0	0	0	0	0	0	0	0
ASV_525	0	0	0	0	0	0	221	0	0
ASV_527	0	0	0	0	0	0	0	96	45
ASV_528	0	0	0	0	0	0	0	0	0
ASV_529	0	0	0	0	0	0	0	0	0
ASV_530	0	0	0	0	0	0	0	0	0
ASV_532	0	0	122	0	0	0	0	0	0
ASV_537	0	0	0	0	0	0	93	82	40
ASV_544	0	0	0	0	0	0	0	0	0
ASV_545	0	0	0	0	0	0	0	0	0
ASV_546	0	0	0	0	0	0	0	0	0
ASV_547	0	0	0	0	0	0	0	0	0
ASV_548	0	0	0	0	0	0	0	0	0
ASV_549	0	0	0	0	0	0	0	0	0
ASV_553	0	0	0	0	0	0	0	0	0
ASV_555	0	0	0	0	0	0	0	0	0
ASV_556	0	0	0	0	0	0	0	0	0
ASV_558	0	0	0	0	0	0	0	0	0
ASV_56	0	0	0	0	0	0	0	0	0
ASV_565	0	0	0	0	0	87	0	0	0
ASV_566	0	0	0	0	0	0	0	0	0
ASV_567	0	0	0	0	0	0	0	0	0
ASV_568	0	0	0	0	0	0	0	0	0
ASV_569	0	0	0	0	0	0	0	0	0
ASV_57	56	59	0	0	0	1700	34	7874	33
ASV_571	0	0	0	0	0	0	0	0	0

ASV_572	0	0	0	0	0	0	0	0	0
ASV_575	0	0	0	0	0	0	0	0	0
ASV_577	0	0	0	0	0	0	0	0	0
ASV_58	0	0	0	0	43	0	0	0	0
ASV_581	0	0	0	0	0	0	0	0	0
ASV_587	0	0	0	0	0	0	0	0	0
ASV_588	0	0	0	0	0	0	0	0	0
ASV_589	0	0	0	0	0	0	0	0	0
ASV_59	0	0	9420	0	0	0	0	0	0
ASV_590	0	0	0	0	0	0	0	0	0
ASV_593	0	0	0	0	0	0	0	0	0
ASV_596	0	0	180	0	0	0	0	0	0
ASV_6	12597	0	0	0	11626	2677	0	0	12382
ASV_60	0	0	2567	0	0	0	0	0	0
ASV_601	0	0	0	0	0	0	0	10	0
ASV_602	0	0	0	0	0	0	0	0	0
ASV_603	0	0	0	0	0	0	0	0	0
ASV_604	97	80	0	0	0	0	0	0	0
ASV_605	0	0	0	0	4	0	0	173	0
ASV_611	0	0	0	0	0	0	0	0	0
ASV_612	0	0	0	0	0	0	0	0	0
ASV_615	55	75	42	0	0	0	0	0	0
ASV_619	0	0	0	0	0	0	0	0	0
ASV_62	0	13	0	0	0	0	8240	0	15
ASV_622	0	0	0	0	0	0	0	0	0
ASV_628	0	0	0	0	0	0	0	0	0
ASV_630	0	0	0	0	0	0	0	0	0
ASV_631	0	0	0	0	0	0	0	0	0
ASV_633	0	0	0	0	0	0	0	0	0
ASV_634	0	0	0	0	0	0	0	0	0
ASV_635	0	0	0	0	0	0	0	0	0
ASV_639	0	0	0	0	0	0	0	0	0
ASV_643	0	0	0	0	0	0	0	0	0
ASV_644	0	0	0	0	0	0	0	33	0
ASV_646	0	0	0	0	0	59	0	101	0
ASV_647	0	0	0	0	0	0	0	160	0
ASV_650	0	0	0	0	0	0	0	0	0
ASV_651	0	0	0	0	0	0	0	0	0
ASV_652	159	0	0	0	0	0	0	0	0
ASV_657	0	0	0	0	0	0	0	0	0
ASV_658	0	0	0	0	0	0	0	0	0
ASV_66	29	19	18	0	33	0	0	0	0
ASV_661	0	0	0	0	0	0	0	0	0
ASV_665	0	0	0	0	0	0	0	0	0
ASV_67	0	22	0	0	0	0	0	0	0
ASV_670	0	0	0	0	0	0	153	0	0
ASV_671	0	0	0	0	0	0	0	0	0
ASV_674	0	0	0	0	0	0	0	0	0
ASV_678	0	0	0	0	0	0	0	0	0
ASV_679	0	0	0	0	0	0	0	0	0
ASV_68	0	0	7202	0	0	0	0	0	0
ASV_680	0	0	0	0	0	0	47	18	0
ASV_683	0	0	0	0	0	0	0	0	0
ASV_684	0	0	0	0	0	0	0	0	0
ASV_685	0	0	0	0	0	0	0	0	0
ASV_686	0	0	0	0	0	0	0	0	0
ASV_69	0	0	0	0	0	0	0	0	0
ASV_690	0	0	0	0	0	0	0	0	4
ASV_691	0	0	0	0	0	0	0	0	0
ASV_692	46	0	0	0	0	0	62	0	0
ASV_702	0	0	0	0	0	0	0	0	0
ASV_703	0	0	0	0	0	0	0	0	0

ASV_704	0	0	0	0	0	0	0	30	0
ASV_705	0	0	0	0	0	0	0	0	0
ASV_706	0	0	0	0	0	0	0	0	0
ASV_709	0	0	0	0	0	0	0	0	0
ASV_710	0	0	0	0	0	0	0	0	0
ASV_711	0	0	0	0	0	142	0	0	0
ASV_715	0	0	0	0	0	0	0	0	0
ASV_716	0	0	0	0	0	0	0	0	0
ASV_717	0	0	0	0	0	0	0	0	0
ASV_719	0	0	0	0	0	0	0	0	0
ASV_720	0	0	0	0	0	0	0	0	0
ASV_721	0	0	0	0	0	0	0	0	0
ASV_722	0	0	0	0	0	0	0	0	0
ASV_723	0	0	0	0	0	0	0	87	53
ASV_725	0	0	0	0	0	0	0	0	0
ASV_726	0	0	0	0	0	0	139	0	0
ASV_727	0	0	0	0	0	0	139	0	0
ASV_729	0	0	0	0	0	0	0	0	0
ASV_731	0	0	0	0	0	0	0	0	0
ASV_732	0	0	0	0	0	0	0	0	0
ASV_734	0	0	0	0	0	0	0	0	0
ASV_737	0	0	0	0	0	0	13	3	0
ASV_738	0	0	0	0	0	0	0	0	0
ASV_74	0	0	35	0	7	1389	0	4423	0
ASV_743	0	0	0	0	0	0	0	0	0
ASV_744	0	0	0	0	0	0	0	0	0
ASV_745	0	0	0	0	0	0	0	0	0
ASV_747	0	0	0	0	0	0	0	0	132
ASV_748	0	0	0	0	0	0	0	0	0
ASV_749	0	0	131	0	0	0	0	0	0
ASV_75	0	23	23	0	0	0	0	9	0
ASV_752	0	0	130	0	0	0	0	0	0
ASV_756	0	0	0	0	0	0	0	0	0
ASV_757	0	0	0	0	0	0	0	0	0
ASV_758	0	0	0	0	0	0	0	0	0
ASV_759	0	0	0	0	0	0	0	0	128
ASV_76	8	0	0	5766	3	0	0	0	0
ASV_761	0	0	0	0	0	0	0	0	0
ASV_762	0	0	0	0	0	0	0	0	0
ASV_763	0	0	0	0	0	0	0	0	0
ASV_768	0	0	0	0	0	0	0	0	0
ASV_769	0	0	0	0	0	0	0	0	0
ASV_77	0	0	20	0	7	10	5940	0	0
ASV_771	0	0	0	0	0	0	0	0	0
ASV_772	0	0	0	0	0	0	0	0	0
ASV_773	0	0	0	0	0	0	0	0	0
ASV_774	0	0	0	0	0	0	0	0	0
ASV_775	0	0	0	0	0	0	0	0	0
ASV_779	0	0	0	0	0	0	123	0	0
ASV_78	13	21	20	5	0	9	35	16	11
ASV_783	0	4	0	0	0	0	0	7	0
ASV_787	0	0	0	0	0	0	0	0	0
ASV_795	0	0	0	0	0	0	0	0	0
ASV_796	0	0	0	0	0	0	0	0	0
ASV_797	0	0	0	0	0	0	0	0	0
ASV_798	0	0	0	0	0	0	0	0	0
ASV_799	0	0	0	0	0	0	0	0	0
ASV_8	77	56	52121	0	70	15	27	40	36
ASV_80	0	0	0	0	0	1	15	0	0
ASV_800	0	0	0	0	0	0	0	0	0
ASV_801	0	0	0	0	0	0	0	0	0
ASV_802	0	0	0	0	0	0	0	0	0

ASV_808	0	0	0	0	0	0	0	0	0
ASV_809	0	0	0	0	0	0	0	0	0
ASV_81	0	0	0	0	0	0	0	0	0
ASV_810	0	0	0	0	0	0	0	0	0
ASV_814	0	0	0	0	0	0	0	0	0
ASV_815	0	0	0	0	0	108	0	0	6
ASV_817	0	0	0	0	0	0	0	0	0
ASV_818	0	0	0	0	0	0	26	0	0
ASV_82	2322	1611	759	0	0	0	0	0	0
ASV_821	0	0	0	0	0	0	0	0	0
ASV_822	0	0	0	0	0	0	0	0	0
ASV_826	0	0	0	0	0	0	0	0	0
ASV_827	0	0	0	0	0	111	0	0	0
ASV_838	0	0	0	0	0	0	0	0	0
ASV_839	0	0	0	0	0	0	0	0	0
ASV_84	26	42	0	0	20	0	4953	23	0
ASV_840	0	0	0	0	0	0	0	0	0
ASV_841	0	0	0	0	0	0	0	0	0
ASV_842	0	0	0	0	0	0	0	0	0
ASV_843	0	0	0	0	0	0	0	108	0
ASV_847	0	0	0	0	0	0	0	0	0
ASV_848	0	0	0	0	0	0	0	0	0
ASV_85	0	0	2028	0	0	0	0	0	0
ASV_852	0	0	0	0	0	0	0	0	0
ASV_858	0	0	0	0	0	0	0	0	0
ASV_859	0	0	0	0	0	105	0	0	0
ASV_861	0	0	0	0	0	0	0	0	0
ASV_863	0	0	0	0	0	0	0	0	0
ASV_864	0	0	0	0	0	0	0	0	0
ASV_865	0	0	0	0	0	0	62	0	0
ASV_866	0	0	0	0	0	0	0	0	0
ASV_867	0	0	0	0	0	0	0	0	0
ASV_870	0	0	0	0	0	0	0	0	0
ASV_871	0	0	0	0	0	0	0	0	0
ASV_873	0	0	0	0	100	0	0	0	0
ASV_876	0	0	0	0	0	0	0	0	0
ASV_877	0	0	0	0	0	0	99	0	0
ASV_88	15	14	0	0	3142	0	0	1	929
ASV_880	0	0	0	0	0	0	0	0	0
ASV_881	0	0	0	0	0	0	0	0	0
ASV_884	0	0	0	0	0	0	0	0	0
ASV_885	0	0	0	0	0	0	0	0	0
ASV_886	0	0	0	0	0	0	0	0	0
ASV_89	0	0	0	0	0	0	31	0	0
ASV_893	0	0	0	0	0	0	0	0	0
ASV_895	0	0	0	0	0	0	0	0	0
ASV_896	0	0	0	0	0	0	0	0	0
ASV_9	0	0	0	11566	27350	8330	0	31181	0
ASV_900	0	0	0	0	94	0	0	0	0
ASV_907	0	0	0	0	0	0	0	0	0
ASV_908	0	0	0	0	0	0	0	0	0
ASV_909	0	0	0	0	0	0	0	0	0
ASV_91	0	13	5	0	0	0	13	0	0
ASV_910	0	0	0	0	0	0	0	0	0
ASV_911	0	0	0	0	0	0	0	93	0
ASV_914	0	0	0	0	0	0	0	0	0
ASV_915	0	0	0	0	0	0	0	0	0
ASV_916	0	0	0	0	0	0	0	0	0
ASV_917	0	0	0	0	0	0	92	0	0
ASV_918	0	0	0	0	0	0	0	0	92
ASV_923	0	0	0	0	0	0	0	0	0
ASV_924	0	0	0	0	0	0	0	0	0

ASV_925	0	0	0	0	0	0	0	0	0
ASV_926	0	0	0	0	91	0	0	0	0
ASV_927	0	0	0	0	0	20	59	12	0
ASV_934	0	0	0	0	0	0	0	0	0
ASV_94	0	0	0	0	0	0	0	0	0
ASV_941	0	0	0	0	0	0	0	0	0
ASV_942	0	0	0	0	0	0	0	0	0
ASV_943	0	0	0	0	0	0	0	0	0
ASV_944	0	0	0	0	0	82	0	0	0
ASV_946	0	0	0	0	0	0	0	0	0
ASV_947	0	0	0	0	0	0	0	0	37
ASV_949	0	0	0	0	0	0	0	0	0
ASV_95	0	22	40	3936	0	0	0	12	0
ASV_950	0	0	0	0	0	0	0	0	0
ASV_951	0	0	0	0	0	0	0	0	0
ASV_952	0	0	0	0	0	0	0	0	0
ASV_953	0	0	0	0	0	0	87	0	0
ASV_963	0	0	0	0	0	0	0	0	0
ASV_964	0	0	0	0	0	0	0	0	0
ASV_965	0	0	0	0	0	0	0	0	0
ASV_966	0	0	0	0	0	0	0	0	0
ASV_967	0	0	0	0	0	0	0	0	0
ASV_969	0	0	0	0	0	0	0	0	0
ASV_970	0	0	0	0	0	0	0	0	0
ASV_971	0	0	0	0	0	0	0	0	0
ASV_972	0	0	0	0	0	0	0	0	0
ASV_976	0	0	0	0	0	0	0	0	0
ASV_977	0	0	0	0	0	0	3	0	0
ASV_978	0	0	0	0	0	0	0	0	0
ASV_979	0	0	0	0	0	0	0	0	0
ASV_98	0	0	0	0	0	0	0	0	0
ASV_980	0	0	0	0	0	0	0	0	0
ASV_981	0	0	0	0	0	0	0	0	0
ASV_984	0	0	0	0	0	0	0	0	0
ASV_985	0	0	0	0	0	0	0	0	0
ASV_986	0	0	0	0	0	0	0	0	0
ASV_987	0	0	0	0	0	0	0	0	0
ASV_988	0	0	0	0	0	0	0	0	0
ASV_989	0	0	0	0	83	0	0	0	0
ASV_995	0	0	0	0	0	0	0	0	0
ASV_996	0	0	0	0	0	0	0	0	0
ASV_997	0	0	0	0	0	0	0	0	0
ASV_998	0	0	0	0	0	0	0	0	0
ASV_999	0	0	0	0	0	0	0	0	0

Table S3. Taxonomy and putative ecological function for the metabarcoding of the mycobiota of *Ectophylla alba* feces and *Ficus colubrinae* fruits.

ASV	Phylum	Class	Order	Family	Genus	Species	Function
ASV_10	Ascomycota	Laboulbeniomycetes	Pyxidiophorales	Pyxidiophoraceae	Pyxidiophora	arvernensis	fungicolous
ASV_100	Ascomycota	Saccharomycetes	Saccharomycetales	Phaffomycetaceae	Wickerhamomyces	Wickerhamomyces_sp	insect symbiont
ASV_1004	Basidiomycota	Cystobasidiomycetes	unid_Cystobasidiomycetes_order	Symmetrosporaceae	Symmetrospora	Symmetrospora_sp	saprophyte
ASV_1005	Ascomycota	Eurotiomycetes	Eurotiales	Aspergillaceae	Aspergillus	conicus	saprophyte
ASV_1006	Ascomycota	Eurotiomycetes	Chaetothyriales	unid_Chaetothyriales_fam	unid_genus	unid_sp	unknown
ASV_1007	unid_Phylum	unid_class	unid_order	unid_fam	unid_genus	unid_sp	unknown
ASV_1008	Ascomycota	Sordariomycetes	Trichosphaeriales	Trichosphaeriaceae	Nigrospora	Nigrospora_sp	saprophyte
ASV_1009	unid_Phylum	unid_class	unid_order	unid_fam	unid_genus	unid_sp	unknown
ASV_101	Basidiomycota	Agaricomycetes	unid_Agaricomycetes_order	unid_Agaricomycetes_fam	unid_genus	unid_sp	unknown
ASV_1014	unid_Phylum	unid_class	unid_order	unid_fam	unid_genus	unid_sp	unknown
ASV_1015	unid_Phylum	unid_class	unid_order	unid_fam	unid_genus	unid_sp	unknown
ASV_1016	unid_Phylum	unid_class	unid_order	unid_fam	unid_genus	unid_sp	unknown
ASV_1017	Basidiomycota	Agaricomycetes	Cantharellales	Ceratobasidiaceae	Thanatephorus	cucumeris	phytopathogen
ASV_1018	Basidiomycota	Agaricomycetes	Polyporales	Polyporaceae	Trametes	Trametes_sp	saprophyte
ASV_1021	unid_Phylum	unid_class	unid_order	unid_fam	unid_genus	unid_sp	unknown
ASV_1022	Basidiomycota	Agaricomycetes	Polyporales	Phanerochaetaceae	Bjerkandera	adusta	saprophyte
ASV_1023	unid_Phylum	unid_class	unid_order	unid_fam	unid_genus	unid_sp	unknown
ASV_1024	Basidiomycota	Agaricomycetes	unid_Agaricomycetes_order	unid_Agaricomycetes_fam	unid_genus	unid_sp	unknown
ASV_103	Basidiomycota	unid_Basidiomycota_class	unid_Basidiomycota_order	unid_Basidiomycota_fam	unid_genus	unid_sp	unknown
ASV_1030	Ascomycota	Eurotiomycetes	Chaetothyriales	Herpotrichiellaceae	Exophiala	moniliae	animal pathogen
ASV_1031	Ascomycota	Eurotiomycetes	Chaetothyriales	Cyphellophoraceae	Cyphellophora	sessilis	saprophyte
ASV_1032	unid_Phylum	unid_class	unid_order	unid_fam	unid_genus	unid_sp	unknown
ASV_1033	Ascomycota	Orbiliomycetes	Orbiliales	Orbiliaceae	unid_genus	unid_sp	saprophyte
ASV_1034	unid_Phylum	unid_class	unid_order	unid_fam	unid_genus	unid_sp	unknown
ASV_1035	unid_Phylum	unid_class	unid_order	unid_fam	unid_genus	unid_sp	unknown
ASV_1040	Basidiomycota	Agaricomycetes	Cantharellales	unid_Cantharellales_fam	Multiclavula	Multiclavula_sp	lichen
ASV_1041	Basidiomycota	Exobasidiomycetes	Georgefischeriales	Tilletiariaceae	Tilletiaria	anomala	unknown
ASV_1042	unid_Phylum	unid_class	unid_order	unid_fam	unid_genus	unid_sp	unknown
ASV_1045	Basidiomycota	Tremellomycetes	Tremellales	Bulleribasidiaceae	Dioszegia	var. yunspnensis	unknown
ASV_1046	Ascomycota	Sordariomycetes	Hypocreales	unid_Hypocreales_fam	unid_genus	unid_sp	unknown
ASV_1047	unid_Phylum	unid_class	unid_order	unid_fam	unid_genus	unid_sp	unknown
ASV_105	Basidiomycota	Wallemiomycetes	Wallemiales	Wallemiaceae	Wallemia	muriae	animal commensal
ASV_1054	Basidiomycota	Agaricomycetes	Polyporales	Ganodermataceae	Ganoderma	Ganoderma_sp	saprophyte
ASV_1055	Ascomycota	Eurotiomycetes	Chaetothyriales	Trichomeriaceae	unid_genus	unid_sp	unknown
ASV_106	unid_Phylum	unid_class	unid_order	unid_fam	unid_genus	unid_sp	unknown
ASV_1062	Ascomycota	Dothideomycetes	Pleosporales	Didymosphaeriaceae	Montagnula	Montagnula_sp	saprophyte
ASV_1063	Ascomycota	Sordariomycetes	Glomerellales	Glomerellaceae	Colletotrichum	Colletotrichum_sp	phytopathogen
ASV_1064	unid_Phylum	unid_class	unid_order	unid_fam	unid_genus	unid_sp	unknown
ASV_1067	Basidiomycota	Agaricostilbomycetes	Agaricostilbales	Chionosphaeraceae	unid_genus	unid_sp	unknown
ASV_1068	Basidiomycota	Exobasidiomycetes	Exobasidiales	Brachybasidiaceae	Meira	Meira_sp	insecticolous
ASV_1069	Basidiomycota	Agaricomycetes	Hymenochaetales	Schizoporaceae	Xylodon	flaviporus	saprophyte
ASV_107	Basidiomycota	Agaricomycetes	Polyporales	Meruliaceae	Phlebia	tremellosa	saprophyte
ASV_1070	unid_Phylum	unid_class	unid_order	unid_fam	unid_genus	unid_sp	unknown
ASV_1071	Ascomycota	Dothideomycetes	Capnodiales	Teratosphaeriaceae	Hyweljonesia	queenslandica	insecticolous
ASV_1078	Basidiomycota	Agaricomycetes	Polyporales	Ischnodermataceae	Ischnoderma	benzoinum	saprophyte

ASV_1079	Basidiomycota	Agaricomycetes	Polyporales	Ischnodermataceae	Ischnoderma	benzoinum	saprophyte
ASV_108	Ascomycota	Eurotiomycetes	Eurotiales	Aspergillaceae	Aspergillus	glabripes	saprophyte
ASV_1080	unid_Phylum	unid_class	unid_order	unid_fam	unid_genus	unid_sp	unknown
ASV_1081	Ascomycota	Dothideomycetes	Pleosporales	Didymosphaeriaceae	Pseudopithomyces	rosae	phytopathogen
ASV_1082	Ascomycota	Sordariomycetes	Diaporthales	Diaporthaceae	Diaporthe	Diaporthe_sp	phytopathogen
ASV_109	Ascomycota	Saccharomycetes	Saccharomycetales	unid_Saccharomycetales_fam	unid_genus	unid_sp	unknown
ASV_1092	unid_Phylum	unid_class	unid_order	unid_fam	unid_genus	unid_sp	unknown
ASV_1093	Basidiomycota	Agaricomycetes	Trechisporales	unid_Trechisporales_fam	unid_genus	unid_sp	saprophyte
ASV_1094	Ascomycota	Saccharomycetes	Saccharomycetales	Debaryomycetaceae	Debaryomyces	nepalensis	animal commensal
ASV_1100	unid_Phylum	unid_class	unid_order	unid_fam	unid_genus	unid_sp	unknown
ASV_1101	Basidiomycota	Wallemiomycetes	Wallemiales	Wallemiaceae	Wallemia	muriae	animal commensal
ASV_1105	Ascomycota	Eurotiomycetes	Chaetothyriales	Trichomeriaceae	Trichomerium	dioscoreae	epiphyte
ASV_1106	Ascomycota	Dothideomycetes	unid_Dothideomycetes_order	unid_Dothideomycetes_fam	unid_genus	unid_sp	unknown
ASV_1109	Ascomycota	Dothideomycetes	Capnodiales	unid_Capnodiales_fam	unid_genus	unid_sp	saprophyte
ASV_1110	Basidiomycota	unid_Basidiomycota_class	unid_Basidiomycota_order	unid_Basidiomycota_fam	unid_genus	unid_sp	unknown
ASV_1111	Basidiomycota	Malasseziomycetes	Malasseziales	Malasseziaceae	Malassezia	globosa	animal commensal
ASV_1112	unid_Phylum	unid_class	unid_order	unid_fam	unid_genus	unid_sp	unknown
ASV_1113	Ascomycota	Sordariomycetes	Hypocreales	Bionectriaceae	Ijuhya	Ijuhya_sp	saprophyte
ASV_1120	unid_Phylum	unid_class	unid_order	unid_fam	unid_genus	unid_sp	unknown
ASV_1121	Ascomycota	Eurotiomycetes	Eurotiales	Aspergillaceae	Penicillium	jiangxiense	saprophyte
ASV_1122	Basidiomycota	Agaricomycetes	Polyporales	Meruliaceae	Phlebia	tremellosa	saprophyte
ASV_1123	Ascomycota	Sordariomycetes	Hypocreales	unid_Hypocreales_fam	Acremonium	hyalinulum	unknown
ASV_1124	Ascomycota	Eurotiomycetes	Chaetothyriales	Trichomeriaceae	Trichomerium	siamensis	epiphyte
ASV_1125	Ascomycota	Eurotiomycetes	Chaetothyriales	Herpotrichiellaceae	Cladophialophora	Cladophialophora_sp	animal pathogen
ASV_1126	Ascomycota	Sordariomycetes	Hypocreales	Cordycipitaceae	Simplicillium	chinense	fungicolous
ASV_1127	Ascomycota	Dothideomycetes	Capnodiales	Teratosphaeriaceae	Hyweljonesia	queenslandica	insecticolous
ASV_1128	Ascomycota	Dothideomycetes	unid_Dothideomycetes_order	unid_Dothideomycetes_fam	unid_genus	unid_sp	unknown
ASV_1129	Basidiomycota	Tremellomycetes	Cystofilobasidiales	Cystofilobasidiaceae	Cystofilobasidium	capitatum	animal commensal
ASV_1130	Ascomycota	Dothideomycetes	Capnodiales	Mycosphaerellaceae	Chaetothyrina	guttulata	saprophyte
ASV_1136	Basidiomycota	Agaricomycetes	Polyporales	Meripilaceae	Rigidoporus	ulmarius	saprophyte
ASV_1137	Ascomycota	Sordariomycetes	Hypocreales	Bionectriaceae	unid_genus	unid_sp	unknown
ASV_1138	unid_Phylum	unid_class	unid_order	unid_fam	unid_genus	unid_sp	unknown
ASV_1139	Basidiomycota	Malasseziomycetes	Malasseziales	Malasseziaceae	Malassezia	arunalokei	animal commensal
ASV_114	Ascomycota	Sordariomycetes	Hypocreales	Nectriaceae	Albonectria	rigidiuscula	saprophyte
ASV_1142	Basidiomycota	Pucciniomycetes	Septobasidiales	Septobasidiaceae	unid_genus	unid_sp	unknown
ASV_1143	Basidiomycota	Agaricomycetes	Agaricales	Psathyrellaceae	Psathyrella	candolleasp	saprophyte
ASV_1144	Ascomycota	Sordariomycetes	Hypocreales	Cordycipitaceae	Simplicillium	Simplicillium_sp	fungicolous
ASV_1145	Basidiomycota	Agaricomycetes	Sebacinales	Sebacinaceae	Chaetospermum	camelliae	saprophyte
ASV_115	Basidiomycota	Cystobasidiomycetes	Cystobasidiales	Cystobasidiaceae	Occultifur	Occultifur_sp	fungicolous
ASV_1151	Basidiomycota	Agaricomycetes	unid_Agaricomycetes_order	unid_Agaricomycetes_fam	unid_genus	unid_sp	unknown
ASV_1156	Ascomycota	Sordariomycetes	Meliolales	Meliolaceae	Irenopsis	walsurae	phytopathogen
ASV_1157	Ascomycota	Dothideomycetes	Capnodiales	Cladosporiaceae	Cladosporium	Cladosporium_sp	saprophyte
ASV_1158	unid_Phylum	unid_class	unid_order	unid_fam	unid_genus	unid_sp	unknown
ASV_1159	Basidiomycota	Agaricomycetes	Polyporales	Steccherinaceae	Nigroporus	vinosus	saprophyte
ASV_1160	Ascomycota	Dothideomycetes	Pleosporales	Cucurbitariaceae	Pyrenochaetopsis	leptospora	phytopathogen
ASV_1161	Ascomycota	Orbiliomycetes	Orbiliales	unid_Orbiliales_fam	Lecophagus	longisporus	unknown
ASV_1167	Ascomycota	Dothideomycetes	unid_Dothideomycetes_order	unid_Dothideomycetes_fam	unid_genus	unid_sp	unknown
ASV_1168	Ascomycota	Eurotiomycetes	Eurotiales	Aspergillaceae	Aspergillus	Aspergillus_sp	saprophyte
ASV_1169	Ascomycota	Dothideomycetes	Pleosporales	Thyridariaceae	unid_genus	unid_sp	unknown
ASV_117	unid_Phylum	unid_class	unid_order	unid_fam	unid_genus	unid_sp	unknown

ASV_1170	unid_Phylum	unid_class	unid_order	unid_fam	unid_genus	unid_sp	unknown
ASV_1171	Ascomycota	Saccharomycetes	Saccharomycetales	Debaryomycetaceae	Kurtzmaniella	Kurtzmaniella_sp	animal commensal
ASV_1172	Basidiomycota	unid_Basidiomycota_class	unid_Basidiomycota_order	unid_Basidiomycota_fam	unid_genus	unid_sp	unknown
ASV_1181	Ascomycota	Dothideomycetes	Pleosporales	Halotthiaceae	Sulcosporium	Sulcosporium_sp	phytopathogen
ASV_1184	Basidiomycota	Agaricomycetes	Agaricales	Typhulaceae	Typhula	micans	saprophyte
ASV_1185	Ascomycota	unid_Ascomycota_class	unid_Ascomycota_order	unid_Ascomycota_fam	unid_genus	unid_sp	unknown
ASV_1186	Basidiomycota	Agaricomycetes	Cantharellales	Ceratobasidiaceae	Ceratobasidium	Ceratobasidium_sp	saprophyte
ASV_1187	Basidiomycota	Agaricomycetes	Polyporales	Polyporaceae	Trametes	cubensis	saprophyte
ASV_1188	Ascomycota	Saccharomycetes	Saccharomycetales	Phaffomycetaceae	Wickerhamomyces	Wickerhamomyces_sp	insect symbiont
ASV_1189	unid_Phylum	unid_class	unid_order	unid_fam	unid_genus	unid_sp	unknown
ASV_1194	unid_Phylum	unid_class	unid_order	unid_fam	unid_genus	unid_sp	unknown
ASV_1195	unid_Phylum	unid_class	unid_order	unid_fam	unid_genus	unid_sp	unknown
ASV_1196	Basidiomycota	Agaricomycetes	Cantharellales	Ceratobasidiaceae	Thanatephorus	cucumeris	phytopathogen
ASV_1197	Ascomycota	Dothideomycetes	Venturiales	Sympoventuriaceae	Ochroconis	cordanae	phytopathogen
ASV_120	Ascomycota	Dothideomycetes	Pleosporales	unid_Pleosporales_fam	unid_genus	unid_sp	unknown
ASV_1204	Ascomycota	Eurotiomycetes	Chaetothyriales	Chaetothyriaceae	unid_genus	unid_sp	saprophyte
ASV_1205	unid_Phylum	unid_class	unid_order	unid_fam	unid_genus	unid_sp	unknown
ASV_1206	Basidiomycota	Malasseziomycetes	Malasseziales	Malasseziaceae	Malassezia	globosa	animal commensal
ASV_1207	Basidiomycota	Agaricomycetes	Agaricales	Psathyrellaceae	Coprinopsis	musae	saprophyte
ASV_1208	Basidiomycota	Tremellomycetes	Trichosporonales	Trichosporonaceae	Cutaneotrichosporon	jirovecii	saprophyte
ASV_1209	unid_Phylum	unid_class	unid_order	unid_fam	unid_genus	unid_sp	unknown
ASV_1210	Basidiomycota	Agaricomycetes	Agaricales	Bolbitiaceae	Panaeolus	papiliospceus	saprophyte
ASV_1211	unid_Phylum	unid_class	unid_order	unid_fam	unid_genus	unid_sp	unknown
ASV_1219	Basidiomycota	Agaricomycetes	Agaricales	Tricholomataceae	Panellus	stipticus	saprophyte
ASV_1220	unid_Phylum	unid_class	unid_order	unid_fam	unid_genus	unid_sp	unknown
ASV_1221	Basidiomycota	Agaricomycetes	Polyporales	Polyporaceae	Trametes	sanguinea	saprophyte
ASV_1222	Basidiomycota	Malasseziomycetes	Malasseziales	Malasseziaceae	Malassezia	restricta	animal commensal
ASV_1223	unid_Phylum	unid_class	unid_order	unid_fam	unid_genus	unid_sp	unknown
ASV_1224	unid_Phylum	unid_class	unid_order	unid_fam	unid_genus	unid_sp	unknown
ASV_1225	Ascomycota	Eurotiomycetes	Chaetothyriales	unid_Chaetothyriales_fam	unid_genus	unid_sp	unknown
ASV_1226	unid_Phylum	unid_class	unid_order	unid_fam	unid_genus	unid_sp	unknown
ASV_1227	Ascomycota	Eurotiomycetes	Chaetothyriales	unid_Chaetothyriales_fam	unid_genus	unid_sp	unknown
ASV_1228	Ascomycota	Dothideomycetes	Pleosporales	unid_Pleosporales_fam	unid_genus	unid_sp	unknown
ASV_1229	unid_Phylum	unid_class	unid_order	unid_fam	unid_genus	unid_sp	unknown
ASV_123	unid_Phylum	unid_class	unid_order	unid_fam	unid_genus	unid_sp	unknown
ASV_1235	Ascomycota	Dothideomycetes	Pleosporales	Didymosphaeriaceae	Paraphaeosphaeria	arecacearum	phytopathogen
ASV_1236	unid_Phylum	unid_class	unid_order	unid_fam	unid_genus	unid_sp	unknown
ASV_1237	unid_Phylum	unid_class	unid_order	unid_fam	unid_genus	unid_sp	unknown
ASV_1238	unid_Phylum	unid_class	unid_order	unid_fam	unid_genus	unid_sp	unknown
ASV_1242	Basidiomycota	Agaricomycetes	Agaricales	Tricholomataceae	Panellus	stipticus	saprophyte
ASV_1243	Basidiomycota	Agaricomycetes	Agaricales	Psathyrellaceae	Coprinopsis	friesii	saprophyte
ASV_1244	Basidiomycota	Exobasidiomycetes	Georgefischeriales	unid_Georgefischeriales_fam	unid_genus	unid_sp	unknown
ASV_1245	unid_Phylum	unid_class	unid_order	unid_fam	unid_genus	unid_sp	unknown
ASV_1246	Ascomycota	Sordariomycetes	Hypocreales	Bionectriaceae	Ijuhya	Ijuhya_sp	saprophyte
ASV_125	Basidiomycota	Malasseziomycetes	Malasseziales	Malasseziaceae	Malassezia	globosa	animal commensal
ASV_1254	Ascomycota	Sordariomycetes	Xylariales	unid_Xylariales_fam	unid_genus	unid_sp	unknown
ASV_1255	Ascomycota	Dothideomycetes	Capnodiales	Cladosporiaceae	Cladosporium	ramotenellum	saprophyte
ASV_1256	Ascomycota	Sordariomycetes	Trichosphaeriales	Trichosphaeriaceae	Nigrospora	oryzae	saprophyte
ASV_1257	Basidiomycota	Tremellomycetes	Cystofilobasidiales	Cystofilobasidiaceae	Cystofilobasidium	capitatum	animal commensal
ASV_1258	Basidiomycota	Agaricomycetes	Polyporales	Ischnodermataceae	Ischnoderma	benzoinum	saprophyte

ASV_1259	Ascomycota	Eurotiomycetes	Chaetothyriales	unid_Chaetothyriales_fam	Neostrelitziana	acaciigena	unknown
ASV_126	Ascomycota	Sordariomycetes	Xylariales	unid_Xylariales_fam	Asteromella	Asteromella_sp	saprophyte
ASV_1267	Ascomycota	Sordariomycetes	Togniniales	Togniniaceae	Phaeoacremonium	Phaeoacremonium_sp	phytopathogen
ASV_1268	Basidiomycota	Wallemiomycetes	Wallemiales	Wallemiaceae	Wallemia	muriae	animal commensal
ASV_1269	unid_Phylum	unid_class	unid_order	unid_fam	unid_genus	unid_sp	unknown
ASV_127	Ascomycota	Sordariomycetes	Hypocreales	Nectriaceae	Fusarium	concentricum	phytopathogen
ASV_1270	unid_Phylum	unid_class	unid_order	unid_fam	unid_genus	unid_sp	unknown
ASV_1271	unid_Phylum	unid_class	unid_order	unid_fam	unid_genus	unid_sp	unknown
ASV_1272	unid_Phylum	unid_class	unid_order	unid_fam	unid_genus	unid_sp	unknown
ASV_1273	Basidiomycota	Agaricomycetes	unid_Agaricomycetes_order	unid_Agaricomycetes_fam	unid_genus	unid_sp	unknown
ASV_1274	Ascomycota	Saccharomycetes	Saccharomycetales	Saccharomycetaceae	Saccharomyces	Saccharomyces_sp	animal commensal
ASV_1275	Ascomycota	Dothideomycetes	Tubeufiales	Tubeufiaceae	unid_genus	unid_sp	saprophyte
ASV_1282	Ascomycota	Sordariomycetes	Coniochaetales	Coniochaetaceae	Coniochaeta	boothii	phytopathogen
ASV_1283	Ascomycota	Lecanoromycetes	Lecanorales	Ramalinaceae	Bacidia	neosquamulosa	lichen
ASV_1284	Ascomycota	Eurotiomycetes	Eurotiales	Aspergillaceae	Penicillium	Penicillium_sp	saprophyte
ASV_1285	Basidiomycota	Malasseziomycetes	Malasseziales	Malasseziaceae	Malassezia	arunalokei	animal commensal
ASV_1289	Ascomycota	Dothideomycetes	Tubeufiales	Tubeufiaceae	unid_genus	unid_sp	saprophyte
ASV_1296	Basidiomycota	Agaricomycetes	Agaricales	Psathyrellaceae	Psathyrella	Psathyrella_sp	saprophyte
ASV_1297	Basidiomycota	Agaricomycetes	Polyporales	Ganodermataceae	Perenniporia	vanhullii	saprophyte
ASV_1298	Basidiomycota	Tremellomycetes	Tremellales	Rhynchogastremataceae	Papiliotrema	Papiliotrema_sp	epiphyte
ASV_1299	unid_Phylum	unid_class	unid_order	unid_fam	unid_genus	unid_sp	unknown
ASV_13	unid_Phylum	unid_class	unid_order	unid_fam	unid_genus	unid_sp	unknown
ASV_130	Basidiomycota	unid_Basidiomycota_class	unid_Basidiomycota_order	unid_Basidiomycota_fam	unid_genus	unid_sp	unknown
ASV_1300	unid_Phylum	unid_class	unid_order	unid_fam	unid_genus	unid_sp	unknown
ASV_1301	unid_Phylum	unid_class	unid_order	unid_fam	unid_genus	unid_sp	unknown
ASV_1302	Ascomycota	Sordariomycetes	Diaporthales	unid_Diaporthales_fam	Leptosillia	acerina	phytopathogen
ASV_1303	Ascomycota	Dothideomycetes	Pleosporales	Didymosphaeriaceae	Pseudopithomyces	rosae	phytopathogen
ASV_1304	Ascomycota	Eurotiomycetes	Chaetothyriales	Herpotrichiellaceae	Exophiala	brunnea	animal pathogen
ASV_1312	Basidiomycota	Agaricomycetes	Agaricales	Agaricaceae	Lepiota	Lepiota_sp	saprophyte
ASV_1313	Ascomycota	Dothideomycetes	unid_Dothideomycetes_order	unid_Dothideomycetes_fam	unid_genus	unid_sp	unknown
ASV_1314	unid_Phylum	unid_class	unid_order	unid_fam	unid_genus	unid_sp	unknown
ASV_1325	unid_Phylum	unid_class	unid_order	unid_fam	unid_genus	unid_sp	unknown
ASV_133	Ascomycota	Saccharomycetes	Saccharomycetales	Phaffomycetaceae	Wickerhamomyces	Wickerhamomyces_sp	insect symbiont
ASV_1331	Basidiomycota	Agaricomycetes	Russulales	unid_Russulales_fam	unid_genus	unid_sp	unknown
ASV_1332	unid_Phylum	unid_class	unid_order	unid_fam	unid_genus	unid_sp	unknown
ASV_1333	Basidiomycota	Agaricomycetes	Cantharellales	Ceratobasidiaceae	Thanatephorus	cucumeris	phytopathogen
ASV_1334	Basidiomycota	Agaricomycetes	Agaricales	Tricholomataceae	Merismodes	fasciculata	saprophyte
ASV_134	unid_Phylum	unid_class	unid_order	unid_fam	unid_genus	unid_sp	unknown
ASV_1341	unid_Phylum	unid_class	unid_order	unid_fam	unid_genus	unid_sp	unknown
ASV_1342	unid_Phylum	unid_class	unid_order	unid_fam	unid_genus	unid_sp	unknown
ASV_1343	unid_Phylum	unid_class	unid_order	unid_fam	unid_genus	unid_sp	unknown
ASV_1344	Ascomycota	Dothideomycetes	Pleosporales	Morosphaeriaceae	Acrocalymma	Acrocalymma_sp	phytopathogen
ASV_1345	unid_Phylum	unid_class	unid_order	unid_fam	unid_genus	unid_sp	unknown
ASV_1346	Ascomycota	Dothideomycetes	unid_Dothideomycetes_order	unid_Dothideomycetes_fam	unid_genus	unid_sp	unknown
ASV_135	Basidiomycota	Agaricomycetes	Polyporales	Meruliaceae	Phlebia	tremellosa	saprophyte
ASV_1351	Ascomycota	Sordariomycetes	Xylariales	unid_Xylariales_fam	unid_genus	unid_sp	unknown
ASV_1352	Ascomycota	Lecanoromycetes	Lecanorales	Ramalinaceae	Bacidia	neosquamulosa	lichen
ASV_1353	unid_Phylum	unid_class	unid_order	unid_fam	unid_genus	unid_sp	unknown
ASV_1354	Ascomycota	Sordariomycetes	Trichosphaeriales	Trichosphaeriaceae	Nigrospora	Nigrospora_sp	saprophyte
ASV_1355	unid_Phylum	unid_class	unid_order	unid_fam	unid_genus	unid_sp	unknown

ASV_1356	Ascomycota	Dothideomycetes	Botryosphaeriales	Phyllostictaceae	Phyllosticta	capitalensis	phytopathogen
ASV_1357	Ascomycota	unid_Ascomycota_class	unid_Ascomycota_order	unid_Ascomycota_fam	unid_genus	unid_sp	unknown
ASV_1358	Ascomycota	Eurotiomycetes	Phaeomoniellales	Phaeomoniellaceae	Phaeomoniella	Phaeomoniella_sp	phytopathogen
ASV_1359	Basidiomycota	unid_Basidiomycota_class	unid_Basidiomycota_order	unid_Basidiomycota_fam	unid_genus	unid_sp	unknown
ASV_1366	Basidiomycota	Agaricomycetes	Agaricales	Tricholomataceae	Panellus	stipticus	saprophyte
ASV_1367	unid_Phylum	unid_class	unid_order	unid_fam	unid_genus	unid_sp	unknown
ASV_1368	Ascomycota	Sordariomycetes	Xylariales	unid_Xylariales_fam	unid_genus	unid_sp	unknown
ASV_1369	Basidiomycota	Agaricostilbomycetes	Agaricostilbales	Chionosphaeraceae	Cystobasidiopsis	Cystobasidiopsis_sp	unknown
ASV_137	Basidiomycota	Wallemiomycetes	Wallemiales	Wallemiaceae	Wallemia	canadensis	animal commensal
ASV_1373	Ascomycota	Sordariomycetes	Hypocreales	Nectriaceae	Fusarium	polyphialidicum	phytopathogen
ASV_1374	Ascomycota	Sordariomycetes	Hypocreales	unid_Hypocreales_fam	Acremonium	polychromum	unknown
ASV_1375	unid_Phylum	unid_class	unid_order	unid_fam	unid_genus	unid_sp	unknown
ASV_138	Basidiomycota	Exobasidiomycetes	Exobasidiales	Brachybasidiaceae	Meira	Meira_sp	insecticolous
ASV_139	unid_Phylum	unid_class	unid_order	unid_fam	unid_genus	unid_sp	unknown
ASV_1390	unid_Phylum	unid_class	unid_order	unid_fam	unid_genus	unid_sp	unknown
ASV_1391	unid_Phylum	unid_class	unid_order	unid_fam	unid_genus	unid_sp	unknown
ASV_1392	unid_Phylum	unid_class	unid_order	unid_fam	unid_genus	unid_sp	unknown
ASV_140	Basidiomycota	Agaricomycetes	Cantharellales	Ceratobasidiaceae	Thanatephorus	cucumeris	phytopathogen
ASV_1400	Ascomycota	Sordariomycetes	Glomerellales	Reticulascaceae	Pseudoacrodictys	Pseudoacrodictys_sp	phytopathogen
ASV_1401	unid_Phylum	unid_class	unid_order	unid_fam	unid_genus	unid_sp	unknown
ASV_1402	Ascomycota	Sordariomycetes	Xylariales	Sporocadaceae	Pseudopestalotiopsis	simitheae	phytopathogen
ASV_1403	unid_Phylum	unid_class	unid_order	unid_fam	unid_genus	unid_sp	unknown
ASV_1404	unid_Phylum	unid_class	unid_order	unid_fam	unid_genus	unid_sp	unknown
ASV_1405	unid_Phylum	unid_class	unid_order	unid_fam	unid_genus	unid_sp	unknown
ASV_1412	Ascomycota	unid_Ascomycota_class	unid_Ascomycota_order	unid_Ascomycota_fam	unid_genus	unid_sp	unknown
ASV_1413	unid_Phylum	unid_class	unid_order	unid_fam	unid_genus	unid_sp	unknown
ASV_1414	Ascomycota	Sordariomycetes	Xylariales	unid_Xylariales_fam	unid_genus	unid_sp	unknown
ASV_1415	Ascomycota	Dothideomycetes	Capnodiales	Mycosphaerellaceae	Pseudocercospora	norchiensis	phytopathogen
ASV_1422	Ascomycota	Eurotiomycetes	Eurotiales	Aspergillaceae	Penicillium	Penicillium_sp	saprophyte
ASV_1423	Ascomycota	Dothideomycetes	Pleosporales	Coniothyriaceae	Coniothyrium	Coniothyrium_sp	phytopathogen
ASV_1424	unid_Phylum	unid_class	unid_order	unid_fam	unid_genus	unid_sp	unknown
ASV_1425	Basidiomycota	Agaricomycetes	Cantharellales	unid_Cantharellales_fam	Multiclavula	Multiclavula_sp	lichen
ASV_1426	Basidiomycota	Malasseziomycetes	Malasseziales	Malasseziaceae	Malassezia	restricta	animal commensal
ASV_1427	Ascomycota	Sordariomycetes	Glomerellales	Glomerellaceae	Colletotrichum	Colletotrichum_sp	phytopathogen
ASV_1428	Ascomycota	Sordariomycetes	Xylariales	Apiosporaceae	unid_genus	unid_sp	unknown
ASV_1436	Ascomycota	Sordariomycetes	Hypocreales	Nectriaceae	Xenoacremonium	falcatum	saprophyte
ASV_1437	Ascomycota	unid_Ascomycota_class	unid_Ascomycota_order	unid_Ascomycota_fam	unid_genus	unid_sp	unknown
ASV_1438	Ascomycota	Sordariomycetes	Hypocreales	unid_Hypocreales_fam	unid_genus	unid_sp	unknown
ASV_1439	Ascomycota	Saccharomycetes	Saccharomycetales	Debaryomycetaceae	Debaryomyces	hansenii	animal commensal
ASV_1440	Basidiomycota	Agaricomycetes	Polyporales	Fomitopsidaceae	unid_genus	unid_sp	saprophyte
ASV_1441	Ascomycota	Sordariomycetes	Xylariales	Sporocadaceae	Pseudopestalotiopsis	simitheae	phytopathogen
ASV_1442	unid_Phylum	unid_class	unid_order	unid_fam	unid_genus	unid_sp	unknown
ASV_145	unid_Phylum	unid_class	unid_order	unid_fam	unid_genus	unid_sp	unknown
ASV_1459	Basidiomycota	Agaricomycetes	Agaricales	Tricholomataceae	Panellus	stipticus	saprophyte
ASV_1460	Ascomycota	Sordariomycetes	Hypocreales	Nectriaceae	Fusarium	pseudensiforme	saprophyte
ASV_1461	Ascomycota	Sordariomycetes	Xylariales	Xylariaceae	Nemania	abortiva	saprophyte
ASV_1462	Ascomycota	Eurotiomycetes	Chaetothyriales	Trichomeriaceae	Trichomerium	siamensis	epiphyte
ASV_1463	Basidiomycota	Cystobasidiomycetes	unid_Cystobasidiomycetes_order	Symmetrosporaceae	Symmetrospora	Symmetrospora_sp	saprophyte
ASV_1464	Ascomycota	Eurotiomycetes	unid_Eurotiomycetes_order	unid_Eurotiomycetes_fam	unid_genus	unid_sp	unknown
ASV_147	unid_Phylum	unid_class	unid_order	unid_fam	unid_genus	unid_sp	unknown

ASV_1472	Ascomycota	Eurotiomycetes	Chaetothyriales	unid_Chaetothyriales_fam	unid_genus	unid_sp	unknown
ASV_1473	Ascomycota	Eurotiomycetes	Eurotiales	Aspergillaceae	Aspergillus	conicus	saprophyte
ASV_1474	unid_Phylum	unid_class	unid_order	unid_fam	unid_genus	unid_sp	unknown
ASV_1475	Basidiomycota	Agaricomycetes	Corticiales	Corticiaceae	Limonomyces	roseipellis	phytopathogen
ASV_1483	unid_Phylum	unid_class	unid_order	unid_fam	unid_genus	unid_sp	unknown
ASV_1484	unid_Phylum	unid_class	unid_order	unid_fam	unid_genus	unid_sp	unknown
ASV_1485	Basidiomycota	Agaricomycetes	Sebacinales	unid_Sebacinales_fam	unid_genus	unid_sp	unknown
ASV_1486	Ascomycota	Eurotiomycetes	Chaetothyriales	Trichomeriaceae	Trichomerium	dioscoreae	epiphyte
ASV_1487	Basidiomycota	Agaricomycetes	unid_Agaricomycetes_order	unid_Agaricomycetes_fam	unid_genus	unid_sp	unknown
ASV_1488	Basidiomycota	Agaricomycetes	Polyporales	Polyporaceae	Grammothele	fuligo	saprophyte
ASV_1489	Ascomycota	Dothideomycetes	Pleosporales	Thyridariaceae	Neoroussoella	Neoroussoella_sp	saprophyte
ASV_149	Basidiomycota	Wallemiomycetes	Wallemiales	Wallemiaceae	Wallemia	canadensis	animal commensal
ASV_1490	Ascomycota	Dothideomycetes	Pleosporales	unid_Pleosporales_fam	Hermatomyces	tectonae	saprophyte
ASV_150	Basidiomycota	Malasseziomycetes	Malasseziales	Malasseziaceae	Malassezia	globosa	animal commensal
ASV_1500	Ascomycota	Dothideomycetes	Pleosporales	Lophiotremataceae	unid_genus	unid_sp	unknown
ASV_1501	Ascomycota	Eurotiomycetes	Chaetothyriales	Cyphellophoraceae	Cyphellophora	eucalypti	saprophyte
ASV_1502	unid_Phylum	unid_class	unid_order	unid_fam	unid_genus	unid_sp	unknown
ASV_1503	Basidiomycota	Pucciniomycetes	Septobasidiales	Septobasidiaceae	Septobasidium	Septobasidium_sp	insecticolous
ASV_1504	Ascomycota	Eurotiomycetes	Chaetothyriales	Trichomeriaceae	unid_genus	unid_sp	unknown
ASV_151	Basidiomycota	Wallemiomycetes	Wallemiales	Wallemiaceae	Wallemia	canadensis	animal commensal
ASV_1515	Ascomycota	Dothideomycetes	Pleosporales	Didymosphaeriaceae	unid_genus	unid_sp	phytopathogen
ASV_1516	Ascomycota	Sordariomycetes	Xylariales	Xylariaceae	Hypoxylon	monticulosum	saprophyte
ASV_1517	unid_Phylum	unid_class	unid_order	unid_fam	unid_genus	unid_sp	unknown
ASV_1518	unid_Phylum	unid_class	unid_order	unid_fam	unid_genus	unid_sp	unknown
ASV_1519	unid_Phylum	unid_class	unid_order	unid_fam	unid_genus	unid_sp	unknown
ASV_152	unid_Phylum	unid_class	unid_order	unid_fam	unid_genus	unid_sp	unknown
ASV_1520	Basidiomycota	Agaricomycetes	Agaricales	Psathyrellaceae	Coprinellus	dissemisptus	saprophyte
ASV_1530	Basidiomycota	Agaricomycetes	Cantharellales	unid_Cantharellales_fam	Sistotrema	sernanderi	saprophyte
ASV_1531	Basidiomycota	Pucciniomycetes	Septobasidiales	Septobasidiaceae	unid_genus	unid_sp	unknown
ASV_1532	unid_Phylum	unid_class	unid_order	unid_fam	unid_genus	unid_sp	unknown
ASV_1533	Ascomycota	Eurotiomycetes	Chaetothyriales	Cyphellophoraceae	Cyphellophora	eucalypti	saprophyte
ASV_1534	unid_Phylum	unid_class	unid_order	unid_fam	unid_genus	unid_sp	unknown
ASV_1535	unid_Phylum	unid_class	unid_order	unid_fam	unid_genus	unid_sp	unknown
ASV_1536	Ascomycota	Sordariomycetes	Diaporthales	Diaporthaceae	Diaporthe	tulliensis	phytopathogen
ASV_1537	Ascomycota	Sordariomycetes	Hypocreales	Bionectriaceae	unid_genus	unid_sp	unknown
ASV_154	Basidiomycota	Agaricomycetes	Cantharellales	unid_Cantharellales_fam	Multiclavula	Multiclavula_sp	lichen
ASV_1549	unid_Phylum	unid_class	unid_order	unid_fam	unid_genus	unid_sp	unknown
ASV_1550	Ascomycota	Eurotiomycetes	Chaetothyriales	Chaetothyriaceae	Ceramothyrium	melastoma	saprophyte
ASV_1551	Ascomycota	unid_Ascomycota_class	unid_Ascomycota_order	unid_Ascomycota_fam	unid_genus	unid_sp	unknown
ASV_1552	Ascomycota	Dothideomycetes	Capnodiales	Neodevriesiaceae	Neodevriesia	shakazului	saprophyte
ASV_1553	unid_Phylum	unid_class	unid_order	unid_fam	unid_genus	unid_sp	unknown
ASV_1554	Ascomycota	Eurotiomycetes	Eurotiales	Trichocomaceae	Talaromyces	wortmannii	saprophyte
ASV_1555	Basidiomycota	Agaricomycetes	Polyporales	Meruliaceae	Gyrophanopsis	Gyrophanopsis_sp	saprophyte
ASV_1556	unid_Phylum	unid_class	unid_order	unid_fam	unid_genus	unid_sp	unknown
ASV_1557	Ascomycota	Dothideomycetes	unid_Dothideomycetes_order	unid_Dothideomycetes_fam	unid_genus	unid_sp	unknown
ASV_1558	unid_Phylum	unid_class	unid_order	unid_fam	unid_genus	unid_sp	unknown
ASV_1559	unid_Phylum	unid_class	unid_order	unid_fam	unid_genus	unid_sp	unknown
ASV_1560	unid_Phylum	unid_class	unid_order	unid_fam	unid_genus	unid_sp	unknown
ASV_1561	Basidiomycota	Tremellomycetes	Cystofilobasidiales	Cystofilobasidiaceae	Cystofilobasidium	capitatum	animal commensal
ASV_1562	unid_Phylum	unid_class	unid_order	unid_fam	unid_genus	unid_sp	unknown

Table S3

ASV_1563	Basidiomycota	Wallemiomycetes	Wallemiales	Wallemiaceae	Wallemia	canadensis	animal commensal
ASV_157	Ascomycota	Sordariomycetes	Glomerellales	Glomerellaceae	Colletotrichum	Colletotrichum_sp	phytopathogen
ASV_1578	Basidiomycota	Tremellomycetes	Tremellales	Trimorphomycetaceae	Carlosrosaea	Carlosrosaea_sp	epiphyte
ASV_1579	Ascomycota	unid_Ascomycota_class	unid_Ascomycota_order	unid_Ascomycota_fam	unid_genus	unid_sp	unknown
ASV_1580	Ascomycota	Dothideomycetes	Capnodiales	Mycosphaerellaceae	unid_genus	unid_sp	phytopathogen
ASV_1581	unid_Phylum	unid_class	unid_order	unid_fam	unid_genus	unid_sp	unknown
ASV_1582	Ascomycota	Dothideomycetes	Pleosporales	unid_Pleosporales_fam	unid_genus	unid_sp	unknown
ASV_1583	Ascomycota	Dothideomycetes	Pleosporales	Periconiaceae	Periconia	byssoides	saprophyte
ASV_1584	Basidiomycota	Agaricomycetes	Polyporales	Ganodermataceae	Ganoderma	ecuadoriense	saprophyte
ASV_1585	Ascomycota	Eurotiomycetes	Chaetothyriales	unid_Chaetothyriales_fam	Strelitziana	Strelitziana_sp	unknown
ASV_1586	Ascomycota	Sordariomycetes	Myrmecridiales	unid_Myrmecridiales_fam	unid_genus	unid_sp	unknown
ASV_1587	unid_Phylum	unid_class	unid_order	unid_fam	unid_genus	unid_sp	unknown
ASV_1588	Ascomycota	Dothideomycetes	Pleosporales	Didymosphaeriaceae	Spegazzinia	lobulata	phytopathogen
ASV_159	Basidiomycota	Cystobasidiomycetes	unid_Cystobasidiomycetes_order	Symmetrosporaceae	Symmetrospora	Symmetrospora_sp	saprophyte
ASV_1602	Basidiomycota	Malasseziomycetes	Malasseziales	Malasseziaceae	Malassezia	arunalokei	animal commensal
ASV_1603	unid_Phylum	unid_class	unid_order	unid_fam	unid_genus	unid_sp	unknown
ASV_1604	unid_Phylum	unid_class	unid_order	unid_fam	unid_genus	unid_sp	unknown
ASV_1605	Ascomycota	Sordariomycetes	Glomerellales	Glomerellaceae	Colletotrichum	ignotum	phytopathogen
ASV_1606	Basidiomycota	Malasseziomycetes	Malasseziales	Malasseziaceae	Malassezia	restricta	animal commensal
ASV_1607	unid_Phylum	unid_class	unid_order	unid_fam	unid_genus	unid_sp	unknown
ASV_1608	unid_Phylum	unid_class	unid_order	unid_fam	unid_genus	unid_sp	unknown
ASV_1609	Ascomycota	Dothideomycetes	Dothideales	Aureobasidiaceae	Aureobasidium	Aureobasidium_sp	saprophyte
ASV_161	Basidiomycota	Agaricomycetes	Sebacinales	unid_Sebacinales_fam	unid_genus	unid_sp	unknown
ASV_1610	unid_Phylum	unid_class	unid_order	unid_fam	unid_genus	unid_sp	unknown
ASV_1611	unid_Phylum	unid_class	unid_order	unid_fam	unid_genus	unid_sp	unknown
ASV_1612	Basidiomycota	Agaricomycetes	unid_Agaricomycetes_order	unid_Agaricomycetes_fam	unid_genus	unid_sp	unknown
ASV_1613	unid_Phylum	unid_class	unid_order	unid_fam	unid_genus	unid_sp	unknown
ASV_1614	Basidiomycota	Pucciniomycetes	Septobasidiales	Septobasidiaceae	unid_genus	unid_sp	unknown
ASV_162	unid_Phylum	unid_class	unid_order	unid_fam	unid_genus	unid_sp	unknown
ASV_1626	Basidiomycota	Agaricomycetes	Polyporales	Fomitopsidaceae	Amyloporia	sinuosa	saprophyte
ASV_1627	unid_Phylum	unid_class	unid_order	unid_fam	unid_genus	unid_sp	unknown
ASV_1628	Basidiomycota	Agaricomycetes	unid_Agaricomycetes_order	unid_Agaricomycetes_fam	unid_genus	unid_sp	unknown
ASV_1629	Ascomycota	Eurotiomycetes	unid_Eurotiomycetes_order	unid_Eurotiomycetes_fam	unid_genus	unid_sp	unknown
ASV_163	Ascomycota	Dothideomycetes	Pleosporales	Morosphaeriaceae	Acrocalymma	Acrocalymma_sp	phytopathogen
ASV_1630	unid_Phylum	unid_class	unid_order	unid_fam	unid_genus	unid_sp	unknown
ASV_1636	unid_Phylum	unid_class	unid_order	unid_fam	unid_genus	unid_sp	unknown
ASV_1637	unid_Phylum	unid_class	unid_order	unid_fam	unid_genus	unid_sp	unknown
ASV_1638	unid_Phylum	unid_class	unid_order	unid_fam	unid_genus	unid_sp	unknown
ASV_1639	Ascomycota	Sordariomycetes	Trichosphaeriales	Trichosphaeriaceae	Nigrospora	Nigrospora_sp	saprophyte
ASV_1640	Ascomycota	Saccharomycetes	Saccharomycetales	Phaffomycetaceae	Wickerhamomyces	Wickerhamomyces_sp	insect symbiont
ASV_1641	unid_Phylum	unid_class	unid_order	unid_fam	unid_genus	unid_sp	unknown
ASV_1642	Ascomycota	unid_Ascomycota_class	unid_Ascomycota_order	unid_Ascomycota_fam	Tricladiella	pluvialis	saprophyte
ASV_1643	Ascomycota	Dothideomycetes	Pleosporales	Thyridariaceae	unid_genus	unid_sp	unknown
ASV_1644	Basidiomycota	Agaricomycetes	Agaricales	Strophariaceae	Deconica	hartii	saprophyte
ASV_1645	Basidiomycota	Agaricomycetes	Auriculariales	Auriculariaceae	Auricularia	orientalis	saprophyte
ASV_1646	unid_Phylum	unid_class	unid_order	unid_fam	unid_genus	unid_sp	unknown
ASV_1647	Ascomycota	unid_Ascomycota_class	unid_Ascomycota_order	unid_Ascomycota_fam	unid_genus	unid_sp	unknown
ASV_1648	Ascomycota	Sordariomycetes	Diaporthales	unid_Diaporthales_fam	unid_genus	unid_sp	phytopathogen
ASV_1649	Ascomycota	Eurotiomycetes	Eurotiales	Aspergillaceae	Aspergillus	glabripes	saprophyte
ASV_166	Basidiomycota	Wallemiomycetes	Wallemiales	Wallemiaceae	Wallemia	sebi	animal commensal

ASV_1661	Ascomycota	Dothideomycetes	Pleosporales	unid_Pleosporales_fam	unid_genus	unid_sp	unknown
ASV_1662	unid_Phylum	unid_class	unid_order	unid_fam	unid_genus	unid_sp	unknown
ASV_1663	unid_Phylum	unid_class	unid_order	unid_fam	unid_genus	unid_sp	unknown
ASV_1664	Ascomycota	Sordariomycetes	Xylariales	Amphisphaeriaceae	Lepteutypa	Lepteutypa_sp	phytopathogen
ASV_1665	Ascomycota	Lecanoromycetes	Lecanorales	Ramalinaceae	Bacidina	mendax	lichen
ASV_1666	unid_Phylum	unid_class	unid_order	unid_fam	unid_genus	unid_sp	unknown
ASV_1667	Basidiomycota	Pucciniomycetes	Septobasidiales	Septobasidiaceae	unid_genus	unid_sp	unknown
ASV_1668	Ascomycota	Leotiomycetes	Rhytismatales	unid_Rhytismatales_fam	Karstenia	rhopaloides	saprophyte
ASV_1669	Basidiomycota	Malasseziomycetes	Malasseziales	Malasseziaceae	Malassezia	globosa	animal commensal
ASV_1670	unid_Phylum	unid_class	unid_order	unid_fam	unid_genus	unid_sp	unknown
ASV_1671	Basidiomycota	Agaricomycetes	Agaricales	Psathyrellaceae	Coprinellus	dissemisptus	saprophyte
ASV_168	Ascomycota	Sordariomycetes	Xylariales	unid_Xylariales_fam	Asteromella	Asteromella_sp	saprophyte
ASV_1684	Basidiomycota	Tremellomycetes	Tremellales	Rhynchogastremataceae	Rhynchogastrema	visegradensis	epiphyte
ASV_1685	Ascomycota	Eurotiomycetes	Eurotiales	Aspergillaceae	Aspergillus	conicus	saprophyte
ASV_1686	Basidiomycota	Agaricomycetes	Hymenochaetales	Hymenochaetaceae	Phellinus	caribaeo-quercicola	saprophyte
ASV_1687	unid_Phylum	unid_class	unid_order	unid_fam	unid_genus	unid_sp	unknown
ASV_1688	unid_Phylum	unid_class	unid_order	unid_fam	unid_genus	unid_sp	unknown
ASV_1689	Basidiomycota	Malasseziomycetes	Malasseziales	Malasseziaceae	Malassezia	globosa	animal commensal
ASV_1690	Basidiomycota	Exobasidiomycetes	Exobasidiales	Brachybasidiaceae	Meira	Meira_sp	insecticolous
ASV_17	unid_Phylum	unid_class	unid_order	unid_fam	unid_genus	unid_sp	unknown
ASV_170	Basidiomycota	Agaricomycetes	Polyporales	Meruliaceae	Phlebia	tremellosa	saprophyte
ASV_1703	unid_Phylum	unid_class	unid_order	unid_fam	unid_genus	unid_sp	unknown
ASV_1704	unid_Phylum	unid_class	unid_order	unid_fam	unid_genus	unid_sp	unknown
ASV_1705	Ascomycota	Sordariomycetes	Xylariales	Xylariaceae	Annulohypoxylon	stygium	saprophyte
ASV_1706	Ascomycota	Dothideomycetes	Capnodiales	Mycosphaerellaceae	Xenoramularia	neerlandica	phytopathogen
ASV_1707	Ascomycota	Saccharomycetes	Saccharomycetales	Debaryomycetaceae	Meyerozyma	smithsonii	animal commensal
ASV_1708	unid_Phylum	unid_class	unid_order	unid_fam	unid_genus	unid_sp	unknown
ASV_1709	Basidiomycota	Agaricomycetes	Polyporales	Polyporaceae	Lopharia	ayresii	saprophyte
ASV_1710	Basidiomycota	Agaricomycetes	Hymenochaetales	Hymenochaetaceae	Tropicoporus	tropicalis	saprophyte
ASV_1711	Ascomycota	Dothideomycetes	Pleosporales	Pleosporaceae	Curvularia	coicis	phytopathogen
ASV_1712	unid_Phylum	unid_class	unid_order	unid_fam	unid_genus	unid_sp	unknown
ASV_1713	Ascomycota	Eurotiomycetes	Chaetothyriales	Trichomeriaceae	unid_genus	unid_sp	unknown
ASV_1714	Basidiomycota	unid_Basidiomycota_class	unid_Basidiomycota_order	unid_Basidiomycota_fam	unid_genus	unid_sp	unknown
ASV_1715	Basidiomycota	Cystobasidiomycetes	unid_Cystobasidiomycetes_order	unid_Cystobasidiomycetes_fam	unid_genus	unid_sp	unknown
ASV_1731	Ascomycota	unid_Ascomycota_class	unid_Ascomycota_order	unid_Ascomycota_fam	Tricladiella	pluvialis	saprophyte
ASV_1732	Basidiomycota	Malasseziomycetes	Malasseziales	Malasseziaceae	Malassezia	restricta	animal commensal
ASV_1733	Basidiomycota	Agaricomycetes	Russulales	Peniophoraceae	Peniophora	Peniophora_sp	saprophyte
ASV_1734	Ascomycota	Dothideomycetes	Pleosporales	Didymosphaeriaceae	unid_genus	unid_sp	phytopathogen
ASV_1735	Ascomycota	Dothideomycetes	Capnodiales	Mycosphaerellaceae	Zasmidium	queenslandicum	phytopathogen
ASV_1736	Ascomycota	Sordariomycetes	Xylariales	Sporocadaceae	Pseudopestalotiopsis	simitheae	phytopathogen
ASV_1737	Ascomycota	Dothideomycetes	Pleosporales	Thyridariaceae	Roussoella	Roussoella_sp	saprophyte
ASV_1738	Ascomycota	Dothideomycetes	Pleosporales	unid_Pleosporales_fam	unid_genus	unid_sp	unknown
ASV_1739	Basidiomycota	Agaricomycetes	Polyporales	Phanerochaetaceae	Phaeophlebiopsis	caribbeasp	saprophyte
ASV_174	Ascomycota	Laboulbeniomycetes	Pyxidiophorales	Pyxidiophoraceae	Pyxidiophora	arvernensis	fungicolous
ASV_1740	unid_Phylum	unid_class	unid_order	unid_fam	unid_genus	unid_sp	unknown
ASV_1741	Ascomycota	Sordariomycetes	Trichosphaeriales	Trichosphaeriaceae	Nigrospora	Nigrospora_sp	saprophyte
ASV_1742	Ascomycota	Eurotiomycetes	Chaetothyriales	Trichomeriaceae	Alatosessilispora	bibrachiata	unknown
ASV_1743	Basidiomycota	Exobasidiomycetes	Exobasidiales	Brachybasidiaceae	Meira	Meira_sp	insecticolous
ASV_1744	Ascomycota	Sordariomycetes	Glomerellales	Glomerellaceae	Colletotrichum	panamense	phytopathogen
ASV_1745	Basidiomycota	Malasseziomycetes	Malasseziales	Malasseziaceae	Malassezia	arunalokei	animal commensal

ASV_175	Basidiomycota	Agaricomycetes	Cantharellales	Ceratobasidiaceae	Thanatephorus	cucumeris	phytopathogen
ASV_1758	Ascomycota	unid_Ascomycota_class	unid_Ascomycota_order	unid_Ascomycota_fam	unid_genus	unid_sp	unknown
ASV_1759	Ascomycota	Eurotiomycetes	Eurotiales	Aspergillaceae	Aspergillus	Aspergillus_sp	saprophyte
ASV_1760	Basidiomycota	Agaricomycetes	Polyporales	Meruliaceae	Phlebia	tremellosa	saprophyte
ASV_1761	Basidiomycota	Agaricomycetes	Polyporales	Polyporaceae	unid_genus	unid_sp	saprophyte
ASV_1762	Basidiomycota	Agaricomycetes	Polyporales	Ganodermataceae	Ganoderma	Ganoderma_sp	saprophyte
ASV_1763	Basidiomycota	Agaricomycetes	Cantharellales	unid_Cantharellales_fam	Sistotrema	diademiferum	saprophyte
ASV_1764	unid_Phylum	unid_class	unid_order	unid_fam	unid_genus	unid_sp	unknown
ASV_1765	Basidiomycota	Agaricomycetes	Russulales	Wrightoporiaceae	Wrightoporia	tropicalis	saprophyte
ASV_1766	Ascomycota	unid_Ascomycota_class	unid_Ascomycota_order	unid_Ascomycota_fam	Tricladiella	pluvialis	saprophyte
ASV_1767	Ascomycota	Dothideomycetes	Capnodiales	Mycosphaerellaceae	unid_genus	unid_sp	phytopathogen
ASV_1768	Basidiomycota	Pucciniomycetes	Septobasidiales	Septobasidiaceae	unid_genus	unid_sp	unknown
ASV_1779	unid_Phylum	unid_class	unid_order	unid_fam	unid_genus	unid_sp	unknown
ASV_1780	Basidiomycota	Agaricomycetes	Agaricales	Strophariaceae	Psilocybe	Psilocybe_sp	saprophyte
ASV_1781	Ascomycota	Dothideomycetes	Pleosporales	Pleosporaceae	Curvularia	coicis	phytopathogen
ASV_1782	Basidiomycota	Agaricomycetes	Polyporales	Meruliaceae	Phlebiporia	Phlebiporia_sp	saprophyte
ASV_1783	Basidiomycota	Agaricomycetes	Agaricales	Pterulaceae	unid_genus	unid_sp	saprophyte
ASV_1784	Basidiomycota	Agaricomycetes	Polyporales	Irpicaceae	Ceriporia	alachuana	saprophyte
ASV_1785	Ascomycota	Dothideomycetes	Pleosporales	unid_Pleosporales_fam	unid_genus	unid_sp	unknown
ASV_1786	unid_Phylum	unid_class	unid_order	unid_fam	unid_genus	unid_sp	unknown
ASV_1787	Basidiomycota	Agaricomycetes	Agaricales	Agaricaceae	Agaricus	Agaricus_sp	saprophyte
ASV_1788	unid_Phylum	unid_class	unid_order	unid_fam	unid_genus	unid_sp	unknown
ASV_1789	Ascomycota	Dothideomycetes	Venturiales	Sympoventuriaceae	Ochroconis	Ochroconis_sp	phytopathogen
ASV_179	Basidiomycota	Agaricomycetes	unid_Agaricomycetes_order	unid_Agaricomycetes_fam	unid_genus	unid_sp	unknown
ASV_1790	Ascomycota	Dothideomycetes	Pleosporales	Pleosporaceae	Curvularia	Curvularia_sp	phytopathogen
ASV_1791	Ascomycota	Dothideomycetes	Pleosporales	unid_Pleosporales_fam	unid_genus	unid_sp	unknown
ASV_18	Ascomycota	Laboulbeniomycetes	Pyxidiophorales	Pyxidiophoraceae	Pyxidiophora	arvernensis	fungicolous
ASV_1814	Ascomycota	Dothideomycetes	Pleosporales	Didymosphaeriaceae	Paraconiothyrium	Paraconiothyrium_sp	fungicolous
ASV_1815	Basidiomycota	Microbotryomycetes	Sporidiobolales	Sporidiobolaceae	unid_genus	unid_sp	saprophyte
ASV_1816	unid_Phylum	unid_class	unid_order	unid_fam	unid_genus	unid_sp	unknown
ASV_1817	unid_Phylum	unid_class	unid_order	unid_fam	unid_genus	unid_sp	unknown
ASV_1818	Ascomycota	Eurotiomycetes	Chaetothyriales	Trichomeriaceae	unid_genus	unid_sp	unknown
ASV_1819	unid_Phylum	unid_class	unid_order	unid_fam	unid_genus	unid_sp	unknown
ASV_182	Basidiomycota	Exobasidiomycetes	Georgefischeriales	Tilletiariaceae	Tilletiaria	anomala	unknown
ASV_1820	Basidiomycota	Malasseziomycetes	Malasseziales	Malasseziaceae	unid_genus	unid_sp	animal commensal
ASV_1821	Basidiomycota	Malasseziomycetes	Malasseziales	Malasseziaceae	Malassezia	globosa	animal commensal
ASV_1822	Basidiomycota	Agaricomycetes	Agaricales	Psathyrellaceae	Coprinellus	dissemisptus	saprophyte
ASV_1823	Ascomycota	unid_Ascomycota_class	unid_Ascomycota_order	unid_Ascomycota_fam	unid_genus	unid_sp	unknown
ASV_183	Basidiomycota	Agaricomycetes	Sebacinales	unid_Sebacinales_fam	unid_genus	unid_sp	unknown
ASV_1839	Basidiomycota	unid_Basidiomycota_class	unid_Basidiomycota_order	unid_Basidiomycota_fam	unid_genus	unid_sp	unknown
ASV_1840	Basidiomycota	Agaricomycetes	Corticiales	Vuilleminiaceae	Vuilleminia	Vuilleminia_sp	saprophyte
ASV_1841	unid_Phylum	unid_class	unid_order	unid_fam	unid_genus	unid_sp	unknown
ASV_1842	Ascomycota	Sordariomycetes	Diaporthales	Schizoparmaceae	Coniella	Coniella_sp	phytopathogen
ASV_1843	Ascomycota	Sordariomycetes	Hypoceales	Catabotrydaceae	Botryosporium	Botryosporium_sp	phytopathogen
ASV_1844	unid_Phylum	unid_class	unid_order	unid_fam	unid_genus	unid_sp	unknown
ASV_1845	Basidiomycota	Agaricomycetes	Polyporales	Ganodermataceae	Ganoderma	Ganoderma_sp	saprophyte
ASV_1846	Basidiomycota	Agaricomycetes	Cantharellales	unid_Cantharellales_fam	unid_genus	unid_sp	saprophyte
ASV_1847	unid_Phylum	unid_class	unid_order	unid_fam	unid_genus	unid_sp	unknown
ASV_1848	unid_Phylum	unid_class	unid_order	unid_fam	unid_genus	unid_sp	unknown
ASV_1849	unid_Phylum	unid_class	unid_order	unid_fam	unid_genus	unid_sp	unknown

ASV_1850	Ascomycota	unid_Ascomycota_class	unid_Ascomycota_order	unid_Ascomycota_fam	unid_genus	unid_sp	unknown
ASV_186	unid_Phylum	unid_class	unid_order	unid_fam	unid_genus	unid_sp	unknown
ASV_1862	Ascomycota	Saccharomycetes	Saccharomycetales	Phaffomycetaceae	Wickerhamomyces	Wickerhamomyces_sp	insect symbiont
ASV_1863	Ascomycota	Dothideomycetes	Capnodiales	Mycosphaerellaceae	unid_genus	unid_sp	phytopathogen
ASV_1864	unid_Phylum	unid_class	unid_order	unid_fam	unid_genus	unid_sp	unknown
ASV_1865	Basidiomycota	Agaricomycetes	Polyporales	Fomitopsidaceae	Melanoporia	nigra	saprophyte
ASV_1866	Ascomycota	Dothideomycetes	Pleosporales	unid_Pleosporales_fam	unid_genus	unid_sp	unknown
ASV_1867	Ascomycota	Sordariomycetes	Xylariales	Xylariaceae	Camillea	tinctor	saprophyte
ASV_1868	Ascomycota	Sordariomycetes	Chaetosphaeriales	Chaetosphaeriaceae	Thozetella	Thozetella_sp	saprophyte
ASV_1869	Ascomycota	Dothideomycetes	Pleosporales	Arthopyreniaceae	unid_genus	unid_sp	unknown
ASV_1884	unid_Phylum	unid_class	unid_order	unid_fam	unid_genus	unid_sp	unknown
ASV_1885	Basidiomycota	Agaricomycetes	Agaricales	Psathyrellaceae	Coprinopsis	friesii	saprophyte
ASV_1886	Basidiomycota	Agaricomycetes	Russulales	Lachnocladiaceae	Vararia	rugosispora	saprophyte
ASV_1887	Basidiomycota	Agaricomycetes	Agaricales	Agaricaceae	unid_genus	unid_sp	saprophyte
ASV_1888	Ascomycota	Dothideomycetes	Pleosporales	Pleosporaceae	Curvularia	americana	phytopathogen
ASV_1889	Ascomycota	Sordariomycetes	Xylariales	Xylariaceae	Nemania	primolutea	saprophyte
ASV_1890	Ascomycota	Orbiliomycetes	unid_Orbiliomycetes_order	unid_Orbiliomycetes_fam	unid_genus	unid_sp	unknown
ASV_1891	Basidiomycota	Agaricomycetes	Agaricales	unid_Agaricales_fam	unid_genus	unid_sp	saprophyte
ASV_1892	Ascomycota	Dothideomycetes	Pleosporales	Pleosporaceae	Alternaria	betae-kenyensis	saprophyte
ASV_1893	Ascomycota	Sordariomycetes	Diaporthales	Diaporthaceae	Diaporthe	Diaporthe_sp	phytopathogen
ASV_1894	Ascomycota	Sordariomycetes	Hypocreales	Nectriaceae	Fusarium	Fusarium_sp	unknown
ASV_1895	Basidiomycota	Agaricomycetes	Corticiales	Vuilleminiaceae	Vuilleminia	Vuilleminia_sp	saprophyte
ASV_19	unid_Phylum	unid_class	unid_order	unid_fam	unid_genus	unid_sp	unknown
ASV_190	unid_Phylum	unid_class	unid_order	unid_fam	unid_genus	unid_sp	unknown
ASV_1913	Ascomycota	Dothideomycetes	Botryosphaeriales	Botryosphaeriaceae	Lasiodiplodia	Lasiodiplodia_sp	phytopathogen
ASV_1914	Ascomycota	Saccharomycetes	Saccharomycetales	Saccharomycetaceae	Saccharomyces	paradoxus	animal commensal
ASV_1915	Ascomycota	Eurotiomycetes	Chaetothyriales	unid_Chaetothyriales_fam	Neostrelitziana	acaciigena	unknown
ASV_1916	Basidiomycota	Agaricomycetes	Agaricales	Strophariaceae	Galerina	sulciceps	saprophyte
ASV_1917	Ascomycota	unid_Ascomycota_class	unid_Ascomycota_order	unid_Ascomycota_fam	Tricladiella	pluvialis	saprophyte
ASV_1918	Basidiomycota	Exobasidiomycetes	Entylomatales	unid_Entylomatales_fam	Tilletiopsis	washingtonensis	saprophyte
ASV_1919	Basidiomycota	Pucciniomycetes	Septobasidiales	Septobasidiaceae	Septobasidium	Septobasidium_sp	insecticolous
ASV_192	unid_Phylum	unid_class	unid_order	unid_fam	unid_genus	unid_sp	unknown
ASV_1920	Ascomycota	Dothideomycetes	Capnodiales	Cladosporiaceae	Cladosporium	Cladosporium_sp	saprophyte
ASV_1921	unid_Phylum	unid_class	unid_order	unid_fam	unid_genus	unid_sp	unknown
ASV_1922	Ascomycota	Dothideomycetes	Pleosporales	Cucurbitariaceae	Pyrenochaetopsis	leptospora	phytopathogen
ASV_1923	Basidiomycota	Agaricomycetes	Cantharellales	Botryobasidiaceae	unid_genus	unid_sp	saprophyte
ASV_1924	Ascomycota	Sordariomycetes	Xylariales	unid_Xylariales_fam	unid_genus	unid_sp	unknown
ASV_1925	Basidiomycota	Agaricomycetes	Cantharellales	Ceratobasidiaceae	Thanatephorus	cucumeris	phytopathogen
ASV_1926	Ascomycota	Eurotiomycetes	Chaetothyriales	unid_Chaetothyriales_fam	Hyalocladosporiella	tectonae	fungicolous
ASV_1927	Ascomycota	Sordariomycetes	Glomerellales	Glomerellaceae	Colletotrichum	cairnsense	phytopathogen
ASV_1928	Ascomycota	Dothideomycetes	Pleosporales	Pleosporaceae	Curvularia	coicis	phytopathogen
ASV_1929	Ascomycota	Eurotiomycetes	Chaetothyriales	Cyphellophoraceae	Cyphellophora	eucalypti	saprophyte
ASV_193	unid_Phylum	unid_class	unid_order	unid_fam	unid_genus	unid_sp	unknown
ASV_194	unid_Phylum	unid_class	unid_order	unid_fam	unid_genus	unid_sp	unknown
ASV_1944	Ascomycota	Dothideomycetes	Pleosporales	unid_Pleosporales_fam	unid_genus	unid_sp	unknown
ASV_1945	Basidiomycota	unid_Basidiomycota_class	unid_Basidiomycota_order	unid_Basidiomycota_fam	unid_genus	unid_sp	unknown
ASV_1946	unid_Phylum	unid_class	unid_order	unid_fam	unid_genus	unid_sp	unknown
ASV_1947	Ascomycota	Dothideomycetes	Pleosporales	Neomassarinaceae	Neomassarina	pandanicola	saprophyte
ASV_1948	Ascomycota	unid_Ascomycota_class	unid_Ascomycota_order	unid_Ascomycota_fam	Tricladiella	pluvialis	saprophyte
ASV_1949	Ascomycota	Sordariomycetes	Xylariales	Xylariaceae	Annulohypoxylon	stygium	saprophyte

ASV_195	Basidiomycota	Exobasidiomycetes	Exobasidiales	Exobasidiaceae	Exobasidium	Exobasidium_sp	phytopathogen
ASV_1950	Ascomycota	Dothideomycetes	Venturiales	Sympoventuriaceae	Ochroconis	Ochroconis_sp	phytopathogen
ASV_1951	Ascomycota	Sordariomycetes	Hypocreales	Cordycipitaceae	Cordyceps	Cordyceps_sp	insecticolous
ASV_1952	Ascomycota	Eurotiomycetes	Chaetothyriales	Cyphellophoraceae	Cyphellophora	Cyphellophora_sp	saprophyte
ASV_1953	unid_Phylum	unid_class	unid_order	unid_fam	unid_genus	unid_sp	unknown
ASV_1954	Basidiomycota	Agaricomycetes	Agaricales	Tricholomataceae	unid_genus	unid_sp	saprophyte
ASV_1955	Ascomycota	Sordariomycetes	Hypocreales	Bionectriaceae	Clonostachys	rosea	saprophyte
ASV_1956	unid_Phylum	unid_class	unid_order	unid_fam	unid_genus	unid_sp	unknown
ASV_196	unid_Phylum	unid_class	unid_order	unid_fam	unid_genus	unid_sp	unknown
ASV_197	Basidiomycota	Malasseziomycetes	Malasseziales	Malasseziaceae	Malassezia	arunalokei	animal commensal
ASV_1979	unid_Phylum	unid_class	unid_order	unid_fam	unid_genus	unid_sp	unknown
ASV_1980	Basidiomycota	Agaricomycetes	Auriculariales	unid_Auriculariales_fam	unid_genus	unid_sp	saprophyte
ASV_1981	unid_Phylum	unid_class	unid_order	unid_fam	unid_genus	unid_sp	unknown
ASV_1982	unid_Phylum	unid_class	unid_order	unid_fam	unid_genus	unid_sp	unknown
ASV_1983	unid_Phylum	unid_class	unid_order	unid_fam	unid_genus	unid_sp	unknown
ASV_1984	Ascomycota	Sordariomycetes	Xylariales	Xylariaceae	Annulohypoxylon	Annulohypoxylon_sp	saprophyte
ASV_1985	unid_Phylum	unid_class	unid_order	unid_fam	unid_genus	unid_sp	unknown
ASV_1986	Basidiomycota	Pucciniomycetes	Septobasidiales	Septobasidiaceae	Septobasidium	Septobasidium_sp	insecticolous
ASV_1987	Basidiomycota	Agaricomycetes	Polyporales	Polyporaceae	unid_genus	unid_sp	saprophyte
ASV_1988	Ascomycota	Dothideomycetes	Pleosporales	unid_Pleosporales_fam	unid_genus	unid_sp	unknown
ASV_1989	Basidiomycota	Agaricomycetes	Auriculariales	Hyaloriaceae	Myxarium	Myxarium_sp	saprophyte
ASV_1990	Ascomycota	Dothideomycetes	Capnodiales	unid_Capnodiales_fam	unid_genus	unid_sp	saprophyte
ASV_1991	Ascomycota	unid_Ascomycota_class	unid_Ascomycota_order	unid_Ascomycota_fam	unid_genus	unid_sp	unknown
ASV_1992	Basidiomycota	Agaricomycetes	Agaricales	Marasmiaceae	Campanella	Campanella_sp	saprophyte
ASV_1993	Ascomycota	Sordariomycetes	Diaporthales	Diaporthaceae	Diaporthe	tectospe	phytopathogen
ASV_1994	Ascomycota	Eurotiomycetes	Chaetothyriales	Trichomeriaceae	unid_genus	unid_sp	unknown
ASV_2	Ascomycota	Sordariomycetes	Hypocreales	Nectriaceae	Fusarium	waltergamsii	saprophyte
ASV_2013	Ascomycota	Sordariomycetes	Glomerellales	Glomerellaceae	Colletotrichum	ignotum	phytopathogen
ASV_2014	unid_Phylum	unid_class	unid_order	unid_fam	unid_genus	unid_sp	unknown
ASV_2015	Basidiomycota	Tremellomycetes	Cystofilobasidiales	Cystofilobasidiaceae	Cystofilobasidium	capitatum	animal commensal
ASV_2016	Basidiomycota	Tremellomycetes	Tremellales	Bulleribasidiaceae	Derxomyces	pseudoschimicola	unknown
ASV_2017	Ascomycota	Eurotiomycetes	Phaeomoniellales	Phaeomoniellaceae	Phaeomoniella	Phaeomoniella_sp	phytopathogen
ASV_2018	Ascomycota	Lecanoromycetes	Lecanorales	Ramalinaceae	Bacidia	neosquamulosa	lichen
ASV_2019	unid_Phylum	unid_class	unid_order	unid_fam	unid_genus	unid_sp	unknown
ASV_2020	unid_Phylum	unid_class	unid_order	unid_fam	unid_genus	unid_sp	unknown
ASV_2021	Ascomycota	Sordariomycetes	Hypocreales	unid_Hypocreales_fam	unid_genus	unid_sp	unknown
ASV_2022	unid_Phylum	unid_class	unid_order	unid_fam	unid_genus	unid_sp	unknown
ASV_2023	Ascomycota	Sordariomycetes	Chaetosphaeriales	Chaetosphaeriaceae	Dinemasporium	spinificis	saprophyte
ASV_2024	Basidiomycota	Agaricomycetes	Polyporales	unid_Polyporales_fam	unid_genus	unid_sp	saprophyte
ASV_2025	Basidiomycota	Pucciniomycetes	Septobasidiales	Septobasidiaceae	Septobasidium	Septobasidium_sp	insecticolous
ASV_2026	unid_Phylum	unid_class	unid_order	unid_fam	unid_genus	unid_sp	unknown
ASV_2027	Basidiomycota	Agaricomycetes	Corticiales	Corticiaceae	unid_genus	unid_sp	unknown
ASV_2028	Basidiomycota	Agaricomycetes	Polyporales	Meruliaceae	Scopuloides	Scopuloides_sp	saprophyte
ASV_2029	unid_Phylum	unid_class	unid_order	unid_fam	unid_genus	unid_sp	unknown
ASV_203	unid_Phylum	unid_class	unid_order	unid_fam	unid_genus	unid_sp	unknown
ASV_2030	Ascomycota	Saccharomycetes	Saccharomycetales	Phaffomycetaceae	Cyberlindnera	jadinii	animal commensal
ASV_2031	Basidiomycota	Agaricostilbomycetes	Agaricostilbales	Chionosphaeraceae	unid_genus	unid_sp	unknown
ASV_2032	Basidiomycota	Agaricomycetes	Agaricales	Omphalotaceae	Neonothopanus	spmbi	saprophyte
ASV_2033	Ascomycota	Sordariomycetes	Diaporthales	Diaporthaceae	Diaporthe	Diaporthe_sp	phytopathogen
ASV_2034	Basidiomycota	Agaricomycetes	Boletales	Coniophoraceae	Coniophora	hanoiensis	saprophyte

ASV_2035	Ascomycota	Sordariomycetes	Xylariales	Beltraniaceae	Beltrania	pseudorhombica	phytopathogen
ASV_2036	Ascomycota	Dothideomycetes	Venturiales	Sympoventuriaceae	Ochroconis	Ochroconis_sp	phytopathogen
ASV_2037	Basidiomycota	Agaricomycetes	Trechisporales	unid_Trechisporales_fam	unid_genus	unid_sp	saprophyte
ASV_2038	Basidiomycota	Cystobasidiomycetes	unid_Cystobasidiomycetes_order	unid_Cystobasidiomycetes_fam	unid_genus	unid_sp	unknown
ASV_204	unid_Phylum	unid_class	unid_order	unid_fam	unid_genus	unid_sp	unknown
ASV_206	unid_Phylum	unid_class	unid_order	unid_fam	unid_genus	unid_sp	unknown
ASV_2067	Basidiomycota	Exobasidiomycetes	Exobasidiales	Brachybasidiaceae	Meira	Meira_sp	insecticolous
ASV_2068	Basidiomycota	Exobasidiomycetes	Exobasidiales	Exobasidiaceae	Exobasidium	Exobasidium_sp	phytopathogen
ASV_2069	unid_Phylum	unid_class	unid_order	unid_fam	unid_genus	unid_sp	unknown
ASV_2070	Ascomycota	Sordariomycetes	Sordariales	Helminthosphaeriaceae	Spadicoides	Spadicoides_sp	saprophyte
ASV_2071	Ascomycota	unid_Ascomycota_class	unid_Ascomycota_order	unid_Ascomycota_fam	Tricladiella	pluvialis	saprophyte
ASV_2072	Ascomycota	Sordariomycetes	Xylariales	unid_Xylariales_fam	unid_genus	unid_sp	unknown
ASV_2073	unid_Phylum	unid_class	unid_order	unid_fam	unid_genus	unid_sp	unknown
ASV_2074	Ascomycota	Eurotiomycetes	Chaetothyriales	Cyphellophoraceae	Cyphellophora	jingdongensis	saprophyte
ASV_2075	Basidiomycota	Agaricostilbomycetes	Agaricostilbales	Chionosphaeraceae	unid_genus	unid_sp	unknown
ASV_2076	Ascomycota	Dothideomycetes	Pleosporales	Didymosphaeriaceae	Spegazzinia	tessarthra	phytopathogen
ASV_2077	Basidiomycota	Pucciniomycetes	Septobasidiales	Septobasidiaceae	Septobasidium	sinuosum	insecticolous
ASV_2078	unid_Phylum	unid_class	unid_order	unid_fam	unid_genus	unid_sp	unknown
ASV_2079	Ascomycota	Sordariomycetes	Hypocreales	Bionectriaceae	Clonostachys	Clonostachys_sp	saprophyte
ASV_208	unid_Phylum	unid_class	unid_order	unid_fam	unid_genus	unid_sp	unknown
ASV_2080	Ascomycota	Sordariomycetes	Hypocreales	Nectriaceae	Fusarium	asiaticum	phytopathogen
ASV_2081	Ascomycota	Eurotiomycetes	Eurotiales	Aspergillaceae	Penicillium	alfredii	saprophyte
ASV_2082	unid_Phylum	unid_class	unid_order	unid_fam	unid_genus	unid_sp	unknown
ASV_2083	Ascomycota	Sordariomycetes	Trichosphaeriales	Trichosphaeriaceae	Nigrospora	Nigrospora_sp	saprophyte
ASV_2084	Ascomycota	Eurotiomycetes	Chaetothyriales	Trichomeriaceae	unid_genus	unid_sp	unknown
ASV_2085	Basidiomycota	Tremellomycetes	Tremellales	Sirobasidiaceae	Sirobasidium	brefeldianum	unknown
ASV_2086	unid_Phylum	unid_class	unid_order	unid_fam	unid_genus	unid_sp	unknown
ASV_2087	Ascomycota	unid_Ascomycota_class	unid_Ascomycota_order	unid_Ascomycota_fam	unid_genus	unid_sp	unknown
ASV_2088	Ascomycota	Sordariomycetes	Xylariales	unid_Xylariales_fam	unid_genus	unid_sp	unknown
ASV_2089	Ascomycota	Sordariomycetes	Glomerellales	Glomerellaceae	Colletotrichum	hymenocallidicola	phytopathogen
ASV_2090	unid_Phylum	unid_class	unid_order	unid_fam	unid_genus	unid_sp	unknown
ASV_2091	Basidiomycota	Agaricomycetes	unid_Agaricomycetes_order	unid_Agaricomycetes_fam	unid_genus	unid_sp	unknown
ASV_2092	Ascomycota	Dothideomycetes	Venturiales	Sympoventuriaceae	Ochroconis	aquatica	phytopathogen
ASV_2093	Ascomycota	Sordariomycetes	Glomerellales	Glomerellaceae	Colletotrichum	Colletotrichum_sp	phytopathogen
ASV_210	Ascomycota	Dothideomycetes	Pleosporales	Morosphaeriaceae	Acrocalymma	Acrocalymma_sp	phytopathogen
ASV_2114	unid_Phylum	unid_class	unid_order	unid_fam	unid_genus	unid_sp	unknown
ASV_2115	Ascomycota	Sordariomycetes	Hypocreales	Nectriaceae	Fusicolla	merismoides	fungicolous
ASV_2116	unid_Phylum	unid_class	unid_order	unid_fam	unid_genus	unid_sp	unknown
ASV_2117	Ascomycota	Dothideomycetes	Pleosporales	Lophiostomataceae	Vaginatispora	amygdali	saprophyte
ASV_2118	Basidiomycota	Agaricomycetes	Polyporales	Phanerochaetaceae	Phaeophlebiopsis	caribbeasp	saprophyte
ASV_2119	unid_Phylum	unid_class	unid_order	unid_fam	unid_genus	unid_sp	unknown
ASV_212	Ascomycota	Sordariomycetes	Glomerellales	Glomerellaceae	Colletotrichum	Colletotrichum_sp	phytopathogen
ASV_2120	unid_Phylum	unid_class	unid_order	unid_fam	unid_genus	unid_sp	unknown
ASV_2121	Basidiomycota	Agaricomycetes	Polyporales	Polyporaceae	Trametes	Trametes_sp	saprophyte
ASV_2122	Basidiomycota	Agaricostilbomycetes	Agaricostilbales	Chionosphaeraceae	unid_genus	unid_sp	unknown
ASV_2123	Ascomycota	Dothideomycetes	Pleosporales	Didymellaceae	Stagonosporopsis	Stagonosporopsis_sp	phytopathogen
ASV_2124	Ascomycota	Sordariomycetes	Xylariales	Sporocadaceae	Discosia	Discosia_sp	phytopathogen
ASV_2125	unid_Phylum	unid_class	unid_order	unid_fam	unid_genus	unid_sp	unknown
ASV_2126	Basidiomycota	Agaricomycetes	Cantharellales	Ceratobasidiaceae	Thanatephorus	cucumeris	phytopathogen
ASV_2127	Ascomycota	Dothideomycetes	unid_Dothideomycetes_order	unid_Dothideomycetes_fam	unid_genus	unid_sp	unknown

ASV_2128	Basidiomycota	Agaricomycetes	Polyporales	Ganodermataceae	Ganoderma	Ganoderma_sp	saprophyte
ASV_2129	unid_Phylum	unid_class	unid_order	unid_fam	unid_genus	unid_sp	unknown
ASV_213	Ascomycota	Dothideomycetes	Pleosporales	Pleosporaceae	Edenia	gomezpompae	unknown
ASV_2130	unid_Phylum	unid_class	unid_order	unid_fam	unid_genus	unid_sp	unknown
ASV_2131	Ascomycota	unid_Ascomycota_class	unid_Ascomycota_order	unid_Ascomycota_fam	unid_genus	unid_sp	unknown
ASV_2132	Basidiomycota	Exobasidiomycetes	Exobasidiales	Brachybasidiaceae	Meira	Meira_sp	insecticolous
ASV_2133	Ascomycota	Sordariomycetes	Glomerellales	Plectosphaerellaceae	Plectosphaerella	Plectosphaerella_sp	phytopathogen
ASV_2134	unid_Phylum	unid_class	unid_order	unid_fam	unid_genus	unid_sp	unknown
ASV_2135	unid_Phylum	unid_class	unid_order	unid_fam	unid_genus	unid_sp	unknown
ASV_2136	Ascomycota	unid_Ascomycota_class	unid_Ascomycota_order	unid_Ascomycota_fam	unid_genus	unid_sp	unknown
ASV_214	Basidiomycota	Exobasidiomycetes	Georgefischeriales	unid_Georgefischeriales_fam	unid_genus	unid_sp	unknown
ASV_216	Ascomycota	Sordariomycetes	Hypocreales	Nectriaceae	Fusarium	concentricum	phytopathogen
ASV_218	Basidiomycota	Agaricomycetes	Russulales	Stereaceae	Stereum	complicatum	saprophyte
ASV_221	unid_Phylum	unid_class	unid_order	unid_fam	unid_genus	unid_sp	unknown
ASV_222	Ascomycota	Sordariomycetes	Hypocreales	Nectriaceae	Fusarium	waltergamsii	saprophyte
ASV_226	Basidiomycota	Pucciniomycetes	Septobasidiales	Septobasidiaceae	unid_genus	unid_sp	unknown
ASV_228	unid_Phylum	unid_class	unid_order	unid_fam	unid_genus	unid_sp	unknown
ASV_23	unid_Phylum	unid_class	unid_order	unid_fam	unid_genus	unid_sp	unknown
ASV_230	Ascomycota	Saccharomycetes	Saccharomycetales	Phaffomycetaceae	Wickerhamomyces	Wickerhamomyces_sp	insect symbiont
ASV_231	Basidiomycota	Agaricomycetes	Polyporales	Polyporaceae	Trametes	versicolor	saprophyte
ASV_232	Basidiomycota	Microbotryomycetes	unid_Microbotryomycetes_order	unid_Microbotryomycetes_fam	unid_genus	unid_sp	unknown
ASV_233	Ascomycota	Laboulbeniomycetes	Pyxidiophorales	Pyxidiophoraceae	Pyxidiophora	arvernensis	fungicolous
ASV_235	Basidiomycota	Ustilaginomycetes	Ustilaginales	Ustilaginaceae	Moesziomyces	antarcticus	phytopathogen
ASV_236	unid_Phylum	unid_class	unid_order	unid_fam	unid_genus	unid_sp	unknown
ASV_239	unid_Phylum	unid_class	unid_order	unid_fam	unid_genus	unid_sp	unknown
ASV_240	Basidiomycota	Agaricomycetes	unid_Agaricomycetes_order	unid_Agaricomycetes_fam	unid_genus	unid_sp	unknown
ASV_241	unid_Phylum	unid_class	unid_order	unid_fam	unid_genus	unid_sp	unknown
ASV_243	Ascomycota	Sordariomycetes	Xylariales	Sporocadaceae	Neopestalotiopsis	saprophytica	phytopathogen
ASV_244	unid_Phylum	unid_class	unid_order	unid_fam	unid_genus	unid_sp	unknown
ASV_246	Basidiomycota	Malasseziomycetes	Malasseziales	Malasseziaceae	Malassezia	restricta	animal commensal
ASV_247	unid_Phylum	unid_class	unid_order	unid_fam	unid_genus	unid_sp	unknown
ASV_248	Basidiomycota	Malasseziomycetes	Malasseziales	Malasseziaceae	Malassezia	arunalokei	animal commensal
ASV_249	Basidiomycota	Agaricomycetes	Polyporales	Polyporaceae	Lopharia	ayresii	saprophyte
ASV_25	Basidiomycota	unid_Basidiomycota_class	unid_Basidiomycota_order	unid_Basidiomycota_fam	unid_genus	unid_sp	unknown
ASV_250	Ascomycota	Dothideomycetes	Pleosporales	Cucurbitariaceae	Pyrenochaeta	inflorescentiae	phytopathogen
ASV_252	unid_Phylum	unid_class	unid_order	unid_fam	unid_genus	unid_sp	unknown
ASV_254	Basidiomycota	Agaricostilbomycetes	Agaricostilbales	Chionosphaeraceae	unid_genus	unid_sp	unknown
ASV_255	Basidiomycota	Agaricomycetes	unid_Agaricomycetes_order	unid_Agaricomycetes_fam	unid_genus	unid_sp	unknown
ASV_261	unid_Phylum	unid_class	unid_order	unid_fam	unid_genus	unid_sp	unknown
ASV_262	Ascomycota	Dothideomycetes	Pleosporales	Morosphaeriaceae	Acrocalymma	Acrocalymma_sp	phytopathogen
ASV_263	unid_Phylum	unid_class	unid_order	unid_fam	unid_genus	unid_sp	unknown
ASV_264	unid_Phylum	unid_class	unid_order	unid_fam	unid_genus	unid_sp	unknown
ASV_268	unid_Phylum	unid_class	unid_order	unid_fam	unid_genus	unid_sp	unknown
ASV_269	unid_Phylum	unid_class	unid_order	unid_fam	unid_genus	unid_sp	unknown
ASV_270	Basidiomycota	Agaricomycetes	Polyporales	Polyporaceae	Trametes	versicolor	saprophyte
ASV_271	unid_Phylum	unid_class	unid_order	unid_fam	unid_genus	unid_sp	unknown
ASV_274	Basidiomycota	Wallemiomycetes	Wallemiales	Wallemiaceae	Wallemia	canadensis	animal commensal
ASV_275	Basidiomycota	Agaricomycetes	Cantharellales	unid_Cantharellales_fam	Sistotrema	brinkmannii	saprophyte
ASV_276	unid_Phylum	unid_class	unid_order	unid_fam	unid_genus	unid_sp	unknown
ASV_278	unid_Phylum	unid_class	unid_order	unid_fam	unid_genus	unid_sp	unknown

ASV_28	Ascomycota	Laboulbeniomycetes	Pyxidiophorales	Pyxidiophoraceae	Pyxidiophora	arvernensis	fungicolous
ASV_280	Basidiomycota	Exobasidiomycetes	Georgefischeriales	Tilletiariaceae	Tilletiaria	anomala	unknown
ASV_283	Basidiomycota	Agaricomycetes	unid_Agaricomycetes_order	unid_Agaricomycetes_fam	unid_genus	unid_sp	unknown
ASV_284	unid_Phylum	unid_class	unid_order	unid_fam	unid_genus	unid_sp	unknown
ASV_286	Basidiomycota	Agaricomycetes	Trechisporales	Hydnodontaceae	Brevicellicium	olivascens	saprophyte
ASV_287	Ascomycota	Dothideomycetes	Pleosporales	Cucurbitariaceae	Pyrenochaeta	inflorescentiae	phytopathogen
ASV_288	unid_Phylum	unid_class	unid_order	unid_fam	unid_genus	unid_sp	unknown
ASV_292	unid_Phylum	unid_class	unid_order	unid_fam	unid_genus	unid_sp	unknown
ASV_296	unid_Phylum	unid_class	unid_order	unid_fam	unid_genus	unid_sp	unknown
ASV_297	Basidiomycota	Wallemiomycetes	Wallemiales	Wallemiaceae	Wallemia	canadensis	animal commensal
ASV_3	unid_Phylum	unid_class	unid_order	unid_fam	unid_genus	unid_sp	unknown
ASV_304	unid_Phylum	unid_class	unid_order	unid_fam	unid_genus	unid_sp	unknown
ASV_306	Basidiomycota	Agaricomycetes	Cantharellales	unid_Cantharellales_fam	Burgoa	verzuolia	saprophyte
ASV_309	unid_Phylum	unid_class	unid_order	unid_fam	unid_genus	unid_sp	unknown
ASV_312	Ascomycota	Dothideomycetes	Pleosporales	Pleosporaceae	Edenia	gomezpompae	unknown
ASV_314	Ascomycota	Dothideomycetes	Pleosporales	unid_Pleosporales_fam	unid_genus	unid_sp	unknown
ASV_315	Ascomycota	Saccharomycetes	Saccharomycetales	Phaffomycetaceae	Wickerhamomyces	Wickerhamomyces_sp	insect symbiont
ASV_317	unid_Phylum	unid_class	unid_order	unid_fam	unid_genus	unid_sp	unknown
ASV_325	Basidiomycota	Wallemiomycetes	Wallemiales	Wallemiaceae	Wallemia	muriae	animal commensal
ASV_326	Basidiomycota	Agaricomycetes	Russulales	Stereaceae	Stereum	complicatum	saprophyte
ASV_327	unid_Phylum	unid_class	unid_order	unid_fam	unid_genus	unid_sp	unknown
ASV_328	unid_Phylum	unid_class	unid_order	unid_fam	unid_genus	unid_sp	unknown
ASV_329	unid_Phylum	unid_class	unid_order	unid_fam	unid_genus	unid_sp	unknown
ASV_330	Ascomycota	Sordariomycetes	Xylariales	Sporocadaceae	Pseudopestalotiopsis	simitheae	phytopathogen
ASV_331	Basidiomycota	Malasseziomycetes	Malasseziales	Malasseziaceae	Malassezia	restricta	animal commensal
ASV_332	unid_Phylum	unid_class	unid_order	unid_fam	unid_genus	unid_sp	unknown
ASV_333	Ascomycota	Eurotiomycetes	Eurotiales	Aspergillaceae	Penicillium	alfredii	saprophyte
ASV_335	Ascomycota	Eurotiomycetes	Chaetothyriales	Trichomeriaceae	unid_genus	unid_sp	unknown
ASV_336	Basidiomycota	Pucciniomycetes	Septobasidiales	Septobasidiaceae	Septobasidium	Septobasidium_sp	insecticolous
ASV_337	Basidiomycota	Wallemiomycetes	Wallemiales	Wallemiaceae	Wallemia	canadensis	animal commensal
ASV_338	Basidiomycota	Wallemiomycetes	Wallemiales	Wallemiaceae	Wallemia	canadensis	animal commensal
ASV_34	Basidiomycota	Wallemiomycetes	Wallemiales	Wallemiaceae	Wallemia	muriae	animal commensal
ASV_340	Ascomycota	Dothideomycetes	Botryosphaeriales	Phyllostictaceae	Phyllosticta	catimbauensis	phytopathogen
ASV_341	Ascomycota	Dothideomycetes	Pleosporales	Pleosporaceae	Alternaria	betae-kenyensis	saprophyte
ASV_343	unid_Phylum	unid_class	unid_order	unid_fam	unid_genus	unid_sp	unknown
ASV_344	Basidiomycota	Agaricomycetes	Agaricales	Psathyrellaceae	Coprinellus	dissemisptus	saprophyte
ASV_347	Basidiomycota	Malasseziomycetes	Malasseziales	Malasseziaceae	Malassezia	arunalokei	animal commensal
ASV_35	unid_Phylum	unid_class	unid_order	unid_fam	unid_genus	unid_sp	unknown
ASV_351	Basidiomycota	Wallemiomycetes	Wallemiales	Wallemiaceae	Wallemia	muriae	animal commensal
ASV_353	unid_Phylum	unid_class	unid_order	unid_fam	unid_genus	unid_sp	unknown
ASV_357	Basidiomycota	Agaricomycetes	Hymenochaetales	Hymenochaetaceae	Hymenochaetopsis	tabacina	saprophyte
ASV_36	unid_Phylum	unid_class	unid_order	unid_fam	unid_genus	unid_sp	unknown
ASV_360	unid_Phylum	unid_class	unid_order	unid_fam	unid_genus	unid_sp	unknown
ASV_361	Basidiomycota	Wallemiomycetes	Wallemiales	Wallemiaceae	Wallemia	muriae	animal commensal
ASV_362	Basidiomycota	Agaricomycetes	unid_Agaricomycetes_order	unid_Agaricomycetes_fam	unid_genus	unid_sp	unknown
ASV_363	unid_Phylum	unid_class	unid_order	unid_fam	unid_genus	unid_sp	unknown
ASV_364	unid_Phylum	unid_class	unid_order	unid_fam	unid_genus	unid_sp	unknown
ASV_365	Ascomycota	Dothideomycetes	Pleosporales	Corynesporascaceae	Corynespora	cassiicola	phytopathogen
ASV_366	unid_Phylum	unid_class	unid_order	unid_fam	unid_genus	unid_sp	unknown
ASV_369	Basidiomycota	Agaricomycetes	Cantharellales	unid_Cantharellales_fam	Sistotrema	sernanderi	saprophyte

ASV_37	Ascomycota	Sordariomycetes	Hypocreales	Nectriaceae	Fusarium	concentricum	phytopathogen
ASV_370	Basidiomycota	Agaricomycetes	Russulales	Stereaceae	Stereum	Stereum_sp	saprophyte
ASV_371	Basidiomycota	Exobasidiomycetes	Exobasidiales	Brachybasidiaceae	Meira	Meira_sp	insecticolous
ASV_375	Basidiomycota	Agaricomycetes	Polyporales	Meripilaceae	Meripilus	giganteus	saprophyte
ASV_380	Ascomycota	Sordariomycetes	Hypocreales	Ophiocordycipitaceae	Ophiocordyceps	retorta	insecticolous
ASV_381	Ascomycota	Sordariomycetes	Xylariales	Sporocadaceae	Neopestalotiopsis	saprophytica	phytopathogen
ASV_382	unid_Phylum	unid_class	unid_order	unid_fam	unid_genus	unid_sp	unknown
ASV_383	Basidiomycota	Cystobasidiomycetes	Erythrobasidiales	Erythrobasidiaceae	Bannoa	Bannoa_sp	epiphyte
ASV_387	unid_Phylum	unid_class	unid_order	unid_fam	unid_genus	unid_sp	unknown
ASV_392	Ascomycota	Sordariomycetes	Xylariales	Sporocadaceae	Neopestalotiopsis	Neopestalotiopsis_sp	phytopathogen
ASV_394	unid_Phylum	unid_class	unid_order	unid_fam	unid_genus	unid_sp	unknown
ASV_396	unid_Phylum	unid_class	unid_order	unid_fam	unid_genus	unid_sp	unknown
ASV_400	Basidiomycota	Wallemiomycetes	Wallemiales	Wallemiaceae	Wallemia	muriae	animal commensal
ASV_403	Basidiomycota	Agaricomycetes	unid_Agaricomycetes_order	unid_Agaricomycetes_fam	unid_genus	unid_sp	unknown
ASV_404	unid_Phylum	unid_class	unid_order	unid_fam	unid_genus	unid_sp	unknown
ASV_408	Basidiomycota	Wallemiomycetes	Wallemiales	Wallemiaceae	Wallemia	canadensis	animal commensal
ASV_409	unid_Phylum	unid_class	unid_order	unid_fam	unid_genus	unid_sp	unknown
ASV_414	Basidiomycota	Wallemiomycetes	Wallemiales	Wallemiaceae	Wallemia	muriae	animal commensal
ASV_415	Basidiomycota	Cystobasidiomycetes	Erythrobasidiales	Erythrobasidiaceae	Erythrobasidium	hasegawianum	epiphyte
ASV_418	Basidiomycota	Agaricomycetes	Sebacinales	unid_Sebacinales_fam	unid_genus	unid_sp	unknown
ASV_419	Basidiomycota	Agaricomycetes	Sebacinales	unid_Sebacinales_fam	unid_genus	unid_sp	unknown
ASV_42	Chytridiomycota	Spizellomycetes	Spizellomycetales	Powellomycetaceae	Geranomyces	variabilis	unknown
ASV_420	unid_Phylum	unid_class	unid_order	unid_fam	unid_genus	unid_sp	unknown
ASV_421	Ascomycota	Dothideomycetes	Botryosphaeriales	Botryosphaeriaceae	Diplodia	pseudoseriata	phytopathogen
ASV_425	Basidiomycota	Malasseziomycetes	Malasseziales	Malasseziaceae	Malassezia	globosa	animal commensal
ASV_428	Ascomycota	Eurotiomycetes	Chaetothyriales	Herpotrichiellaceae	Exophiala	exophialae	animal pathogen
ASV_431	unid_Phylum	unid_class	unid_order	unid_fam	unid_genus	unid_sp	unknown
ASV_433	unid_Phylum	unid_class	unid_order	unid_fam	unid_genus	unid_sp	unknown
ASV_434	unid_Phylum	unid_class	unid_order	unid_fam	unid_genus	unid_sp	unknown
ASV_435	unid_Phylum	unid_class	unid_order	unid_fam	unid_genus	unid_sp	unknown
ASV_436	Ascomycota	Eurotiomycetes	Eurotiales	Aspergillaceae	Aspergillus	glabripes	saprophyte
ASV_437	Basidiomycota	Pucciniomycetes	Septobasidiales	Septobasidiaceae	Septobasidium	Septobasidium_sp	insecticolous
ASV_438	Ascomycota	Dothideomycetes	Capnodiales	Cladosporiaceae	Cladosporium	Cladosporium_sp	saprophyte
ASV_44	Basidiomycota	Wallemiomycetes	Wallemiales	Wallemiaceae	Wallemia	muriae	animal commensal
ASV_440	Basidiomycota	Agaricomycetes	Cantharellales	unid_Cantharellales_fam	Sistotrema	sernanderi	saprophyte
ASV_442	Basidiomycota	Agaricomycetes	Russulales	Stereaceae	Stereum	Stereum_sp	saprophyte
ASV_447	Basidiomycota	Malasseziomycetes	Malasseziales	Malasseziaceae	Malassezia	arunalokei	animal commensal
ASV_448	Ascomycota	Sordariomycetes	Hypocreales	Nectriaceae	Albonectria	rigidiuscula	saprophyte
ASV_449	unid_Phylum	unid_class	unid_order	unid_fam	unid_genus	unid_sp	unknown
ASV_45	Ascomycota	Sordariomycetes	Hypocreales	Nectriaceae	Fusarium	concentricum	phytopathogen
ASV_450	Basidiomycota	Agaricomycetes	Hymenochaetales	Hymenochaetaceae	Hymenochaetopsis	tabacina	saprophyte
ASV_451	Basidiomycota	Pucciniomycetes	Septobasidiales	Septobasidiaceae	Septobasidium	ramorum	insecticolous
ASV_455	Ascomycota	Sordariomycetes	Hypocreales	Nectriaceae	Fusarium	concentricum	phytopathogen
ASV_457	Ascomycota	Dothideomycetes	Botryosphaeriales	Phyllostictaceae	Phyllosticta	catimbauensis	phytopathogen
ASV_46	Basidiomycota	Wallemiomycetes	Wallemiales	Wallemiaceae	Wallemia	muriae	animal commensal
ASV_463	Basidiomycota	Agaricomycetes	Cantharellales	unid_Cantharellales_fam	Multiclavula	Multiclavula_sp	lichen
ASV_464	unid_Phylum	unid_class	unid_order	unid_fam	unid_genus	unid_sp	unknown
ASV_465	Ascomycota	Saccharomycetes	Saccharomycetales	Debaryomycetaceae	Debaryomyces	Debaryomyces_sp	animal commensal
ASV_466	unid_Phylum	unid_class	unid_order	unid_fam	unid_genus	unid_sp	unknown
ASV_467	unid_Phylum	unid_class	unid_order	unid_fam	unid_genus	unid_sp	unknown

ASV_468	Ascomycota	Sordariomycetes	Hypocreales	Nectriaceae	Albonectria	rigidiuscula	saprophyte
ASV_469	unid_Phylum	unid_class	unid_order	unid_fam	unid_genus	unid_sp	unknown
ASV_47	Basidiomycota	unid_Basidiomycota_class	unid_Basidiomycota_order	unid_Basidiomycota_fam	unid_genus	unid_sp	unknown
ASV_472	Ascomycota	Sordariomycetes	Glomerellales	Glomerellaceae	Colletotrichum	ignotum	phytopathogen
ASV_473	Basidiomycota	Wallemiomycetes	Wallemiales	Wallemiaceae	Wallemia	muriae	animal commensal
ASV_474	unid_Phylum	unid_class	unid_order	unid_fam	unid_genus	unid_sp	unknown
ASV_475	unid_Phylum	unid_class	unid_order	unid_fam	unid_genus	unid_sp	unknown
ASV_477	Basidiomycota	Pucciniomycetes	Septobasidiales	Septobasidiaceae	Septobasidium	ramorum	insecticolous
ASV_478	unid_Phylum	unid_class	unid_order	unid_fam	unid_genus	unid_sp	unknown
ASV_479	Ascomycota	Dothideomycetes	unid_Dothideomycetes_order	unid_Dothideomycetes_fam	unid_genus	unid_sp	unknown
ASV_483	unid_Phylum	unid_class	unid_order	unid_fam	unid_genus	unid_sp	unknown
ASV_486	unid_Phylum	unid_class	unid_order	unid_fam	unid_genus	unid_sp	unknown
ASV_487	Ascomycota	Eurotiomycetes	Eurotiales	Aspergillaceae	Aspergillus	restrictus	saprophyte
ASV_488	Ascomycota	Sordariomycetes	Hypocreales	Hypocreaceae	Trichoderma	lixii	fungicolous
ASV_490	Chytridiomycota	Spizellomycetes	Spizellomycetales	Powellomycetaceae	Geranomyces	variabilis	unknown
ASV_491	unid_Phylum	unid_class	unid_order	unid_fam	unid_genus	unid_sp	unknown
ASV_495	Basidiomycota	Tremellomycetes	Tremellales	Tremellaceae	Cryptococcus	Cryptococcus_sp	animal commensal
ASV_5	unid_Phylum	unid_class	unid_order	unid_fam	unid_genus	unid_sp	unknown
ASV_501	unid_Phylum	unid_class	unid_order	unid_fam	unid_genus	unid_sp	unknown
ASV_502	unid_Phylum	unid_class	unid_order	unid_fam	unid_genus	unid_sp	unknown
ASV_504	unid_Phylum	unid_class	unid_order	unid_fam	unid_genus	unid_sp	unknown
ASV_505	unid_Phylum	unid_class	unid_order	unid_fam	unid_genus	unid_sp	unknown
ASV_506	Ascomycota	Eurotiomycetes	Chaetothyriales	Trichomeriaceae	unid_genus	unid_sp	unknown
ASV_508	Basidiomycota	Agaricomycetes	Agaricales	Psathyrellaceae	Coprinellus	dissemisptus	saprophyte
ASV_509	Ascomycota	Eurotiomycetes	Eurotiales	Aspergillaceae	Aspergillus	conicus	saprophyte
ASV_51	Ascomycota	unid_Ascomycota_class	unid_Ascomycota_order	unid_Ascomycota_fam	unid_genus	unid_sp	unknown
ASV_512	Basidiomycota	Agaricomycetes	Trechisporales	Hydnodontaceae	Trechispora	Trechispora_sp	saprophyte
ASV_515	unid_Phylum	unid_class	unid_order	unid_fam	unid_genus	unid_sp	unknown
ASV_52	Basidiomycota	Tremellomycetes	Cystofilobasidiales	Cystofilobasidiaceae	Cystofilobasidium	capitatum	animal commensal
ASV_521	Ascomycota	Eurotiomycetes	Eurotiales	Aspergillaceae	Aspergillus	penicillioides	saprophyte
ASV_523	Ascomycota	Dothideomycetes	Pleosporales	Thyridariaceae	Roussoella	Roussoella_sp	saprophyte
ASV_525	unid_Phylum	unid_class	unid_order	unid_fam	unid_genus	unid_sp	unknown
ASV_527	unid_Phylum	unid_class	unid_order	unid_fam	unid_genus	unid_sp	unknown
ASV_528	Ascomycota	Sordariomycetes	Xylariales	Xylariaceae	Annulohypoxylon	purpureopigmentum	saprophyte
ASV_529	unid_Phylum	unid_class	unid_order	unid_fam	unid_genus	unid_sp	unknown
ASV_530	Ascomycota	Eurotiomycetes	Eurotiales	Aspergillaceae	Aspergillus	conicus	saprophyte
ASV_532	Ascomycota	Laboulbeniomycetes	Pyxidiophorales	Pyxidiophoraceae	Pyxidiophora	arvernensis	fungicolous
ASV_537	unid_Phylum	unid_class	unid_order	unid_fam	unid_genus	unid_sp	unknown
ASV_544	unid_Phylum	unid_class	unid_order	unid_fam	unid_genus	unid_sp	unknown
ASV_545	Basidiomycota	Agaricostilbomycetes	Agaricostilbales	Chionosphaeraceae	unid_genus	unid_sp	unknown
ASV_546	Basidiomycota	Agaricomycetes	Polyporales	Phanerochaetaceae	Hyphodermella	Hyphodermella_sp	saprophyte
ASV_547	Basidiomycota	Agaricomycetes	Cantharellales	unid_Cantharellales_fam	Multiclavula	Multiclavula_sp	lichen
ASV_548	Ascomycota	Dothideomycetes	Pleosporales	Thyridariaceae	unid_genus	unid_sp	unknown
ASV_549	unid_Phylum	unid_class	unid_order	unid_fam	unid_genus	unid_sp	unknown
ASV_553	Basidiomycota	Agaricomycetes	unid_Agaricomycetes_order	unid_Agaricomycetes_fam	unid_genus	unid_sp	unknown
ASV_555	Basidiomycota	Pucciniomycetes	Septobasidiales	Septobasidiaceae	Septobasidium	Septobasidium_sp	insecticolous
ASV_556	unid_Phylum	unid_class	unid_order	unid_fam	unid_genus	unid_sp	unknown
ASV_558	unid_Phylum	unid_class	unid_order	unid_fam	unid_genus	unid_sp	unknown
ASV_56	Chytridiomycota	Spizellomycetes	Spizellomycetales	Powellomycetaceae	Geranomyces	variabilis	unknown
ASV_565	Ascomycota	unid_Ascomycota_class	unid_Ascomycota_order	unid_Ascomycota_fam	unid_genus	unid_sp	unknown

ASV_566	unid_Phylum	unid_class	unid_order	unid_fam	unid_genus	unid_sp	unknown
ASV_567	unid_Phylum	unid_class	unid_order	unid_fam	unid_genus	unid_sp	unknown
ASV_568	Basidiomycota	Malasseziomycetes	Malasseziales	Malasseziaceae	Malassezia	arunalokei	animal commensal
ASV_569	unid_Phylum	unid_class	unid_order	unid_fam	unid_genus	unid_sp	unknown
ASV_57	Ascomycota	Dothideomycetes	Pleosporales	unid_Pleosporales_fam	unid_genus	unid_sp	unknown
ASV_571	unid_Phylum	unid_class	unid_order	unid_fam	unid_genus	unid_sp	unknown
ASV_572	Basidiomycota	Pucciniomycetes	Septobasidiales	Septobasidiaceae	Septobasidium	Septobasidium_sp	insecticolous
ASV_575	unid_Phylum	unid_class	unid_order	unid_fam	unid_genus	unid_sp	unknown
ASV_577	Basidiomycota	Malasseziomycetes	Malasseziales	Malasseziaceae	Malassezia	globosa	animal commensal
ASV_58	Ascomycota	Saccharomycetes	Saccharomycetales	unid_Saccharomycetales_fam	unid_genus	unid_sp	unknown
ASV_581	Ascomycota	Dothideomycetes	Pleosporales	Didymellaceae	Neoascochyta	argentisp	phytopathogen
ASV_587	Ascomycota	Dothideomycetes	Capnodiales	Mycosphaerellaceae	Mycosphaerella	tassiana	phytopathogen
ASV_588	unid_Phylum	unid_class	unid_order	unid_fam	unid_genus	unid_sp	unknown
ASV_589	Basidiomycota	Agaricomycetes	Russulales	Lachnocladiaceae	Asterostroma	cervicolor	saprophyte
ASV_59	Ascomycota	Laboulbeniomycetes	Pyxidiophorales	Pyxidiophoraceae	Pyxidiophora	arvernensis	fungicolous
ASV_590	Ascomycota	Dothideomycetes	Pleosporales	unid_Pleosporales_fam	unid_genus	unid_sp	unknown
ASV_593	Basidiomycota	Agaricomycetes	Polyporales	Meripilaceae	Meripilus	giganteus	saprophyte
ASV_596	Ascomycota	Laboulbeniomycetes	Pyxidiophorales	Pyxidiophoraceae	Pyxidiophora	arvernensis	fungicolous
ASV_6	Ascomycota	Sordariomycetes	Hypocreales	Nectriaceae	Fusarium	waltergamsii	saprophyte
ASV_60	Ascomycota	Laboulbeniomycetes	Pyxidiophorales	Pyxidiophoraceae	Pyxidiophora	arvernensis	fungicolous
ASV_601	Ascomycota	Dothideomycetes	Pleosporales	Didymellaceae	unid_genus	unid_sp	phytopathogen
ASV_602	Basidiomycota	Agaricomycetes	Trechisporales	Hydnodontaceae	Trechispora	Trechispora_sp	saprophyte
ASV_603	Basidiomycota	Cystobasidiomycetes	Erythrobasidiales	Erythrobasidiaceae	Bannoa	Bannoa_sp	epiphyte
ASV_604	unid_Phylum	unid_class	unid_order	unid_fam	unid_genus	unid_sp	unknown
ASV_605	Ascomycota	Sordariomycetes	Diaporthales	Diaporthaceae	Diaporthe	Diaporthe_sp	phytopathogen
ASV_611	Basidiomycota	Cystobasidiomycetes	Erythrobasidiales	unid_Erythrobasidiales_fam	unid_genus	unid_sp	unknown
ASV_612	Basidiomycota	Agaricomycetes	Polyporales	Meruliaceae	Phlebia	tremellosa	saprophyte
ASV_615	unid_Phylum	unid_class	unid_order	unid_fam	unid_genus	unid_sp	unknown
ASV_619	Basidiomycota	Agaricomycetes	Atheliales	Atheliaceae	Athelia	decipiens	saprophyte
ASV_62	Ascomycota	Sordariomycetes	Glomerellales	Glomerellaceae	Colletotrichum	cairnsense	phytopathogen
ASV_622	Ascomycota	Sordariomycetes	Hypocreales	Cordycipitaceae	Simplicillium	lanosoniveum	fungicolous
ASV_628	Basidiomycota	Agaricomycetes	Cantharellales	Ceratobasidiaceae	Thanatephorus	cucumeris	phytopathogen
ASV_630	Basidiomycota	Agaricostilbomycetes	Agaricostilbales	Chionosphaeraceae	Kurtzmanomyces	Kurtzmanomyces_sp	unknown
ASV_631	Basidiomycota	Agaricomycetes	Agaricales	Psathyrellaceae	Coprinellus	dissemisptus	saprophyte
ASV_633	Basidiomycota	Cystobasidiomycetes	Erythrobasidiales	Erythrobasidiaceae	Bannoa	ogasawarensis	epiphyte
ASV_634	unid_Phylum	unid_class	unid_order	unid_fam	unid_genus	unid_sp	unknown
ASV_635	unid_Phylum	unid_class	unid_order	unid_fam	unid_genus	unid_sp	unknown
ASV_639	Basidiomycota	Agaricomycetes	Polyporales	Polyporaceae	Trametes	versicolor	saprophyte
ASV_643	Basidiomycota	Agaricomycetes	Polyporales	Steccherinaceae	unid_genus	unid_sp	saprophyte
ASV_644	unid_Phylum	unid_class	unid_order	unid_fam	unid_genus	unid_sp	unknown
ASV_646	unid_Phylum	unid_class	unid_order	unid_fam	unid_genus	unid_sp	unknown
ASV_647	Ascomycota	Dothideomycetes	Pleosporales	Pleosporaceae	Curvularia	sorghina	phytopathogen
ASV_650	Basidiomycota	Malasseziomycetes	Malasseziales	Malasseziaceae	Malassezia	globosa	animal commensal
ASV_651	Basidiomycota	Agaricomycetes	Polyporales	Fomitopsidaceae	Laetiporus	gilbertsonii	saprophyte
ASV_652	unid_Phylum	unid_class	unid_order	unid_fam	unid_genus	unid_sp	unknown
ASV_657	unid_Phylum	unid_class	unid_order	unid_fam	unid_genus	unid_sp	unknown
ASV_658	unid_Phylum	unid_class	unid_order	unid_fam	unid_genus	unid_sp	unknown
ASV_66	Basidiomycota	Malasseziomycetes	Malasseziales	Malasseziaceae	Malassezia	arunalokei	animal commensal
ASV_661	Basidiomycota	Cystobasidiomycetes	Erythrobasidiales	Erythrobasidiaceae	unid_genus	unid_sp	unknown
ASV_665	Basidiomycota	Wallemiomycetes	Wallemiales	Wallemiaceae	Wallemia	Wallemia_sp	animal commensal

ASV_67	Basidiomycota	Malasseziomycetes	Malasseziales	Malasseziaceae	Malassezia	restricta	animal commensal
ASV_670	unid_Phylum	unid_class	unid_order	unid_fam	unid_genus	unid_sp	unknown
ASV_671	Basidiomycota	unid_Basidiomycota_class	unid_Basidiomycota_order	unid_Basidiomycota_fam	unid_genus	unid_sp	unknown
ASV_674	Ascomycota	Dothideomycetes	Capnodiales	Cladosporiaceae	Cladosporium	Cladosporium_sp	saprophyte
ASV_678	Ascomycota	Sordariomycetes	Hypocreales	Cordycipitaceae	Engyodontium	album	unknown
ASV_679	Basidiomycota	Agaricomycetes	Polyporales	Polyporaceae	Trametes	versicolor	saprophyte
ASV_68	Ascomycota	Laboulbeniomycetes	Pyxidiophorales	Pyxidiophoraceae	Pyxidiophora	arvernensis	fungicolous
ASV_680	unid_Phylum	unid_class	unid_order	unid_fam	unid_genus	unid_sp	unknown
ASV_683	Basidiomycota	Malasseziomycetes	Malasseziales	Malasseziaceae	Malassezia	arunalokei	animal commensal
ASV_684	unid_Phylum	unid_class	unid_order	unid_fam	unid_genus	unid_sp	unknown
ASV_685	Basidiomycota	Malasseziomycetes	Malasseziales	Malasseziaceae	Malassezia	globosa	animal commensal
ASV_686	unid_Phylum	unid_class	unid_order	unid_fam	unid_genus	unid_sp	unknown
ASV_69	Basidiomycota	Tremellomycetes	Cystofilobasidiales	Cystofilobasidiaceae	Cystofilobasidium	capitatum	animal commensal
ASV_690	Ascomycota	Sordariomycetes	Hypocreales	Nectriaceae	Thelonectria	jungneri	saprophyte
ASV_691	Ascomycota	Sordariomycetes	Hypocreales	Nectriaceae	Gliocladiopsis	elghollii	unknown
ASV_692	Ascomycota	Laboulbeniomycetes	Pyxidiophorales	Pyxidiophoraceae	Pyxidiophora	arvernensis	fungicolous
ASV_702	Basidiomycota	Agaricomycetes	Polyporales	Phanerochaetaceae	Hyphodermella	Hyphodermella_sp	saprophyte
ASV_703	Basidiomycota	Malasseziomycetes	Malasseziales	Malasseziaceae	Malassezia	arunalokei	animal commensal
ASV_704	Ascomycota	Sordariomycetes	Hypocreales	Nectriaceae	Fusarium	fujikuroi	phytopathogen
ASV_705	Basidiomycota	Ustilaginomycetes	Ustilaginales	Ustilaginaceae	Moesziomyces	aphidis	phytopathogen
ASV_706	unid_Phylum	unid_class	unid_order	unid_fam	unid_genus	unid_sp	unknown
ASV_709	unid_Phylum	unid_class	unid_order	unid_fam	unid_genus	unid_sp	unknown
ASV_710	unid_Phylum	unid_class	unid_order	unid_fam	unid_genus	unid_sp	unknown
ASV_711	Basidiomycota	Agaricomycetes	Sebacinales	unid_Sebacinales_fam	unid_genus	unid_sp	unknown
ASV_715	Basidiomycota	Tremellomycetes	Tremellales	Rhynchogastremataceae	Papiliotrema	leoncinii	epiphyte
ASV_716	Basidiomycota	Agaricomycetes	Polyporales	Polyporaceae	unid_genus	unid_sp	saprophyte
ASV_717	Ascomycota	Sordariomycetes	Hypocreales	Nectriaceae	Thelonectria	diademata	saprophyte
ASV_719	Basidiomycota	Agaricomycetes	Hymenochaetales	unid_Hymenochaetales_fam	Trichaptum	biforme	saprophyte
ASV_720	unid_Phylum	unid_class	unid_order	unid_fam	unid_genus	unid_sp	unknown
ASV_721	unid_Phylum	unid_class	unid_order	unid_fam	unid_genus	unid_sp	unknown
ASV_722	Basidiomycota	Agaricomycetes	Polyporales	Fomitopsidaceae	Laetiporus	gilbertsonii	saprophyte
ASV_723	unid_Phylum	unid_class	unid_order	unid_fam	unid_genus	unid_sp	unknown
ASV_725	Basidiomycota	unid_Basidiomycota_class	unid_Basidiomycota_order	unid_Basidiomycota_fam	unid_genus	unid_sp	unknown
ASV_726	unid_Phylum	unid_class	unid_order	unid_fam	unid_genus	unid_sp	unknown
ASV_727	unid_Phylum	unid_class	unid_order	unid_fam	unid_genus	unid_sp	unknown
ASV_729	unid_Phylum	unid_class	unid_order	unid_fam	unid_genus	unid_sp	unknown
ASV_731	Ascomycota	Eurotiomycetes	Chaetothyriales	unid_Chaetothyriales_fam	Neostrelitziana	acaciigena	unknown
ASV_732	unid_Phylum	unid_class	unid_order	unid_fam	unid_genus	unid_sp	unknown
ASV_734	Ascomycota	Sordariomycetes	Hypocreales	Nectriaceae	Albonectria	rigidiuscula	saprophyte
ASV_737	Ascomycota	Dothideomycetes	Pleosporales	unid_Pleosporales_fam	unid_genus	unid_sp	unknown
ASV_738	Basidiomycota	Agaricomycetes	unid_Agaricomycetes_order	unid_Agaricomycetes_fam	unid_genus	unid_sp	unknown
ASV_74	Ascomycota	Dothideomycetes	Pleosporales	unid_Pleosporales_fam	unid_genus	unid_sp	unknown
ASV_743	Basidiomycota	Agaricomycetes	unid_Agaricomycetes_order	unid_Agaricomycetes_fam	unid_genus	unid_sp	unknown
ASV_744	Basidiomycota	Agaricomycetes	Sebacinales	unid_Sebacinales_fam	unid_genus	unid_sp	unknown
ASV_745	unid_Phylum	unid_class	unid_order	unid_fam	unid_genus	unid_sp	unknown
ASV_747	unid_Phylum	unid_class	unid_order	unid_fam	unid_genus	unid_sp	unknown
ASV_748	Ascomycota	Dothideomycetes	Pleosporales	Didymellaceae	Neoascochyta	argentisp	phytopathogen
ASV_749	Ascomycota	Laboulbeniomycetes	Pyxidiophorales	Pyxidiophoraceae	Pyxidiophora	arvernensis	fungicolous
ASV_75	Basidiomycota	Cystobasidiomycetes	unid_Cystobasidiomycetes_order	Symmetrosporaceae	Symmetrospora	Symmetrospora_sp	saprophyte
ASV_752	unid_Phylum	unid_class	unid_order	unid_fam	unid_genus	unid_sp	unknown

ASV_756	Basidiomycota	Pucciniomycetes	Septobasidiales	Septobasidiaceae	unid_genus	unid_sp	unknown
ASV_757	Basidiomycota	Agaricostilbomycetes	Agaricostilbales	Chionosphaeraceae	unid_genus	unid_sp	unknown
ASV_758	Basidiomycota	Agaricomycetes	Geastrales	Geastraceae	Geastrum	rubropusillum	saprophyte
ASV_759	Ascomycota	Dothideomycetes	Capnodiales	Mycosphaerellaceae	unid_genus	unid_sp	phytopathogen
ASV_76	unid_Phylum	unid_class	unid_order	unid_fam	unid_genus	unid_sp	unknown
ASV_761	Basidiomycota	Agaricomycetes	unid_Agaricomycetes_order	unid_Agaricomycetes_fam	unid_genus	unid_sp	unknown
ASV_762	unid_Phylum	unid_class	unid_order	unid_fam	unid_genus	unid_sp	unknown
ASV_763	unid_Phylum	unid_class	unid_order	unid_fam	unid_genus	unid_sp	unknown
ASV_768	Basidiomycota	Agaricomycetes	Polyporales	Phanerochaetaceae	Hyphodermella	Hyphodermella_sp	saprophyte
ASV_769	Ascomycota	Dothideomycetes	Pleosporales	Pleosporaceae	Alternaria	betae-kenyensis	saprophyte
ASV_77	Ascomycota	Sordariomycetes	Glomerellales	Glomerellaceae	Colletotrichum	cairnsense	phytopathogen
ASV_771	Basidiomycota	Wallemiomycetes	Wallemiales	Wallemiaceae	Wallemia	muriae	animal commensal
ASV_772	Basidiomycota	Agaricomycetes	Polyporales	Meruliaceae	Phlebia	radiata	saprophyte
ASV_773	unid_Phylum	unid_class	unid_order	unid_fam	unid_genus	unid_sp	unknown
ASV_774	Ascomycota	Sordariomycetes	Hypocreales	unid_Hypocreales_fam	Acremonium	brachypenium	unknown
ASV_775	unid_Phylum	unid_class	unid_order	unid_fam	unid_genus	unid_sp	unknown
ASV_779	unid_Phylum	unid_class	unid_order	unid_fam	unid_genus	unid_sp	unknown
ASV_78	Basidiomycota	Cystobasidiomycetes	Cystobasidiales	Cystobasidiaceae	Occultifur	Occultifur_sp	fungicolous
ASV_783	unid_Phylum	unid_class	unid_order	unid_fam	unid_genus	unid_sp	unknown
ASV_787	Basidiomycota	Agaricomycetes	Atheliales	Atheliaceae	Athelia	decipiens	saprophyte
ASV_795	Basidiomycota	Agaricomycetes	Polyporales	Polyporaceae	Trametes	versicolor	saprophyte
ASV_796	Basidiomycota	Malasseziomycetes	Malasseziales	Malasseziaceae	Malassezia	restricta	animal commensal
ASV_797	unid_Phylum	unid_class	unid_order	unid_fam	unid_genus	unid_sp	unknown
ASV_798	unid_Phylum	unid_class	unid_order	unid_fam	unid_genus	unid_sp	unknown
ASV_799	unid_Phylum	unid_class	unid_order	unid_fam	unid_genus	unid_sp	unknown
ASV_8	Ascomycota	Laboulbeniomycetes	Pyxidiophorales	Pyxidiophoraceae	Pyxidiophora	arvernensis	fungicolous
ASV_80	Ascomycota	Eurotiomycetes	Eurotiales	Aspergillaceae	Aspergillus	glabripes	saprophyte
ASV_800	Basidiomycota	Cystobasidiomycetes	unid_Cystobasidiomycetes_order	Symmetrosporaceae	Symmetrospora	Symmetrospora_sp	saprophyte
ASV_801	unid_Phylum	unid_class	unid_order	unid_fam	unid_genus	unid_sp	unknown
ASV_802	unid_Phylum	unid_class	unid_order	unid_fam	unid_genus	unid_sp	unknown
ASV_808	Basidiomycota	Agaricostilbomycetes	Agaricostilbales	Chionosphaeraceae	Kurtzmanomyces	Kurtzmanomyces_sp	unknown
ASV_809	Ascomycota	unid_Ascomycota_class	unid_Ascomycota_order	unid_Ascomycota_fam	Tricladiella	pluvialis	saprophyte
ASV_81	Basidiomycota	Malasseziomycetes	Malasseziales	Malasseziaceae	Malassezia	globosa	animal commensal
ASV_810	Basidiomycota	Wallemiomycetes	Wallemiales	Wallemiaceae	Wallemia	muriae	animal commensal
ASV_814	Basidiomycota	unid_Basidiomycota_class	unid_Basidiomycota_order	unid_Basidiomycota_fam	unid_genus	unid_sp	unknown
ASV_815	unid_Phylum	unid_class	unid_order	unid_fam	unid_genus	unid_sp	unknown
ASV_817	Basidiomycota	Agaricomycetes	Polyporales	Phanerochaetaceae	Bjerkandera	adusta	saprophyte
ASV_818	unid_Phylum	unid_class	unid_order	unid_fam	unid_genus	unid_sp	unknown
ASV_82	unid_Phylum	unid_class	unid_order	unid_fam	unid_genus	unid_sp	unknown
ASV_821	Ascomycota	Eurotiomycetes	Eurotiales	Aspergillaceae	Aspergillus	penicillioides	saprophyte
ASV_822	Ascomycota	Sordariomycetes	Hypocreales	Cordycipitaceae	Cordyceps	javanica	insecticolous
ASV_826	Basidiomycota	Agaricostilbomycetes	Agaricostilbales	Chionosphaeraceae	unid_genus	unid_sp	unknown
ASV_827	Basidiomycota	Agaricomycetes	Sebacinales	unid_Sebacinales_fam	unid_genus	unid_sp	unknown
ASV_838	Basidiomycota	Agaricostilbomycetes	Agaricostilbales	unid_Agaricostilbales_fam	unid_genus	unid_sp	unknown
ASV_839	Basidiomycota	Wallemiomycetes	Wallemiales	Wallemiaceae	Wallemia	canadensis	animal commensal
ASV_84	Basidiomycota	Agaricomycetes	unid_Agaricomycetes_order	unid_Agaricomycetes_fam	unid_genus	unid_sp	unknown
ASV_840	Basidiomycota	Agaricostilbomycetes	Agaricostilbales	Chionosphaeraceae	unid_genus	unid_sp	unknown
ASV_841	Basidiomycota	Agaricomycetes	unid_Agaricomycetes_order	unid_Agaricomycetes_fam	unid_genus	unid_sp	unknown
ASV_842	Basidiomycota	Agaricomycetes	Polyporales	Fomitopsidaceae	Laetiporus	gilbertsonii	saprophyte
ASV_843	Ascomycota	Dothideomycetes	Pleosporales	Pleosporaceae	Curvularia	sorghina	phytopathogen

ASV_847	Ascomycota	Dothideomycetes	Pleosporales	unid_Pleosporales_fam	Nigrograna	cangshanensis	saprophyte
ASV_848	Basidiomycota	unid_Basidiomycota_class	unid_Basidiomycota_order	unid_Basidiomycota_fam	unid_genus	unid_sp	unknown
ASV_85	Ascomycota	Laboulbeniomycetes	Pyxidiophorales	Pyxidiophoraceae	Pyxidiophora	arvernensis	fungicolous
ASV_852	Basidiomycota	Cystobasidiomycetes	unid_Cystobasidiomycetes_order	Symmetrosporaceae	Symmetrospora	Symmetrospora_sp	saprophyte
ASV_858	Ascomycota	Dothideomycetes	Capnodiales	Cladosporiaceae	Cladosporium	halotolerans	saprophyte
ASV_859	unid_Phylum	unid_class	unid_order	unid_fam	unid_genus	unid_sp	unknown
ASV_861	Basidiomycota	Malasseziomycetes	Malasseziales	Malasseziaceae	Malassezia	restricta	animal commensal
ASV_863	Basidiomycota	unid_Basidiomycota_class	unid_Basidiomycota_order	unid_Basidiomycota_fam	unid_genus	unid_sp	unknown
ASV_864	Ascomycota	Saccharomycetes	Saccharomycetales	Debaryomycetaceae	Debaryomyces	nepalensis	animal commensal
ASV_865	unid_Phylum	unid_class	unid_order	unid_fam	unid_genus	unid_sp	unknown
ASV_866	Basidiomycota	Agaricomycetes	Agaricales	Psathyrellaceae	Coprinellus	dissemisptus	saprophyte
ASV_867	unid_Phylum	unid_class	unid_order	unid_fam	unid_genus	unid_sp	unknown
ASV_870	unid_Phylum	unid_class	unid_order	unid_fam	unid_genus	unid_sp	unknown
ASV_871	unid_Phylum	unid_class	unid_order	unid_fam	unid_genus	unid_sp	unknown
ASV_873	unid_Phylum	unid_class	unid_order	unid_fam	unid_genus	unid_sp	unknown
ASV_876	unid_Phylum	unid_class	unid_order	unid_fam	unid_genus	unid_sp	unknown
ASV_877	unid_Phylum	unid_class	unid_order	unid_fam	unid_genus	unid_sp	unknown
ASV_88	Ascomycota	Sordariomycetes	Hypocreales	Nectriaceae	Albonectria	rigidiuscula	saprophyte
ASV_880	Ascomycota	Eurotiomycetes	Eurotiales	Aspergillaceae	Penicillium	Penicillium_sp	saprophyte
ASV_881	Basidiomycota	Agaricomycetes	Agaricales	unid_Agaricales_fam	Chondrostereum	Chondrostereum_sp	saprophyte
ASV_884	Basidiomycota	Tremellomycetes	Tremellales	Rhynchogastremataceae	Papiliotrema	leoncinii	epiphyte
ASV_885	unid_Phylum	unid_class	unid_order	unid_fam	unid_genus	unid_sp	unknown
ASV_886	Basidiomycota	Agaricomycetes	Agaricales	Psathyrellaceae	Coprinellus	dissemisptus	saprophyte
ASV_89	Basidiomycota	Malasseziomycetes	Malasseziales	Malasseziaceae	Malassezia	restricta	animal commensal
ASV_893	Ascomycota	Dothideomycetes	unid_Dothideomycetes_order	unid_Dothideomycetes_fam	unid_genus	unid_sp	unknown
ASV_895	unid_Phylum	unid_class	unid_order	unid_fam	unid_genus	unid_sp	unknown
ASV_896	Basidiomycota	Agaricomycetes	Polyporales	Polyporaceae	Trametes	versicolor	saprophyte
ASV_9	unid_Phylum	unid_class	unid_order	unid_fam	unid_genus	unid_sp	unknown
ASV_900	unid_Phylum	unid_class	unid_order	unid_fam	unid_genus	unid_sp	unknown
ASV_907	Ascomycota	Sordariomycetes	Xylariales	Sporocadaceae	unid_genus	unid_sp	unknown
ASV_908	Basidiomycota	Pucciniomycetes	Septobasidiales	Septobasidiaceae	unid_genus	unid_sp	unknown
ASV_909	unid_Phylum	unid_class	unid_order	unid_fam	unid_genus	unid_sp	unknown
ASV_91	Basidiomycota	Exobasidiomycetes	Exobasidiales	Brachybasidiaceae	Meira	Meira_sp	insecticolous
ASV_910	Basidiomycota	Malasseziomycetes	Malasseziales	Malasseziaceae	Malassezia	arunalokei	animal commensal
ASV_911	Basidiomycota	Pucciniomycetes	Septobasidiales	Septobasidiaceae	Septobasidium	Septobasidium_sp	insecticolous
ASV_914	Ascomycota	Eurotiomycetes	Eurotiales	Aspergillaceae	Aspergillus	penicillioides	saprophyte
ASV_915	unid_Phylum	unid_class	unid_order	unid_fam	unid_genus	unid_sp	unknown
ASV_916	Ascomycota	Dothideomycetes	unid_Dothideomycetes_order	unid_Dothideomycetes_fam	unid_genus	unid_sp	unknown
ASV_917	Ascomycota	unid_Ascomycota_class	unid_Ascomycota_order	unid_Ascomycota_fam	unid_genus	unid_sp	unknown
ASV_918	Ascomycota	Dothideomycetes	Capnodiales	Mycosphaerellaceae	unid_genus	unid_sp	phytopathogen
ASV_923	unid_Phylum	unid_class	unid_order	unid_fam	unid_genus	unid_sp	unknown
ASV_924	unid_Phylum	unid_class	unid_order	unid_fam	unid_genus	unid_sp	unknown
ASV_925	Basidiomycota	Exobasidiomycetes	Exobasidiales	unid_Exobasidiales_fam	unid_genus	unid_sp	phytopathogen
ASV_926	unid_Phylum	unid_class	unid_order	unid_fam	unid_genus	unid_sp	unknown
ASV_927	unid_Phylum	unid_class	unid_order	unid_fam	unid_genus	unid_sp	unknown
ASV_934	Basidiomycota	Agaricomycetes	Polyporales	Ganodermataceae	Ganoderma	Ganoderma_sp	saprophyte
ASV_94	unid_Phylum	unid_class	unid_order	unid_fam	unid_genus	unid_sp	unknown
ASV_941	unid_Phylum	unid_class	unid_order	unid_fam	unid_genus	unid_sp	unknown
ASV_942	Basidiomycota	Agaricomycetes	Cantharellales	unid_Cantharellales_fam	Multiclavula	Multiclavula_sp	lichen
ASV_943	unid_Phylum	unid_class	unid_order	unid_fam	unid_genus	unid_sp	unknown

ASV_944	Ascomycota	unid_Ascomycota_class	unid_Ascomycota_order	unid_Ascomycota_fam	unid_genus	unid_sp	unknown
ASV_946	Ascomycota	Dothideomycetes	Pleosporales	unid_Pleosporales_fam	unid_genus	unid_sp	unknown
ASV_947	Ascomycota	Dothideomycetes	Venturiales	Sympoventuriaceae	Ochroconis	cordanae	phytopathogen
ASV_949	unid_Phylum	unid_class	unid_order	unid_fam	unid_genus	unid_sp	unknown
ASV_95	unid_Phylum	unid_class	unid_order	unid_fam	unid_genus	unid_sp	unknown
ASV_950	Ascomycota	Eurotiomycetes	Chaetothyriales	Chaetothyriaceae	Camptophora	Camptophora_sp	saprophyte
ASV_951	Ascomycota	Dothideomycetes	Pleosporales	Halotthiaceae	Sulcosporium	Sulcosporium_sp	phytopathogen
ASV_952	Basidiomycota	Agaricomycetes	Cantharellales	Ceratobasidiaceae	Thanatephorus	cucumeris	phytopathogen
ASV_953	unid_Phylum	unid_class	unid_order	unid_fam	unid_genus	unid_sp	unknown
ASV_963	Basidiomycota	Wallemiomycetes	Wallemiales	Wallemiaceae	Wallemia	canadensis	animal commensal
ASV_964	unid_Phylum	unid_class	unid_order	unid_fam	unid_genus	unid_sp	unknown
ASV_965	Basidiomycota	Wallemiomycetes	Wallemiales	Wallemiaceae	Wallemia	canadensis	animal commensal
ASV_966	unid_Phylum	unid_class	unid_order	unid_fam	unid_genus	unid_sp	unknown
ASV_967	Basidiomycota	Agaricomycetes	Agaricales	unid_Agaricales_fam	Chondrostereum	Chondrostereum_sp	saprophyte
ASV_969	Ascomycota	Sordariomycetes	Diaporthales	Diaporthaceae	Diaporthe	cucurbitae	phytopathogen
ASV_970	Basidiomycota	Agaricostilbomycetes	Agaricostilbales	Kondoaceae	Kondoa	thailandica	epiphyte
ASV_971	unid_Phylum	unid_class	unid_order	unid_fam	unid_genus	unid_sp	unknown
ASV_972	Ascomycota	Laboulbeniomycetes	Pyxidiophorales	Pyxidiophoraceae	Pyxidiophora	arvernensis	fungicolous
ASV_976	Basidiomycota	Pucciniomycetes	Septobasidiales	Septobasidiaceae	unid_genus	unid_sp	unknown
ASV_977	Ascomycota	Eurotiomycetes	Chaetothyriales	Trichomeriaceae	Trichomerium	dioscoreae	epiphyte
ASV_978	Basidiomycota	Agaricomycetes	Polyporales	Phanerochaetaceae	Hyphodermella	Hyphodermella_sp	saprophyte
ASV_979	Basidiomycota	Pucciniomycetes	Septobasidiales	Septobasidiaceae	unid_genus	unid_sp	unknown
ASV_98	Ascomycota	Sordariomycetes	Hypocreales	Nectriaceae	Fusarium	concentricum	phytopathogen
ASV_980	unid_Phylum	unid_class	unid_order	unid_fam	unid_genus	unid_sp	unknown
ASV_981	unid_Phylum	unid_class	unid_order	unid_fam	unid_genus	unid_sp	unknown
ASV_984	Basidiomycota	Agaricomycetes	Hymenochaetales	Hymenochaetaceae	Hymenochaete	Hymenochaete_sp	saprophyte
ASV_985	unid_Phylum	unid_class	unid_order	unid_fam	unid_genus	unid_sp	unknown
ASV_986	Basidiomycota	Malasseziomycetes	Malasseziales	Malasseziaceae	Malassezia	arunalokei	animal commensal
ASV_987	unid_Phylum	unid_class	unid_order	unid_fam	unid_genus	unid_sp	unknown
ASV_988	Basidiomycota	Agaricomycetes	unid_Agaricomycetes_order	unid_Agaricomycetes_fam	unid_genus	unid_sp	unknown
ASV_989	Basidiomycota	Agaricomycetes	Cantharellales	Ceratobasidiaceae	unid_genus	unid_sp	unknown
ASV_995	Basidiomycota	Pucciniomycetes	unid_Pucciniomycetes_order	unid_Pucciniomycetes_fam	unid_genus	unid_sp	unknown
ASV_996	Basidiomycota	Agaricomycetes	Polyporales	Meruliaceae	Phlebia	tremellosa	saprophyte
ASV_997	Basidiomycota	Wallemiomycetes	Wallemiales	Wallemiaceae	Wallemia	muriae	animal commensal
ASV_998	Ascomycota	Saccharomycetes	Saccharomycetales	Debaryomycetaceae	Kurtzmaniella	Kurtzmaniella_sp	animal commensal
ASV_999	Basidiomycota	Agaricomycetes	unid_Agaricomycetes_order	unid_Agaricomycetes_fam	unid_genus	unid_sp	unknown

Table S4. Taxonomy and BLAST search results for the culturable fungi from feces of *E. alba* and fruits of *F. colubrinae*.

Isolate	Identification	Fruits or Bats	Sample origin	Matching accession	Identity
Fc01_4	*Colletotrichum fructicola*	F	Fc01	MZ854221	100%
Fc04_03	*Colletotrichum siamense*	F	Fc04	MZ605385	100%
Fc01_2	*Diaporthe sp.*	F	Fc01	MH465206	100%
Fc03_01	*Diaporthe cf. hongkongensis*	F	Fc03	MN862379	99%
Fc02_01	*Fusarium concentricum*	F	Fc02	MT914496	100%
Fc02_02	*Fusarium concentricum*	F	Fc02	MT914496	100%
Fc02_03	*Fusarium concentricum*	F	Fc02	MT914496	100%
Fc02_04	*Fusarium concentricum*	F	Fc02	MT914496	100%
Fc04_02	*Fusarium concentricum*	F	Fc04	MT914496	100%
Fc01_1	*Neopestalotiopsis saprophytica*	F	Fc01	MT576586	100%
Fc01_3	*Neopestalotiopsis saprophytica*	F	Fc01	MT576586	100%
Ea08_2	*Fusarium concentricum*	B	Ea08	MT914496	100%
Ea08_3	*Fusarium concentricum*	B	Ea08	MT914496	100%
Ea08_4	*Fusarium concentricum*	B	Ea08	MT914496	100%
Ea08_5	*Fusarium concentricum*	B	Ea08	MT914496	100%
Ea08_6	*Fusarium concentricum*	B	Ea08	MT914496	100%
Ea10_4	*Fusarium concentricum*	B	Ea10	MT914496	100%
Ea13_1	*Fusarium concentricum*	B	Ea13	MT914496	100%
Ea13_3	*Fusarium concentricum*	B	Ea13	MT914496	100%
Ea15_1	*Fusarium concentricum*	B	Ea15	MT914496	100%
Ea15_2	*Fusarium concentricum*	B	Ea15	MT914496	100%
Ea16_3	*Fusarium concentricum*	B	Ea16	MT914496	100%
Ea13_2	*Fusarium waltergamsii*	B	Ea13	NR159548	99%
Ea13_5	*Fusarium waltergamsii*	B	Ea13	NR159548	99%
Ea14_1	*Fusarium waltergamsii*	B	Ea14	NR159548	99%
Ea14_4	*Fusarium waltergamsii*	B	Ea14	NR159548	99%
Ea07_3	*Mucor irregularis*	B	Ea07	MZ423088	99%
Ea09_1	*Mucor irregularis*	B	Ea09	MZ423088	99%
Ea09_2	*Mucor irregularis*	B	Ea09	MZ423088	99%
Ea09_3	*Mucor irregularis*	B	Ea09	MZ423088	99%
Ea09_4	*Mucor irregularis*	B	Ea09	MZ423088	99%
Ea11_4	*Mucor irregularis*	B	Ea11	MZ423088	99%
Ea14_2	*Mucor irregularis*	B	Ea14	MZ423088	99%
Ea14_3	*Mucor irregularis*	B	Ea14	MZ423088	99%
Ea16_1	*Mucor irregularis*	B	Ea16	MZ423088	99%
Ea16_2	*Mucor irregularis*	B	Ea16	MZ423088	99%
Ea16_4	*Mucor irregularis*	B	Ea16	MZ423088	99%
Ea17_1	*Mucor irregularis*	B	Ea17	MZ423088	99%
Ea17_3	*Mucor irregularis*	B	Ea17	MZ423088	99%
Ea11_5	*Neopestalotiopsis saprophytica*	B	Ea11	MT576586	100%
Ea10_1	*Pseudopestalotiopsis simitheae*	B	Ea10	MW488215	100%
